# Risk of severe COVID-19 outcomes after autumn 2022 COVID-19 booster vaccinations: a pooled analysis of national prospective cohort studies involving 7.4 million adults in England, Northern Ireland, Scotland and Wales

**DOI:** 10.1016/j.lanepe.2023.100816

**Published:** 2023-12-12

**Authors:** Stuart Bedston, Fatima Almaghrabi, Lynsey Patterson, Utkarsh Agrawal, Lana Woolford, Sneha N. Anand, Mark Joy, Anna Crawford, Rosalind Goudie, Rachel Byford, Hoda Abbasizanjani, Deb Smith, Lynn Laidlaw, Ashley Akbari, Christopher Sullivan, Declan T. Bradley, Ronan A. Lyons, Simon de Lusignan, F.D. Richard Hobbs, Chris Robertson, Sir Aziz Sheikh, Ting Shi

**Affiliations:** a Usher Institute, Edinburgh Medical School, University of Edinburgh, Edinburgh, Scotland, UK; b Department of Mathematics and Statistics, University of Strathclyde, Glasgow, Scotland, UK; c Population Data Science, Swansea University Medical School, Faculty of Medicine, Health, and Life Science, Swansea University, Swansea, UK; d Centre for Public Health, Queen's University Belfast, Belfast, UK; e Nuffield Department of Primary Care Health Sciences, University of Oxford, Oxford, UK; f Public Health Agency, Belfast, UK; g Public Health Scotland, Glasgow, Scotland, UK

**Keywords:** COVID-19, Vaccine, Hospital admission, Vaccine breakthrough, Booster dose

## Abstract

**Background:**

UK COVID-19 vaccination policy has evolved to offering COVID-19 booster doses to individuals at increased risk of severe Illness from COVID-19. Building on our analyses of vaccine effectiveness of first, second and initial booster doses, we aimed to identify individuals at increased risk of severe outcomes (i.e., COVID-19 related hospitalisation or death) post the autumn 2022 booster dose.

**Methods:**

We undertook a national population-based cohort analysis across all four UK nations through linked primary care, vaccination, hospitalisation and mortality data. We included individuals who received autumn 2022 booster doses of BNT162b2 (Comirnaty) or mRNA-1273 (Spikevax) during the period September 1, 2022 to December 31, 2022 to investigate the risk of severe COVID-19 outcomes. Cox proportional hazard models were used to estimate adjusted hazard ratios (aHR) and 95% confidence intervals (CIs) for the association between demographic and clinical factors and severe COVID-19 outcomes after the autumn booster dose. Analyses were adjusted for age, sex, body mass index (BMI), deprivation, urban/rural areas and comorbidities. Stratified analyses were conducted by vaccine type. We then conducted a fixed-effect meta-analysis to combine results across the four UK nations.

**Findings:**

Between September 1, 2022 and December 31, 2022, 7,451,890 individuals ≥18 years received an autumn booster dose. 3500 had severe COVID-19 outcomes (2.9 events per 1000 person-years). Being male (male vs female, aHR 1.41 (1.32–1.51)), older adults (≥80 years vs 18–49 years; 10.43 (8.06–13.50)), underweight (BMI <18.5 vs BMI 25.0–29.9; 2.94 (2.51–3.44)), those with comorbidities (≥5 comorbidities vs none; 9.45 (8.15–10.96)) had a higher risk of COVID-19 hospitalisation or death after the autumn booster dose. Those with a larger household size (≥11 people within household vs 2 people; 1.56 (1.23–1.98)) and from more deprived areas (most deprived vs least deprived quintile; 1.35 (1.21–1.51)) had modestly higher risks. We also observed at least a two-fold increase in risk for those with various chronic neurological conditions, including Down's syndrome, immunodeficiency, chronic kidney disease, cancer, chronic respiratory disease, or cardiovascular disease.

**Interpretation:**

Males, older individuals, underweight individuals, those with an increasing number of comorbidities, from a larger household or more deprived areas, and those with specific underlying health conditions remained at increased risk of COVID-19 hospitalisation and death after the autumn 2022 vaccine booster dose. There is now a need to focus on these risk groups for investigating immunogenicity and efficacy of further booster doses or therapeutics.

**Funding:**

National Core Studies—Immunity, 10.13039/100014013UK Research and Innovation (Medical Research Council and Economic and Social Research Council), Health Data Research UK, the Scottish Government, and the 10.13039/501100000848University of Edinburgh.

**Research in context**

**Evidence before this study**

We searched PubMed for observational studies from January 1, 2020 until September 6, 2023, with no language restrictions, using the search terms “COVID-19”, “coronavirus”, “SARS-CoV-2”, “booster”, “vaccine”, and “risk factors”. Our searches identified 16 relevant papers reporting risk factors associated with COVID-19 vaccine breakthrough (i.e., COVID-19 hospitalisation or death) among people who had received a booster vaccine. Risk factors such as lower educational attainment and at least three weekly visits to indoor public places were found to be associated with SARS-CoV-2 infection post-first booster dose. Older age (particularly ≥80 year), being male, living in a care home or in a socioeconomically deprived area were associated with an increased risk of post-first booster COVID-19 related deaths. We also found four studies reporting the vaccine effectiveness of bivalent booster doses. However, we found no population-based evidence available regarding the factors linked to COVID-19 vaccine breakthrough after two booster doses (including bivalent vaccines).

**Added value of this study**

We found an increased risk of COVID-19 hospitalisation or death among individuals being male, older, underweight, those with a higher number of comorbidities, living in a household with a larger number of people, from a more deprived area and those with specific underlying health conditions—particularly chronic neurological conditions, Down's syndrome, immunodeficiency, chronic kidney disease, cancer, chronic respiratory disease, or cardiovascular disease—after receiving the autumn 2022 booster dose. In addition to confirming some of the previously identified risk factors for severe COVID-19 outcomes after the first booster dose, our large four UK nation analysis has identified additional risk factors.

**Implications of all the available evidence**

We provide national evidence identifying the high-risk population with severe COVID-19 outcomes post-autumn 2022 booster dose. This four UK nation-wide population-based study has found that after the autumn 2022 vaccine booster, males, older people, underweight individuals, those with more comorbidities, from larger household or more deprived areas and with certain underlying health conditions remain at highest risk of COVID-19 related hospitalisation and death. The UK's Joint Committee on Vaccination and Immunisation and international policymaking bodies with similar health systems should consider prioritising these individuals for the next round booster dose programme and COVID-19 therapeutics.

## Introduction

In preparation for the autumn and winter seasons of 2022/23, the UK's Joint Committee on Vaccination and Immunisation (JCVI) revised its COVID-19 vaccination programme to focus on individuals at increased risk of experiencing severe COVID-19 illness.^1^ Given the continued prevalence of the Omicron variant throughout the year, concerns had been raised about the possibility of vaccine-variant mismatch related to Omicron. In response, the UK Medicines and Healthcare products Regulatory Agency (MHRA) authorised the distribution of two updated bivalent (antigens for two variant strains presumed to be in high circulation) booster vaccines. Several studies from Israel, Singapore, South Korea and USA have reported the vaccine effectiveness of bivalent booster doses but none have reported on vaccine breakthrough (i.e., COVID-19 hospitalisation or death).^2^, ^3^, ^4^, ^5^ The Spikevax bivalent Original/Omicron vaccine, manufactured by Moderna, was approved on August 15, 2022, and the Comirnaty Original/Omicron BA.1 vaccine, manufactured by Pfizer-BioNTech, was approved on September 3, 2022. As part of any national vaccination campaign, it is important to understand which demographic and clinical characteristics of the population remain at risk of severe illness, despite receiving a vaccine. This information can be used to effectively identify individuals who may benefit from additional vaccine doses or alternative therapeutic options, such as monoclonal antibodies and antiviral medications, including when these should be given.

We recently reported on risk factors for severe COVID-19 outcomes after the first booster dose, including older age, being male, living in a care home, those with comorbidities and those with certain underlying health conditions such as individuals receiving immunosuppressants and those with chronic kidney disease.^6^ This built on our earlier analyses reporting risk profiles of COVID-19 hospitalisation or death in adults who received the first dose of vaccine and fully vaccinated (two doses) people in Scotland.^7^^,^^8^ These studies have been used to inform public health strategy and vaccination policy. Current UK vaccination policy is to offer future booster doses to individuals at high risk, and it is important to assess if there is a change in which groups of the population could benefit most. This is particularly important considering that the landscape of COVID-19 is changing constantly with the emergence of new strains, COVID-19 treatments being available and hybrid immunity. There is limited population level data analysing the uptake and impact of second dose boosters except a pre-print from our Office for National Statistics (ONS) analysis in England, which found that adults with certain health conditions (e.g., having learning disability or Down's syndrome, cancer of the blood and bone marrow) had a higher risk of COVID-19 death relative to other causes of death compared with individuals who did not have diagnoses of those comorbidities.^9^

We sought to use four UK nations’ data to identify risk factors for severe COVID-19 outcomes among those receiving an autumn 2022 booster dose. Specifically, we aimed to identify demographic and clinical characteristics associated with increased risks of COVID-19 hospitalisation or death. We also described the characteristics of those who received both an autumn 2022 booster vaccination and COVID-19 therapeutic treatment.

## Methods

### Study design

We conducted a prospective, multi-nation, observational cohort study of adults residing in England, Northern Ireland, Scotland, and Wales, who had received an autumn 2022 COVID-19 booster vaccination between September 1, 2022 and December 31, 2022. Statistical analyses were conducted on the risk of a severe COVID-19 outcome separately within each nation's secure Trusted Research Environment (TRE). Researchers accessed near-real-time, population-scale, anonymised, linked, individual-level health and sociodemographic data sources within each TRE. These sources included population demographics, residential history, COVID-19 vaccination history, General Practitioner (GP) diagnoses and prescribed medications, hospital admissions, and death records.^7^ Our analytical approach was to conduct separate, equivalent analyses within each nation, and then generate pooled estimates using fixed-effect meta-analyses for the UK.

### Data sources

Detailed data sources in each nation is available in Supplementary Material Data Sources section.

### Cohort

**Fig. 1:**
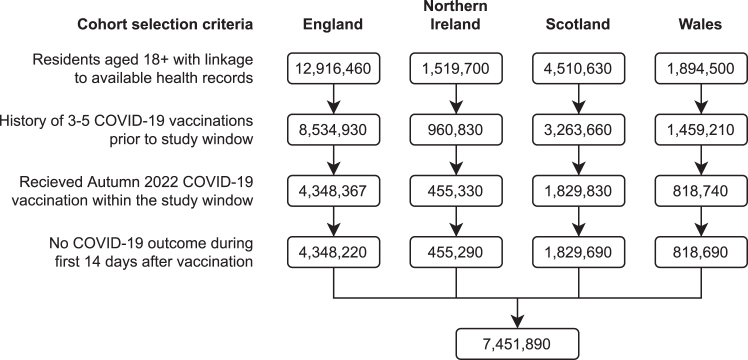
Cohort selection flow diagram.

For each country, we selected residents aged 18 years and older who received an autumn 2022 COVID-19 booster dose between September 1, 2022 and December 31, 2022 with at least two weeks of follow-up (Fig. 1). Booster doses were of either the Cominarty (Pfizer BioNTech BNT162b2) or Spikevax (Moderna mRNA-1273) vaccines.^1^ To be eligible for cohort selection, individuals needed to have basic demographic information available (such as week/month of birth and sex), as well as residential information (such as household ID and local area ID) and linked primary care records or hospital data. They also needed to have received three or more previous COVID-19 vaccinations, as per UK Health Security Agency (UKHSA) guidance.^10^ We placed no restrictions on the type of vaccines previously received. To allow time for a full immune response, we started the follow-up period 14 days after the booster dose was administered. We excluded individuals who experienced the outcome of interest within the first 14 days of being vaccinated.

### Outcome

Our primary outcome was the time until a severe COVID-19 event, defined as either an emergency hospital admission or death caused by COVID-19, whichever occurred first during the follow-up period. To ensure that the appropriate hospital admissions were accurately identified, we required the first episode of an emergency admission to have COVID-19 recorded as the lead International Classification of Diseases, Tenth Revision (ICD-10) diagnostic code. Similarly, for deaths, we required that an ICD-10 code for COVID-19 be recorded as the underlying cause of death. For Northern Ireland, Scotland, Wales, if a severe COVID-19 outcome did not occur, observations were censored at the end of the study window or when the individual moved out of the respective nation or died due to other causes. For England, observations were only censored at the end of the study window. Censoring due to other cause or movement affected less than 0.1% of individuals. Table S1 lists the ICD-10 codes we used to identify COVID-19 illness.

As a sensitivity analysis, we used broad definitions of COVID-19 hospitalisation and death. We allowed COVID-19 to be recorded in any diagnostic position for the first episode of an emergency hospital admission and as either the underlying or secondary cause of death.

### Booster vaccination

Our analysis focused on the autumn 2022 booster dose vaccination. Since immunological responses were not expected until 14 days after vaccination, individuals were defined as exposed starting at 14 days post-vaccination.^11^ We used two measures to capture vaccination: the type of vaccine administered and the time elapsed post-vaccination. We categorised vaccines as either ‘Cominarty’ or ‘Spikevax’, as records did not state whether the vaccines administered were of the original or adapted versions (although data from public health agencies indicate that the overwhelming majority were bivalent boosters). We measured time post-vaccination in two-week intervals up to 12 weeks. We chose these intervals to detect any potential vaccine waning.

### Covariates

Characteristics of interest were defined at baseline and included COVID-19 vaccination history, age, sex, ethnic group, body mass index (BMI), number of comorbidity risk groups, household size, socioeconomic deprivation (based on deprivation quintiles: quintile 1 refers to the most deprived and quintile 5 refers to the most affluent) and rural/urban classification of the residing area, and healthcare administrative area. We summarised COVID-19 vaccination history by counting the number of previous doses and flagging if the most recent dose was within the last 24 weeks before the start of the study. Age was grouped into four categories: 18–49, 50–64, 65–79, and ≥85 years. Ethnic group was grouped into five categories: White, Asian, Black, Mixed, Other, and an “Unknown” category for missing values.^12^ BMI was only available for England, Scotland and Wales and was defined as the most recently recorded value in the last five years. This meant values were only available for approximately half of the population in each nation. Thus, missing values were imputed once on the log scale using all study variables. Similar to the methods we used in our previous analyses,^6^^,^^13^ we imputed the missing BMI data using least squares regressions with all other independent variables included as predictors.

Comorbidity risk groups in England, Scotland and Wales were defined by the QCOVID risk prediction algorithm. The algorithm consists of 30 clinical conditions that have been shown to be associated with an increased risk of COVID-19, Table S3 lists the items used by each nation.^14^ This was however not available for Northern Ireland. For Northern Ireland, we therefore employed our previously developed approach of defining underlying clinical risks based on the number of different British National Formulary (BNF) chapters from which an individual had received repeat medications within the last 12 months. COVID-19 therapeutics, including monoclonal antibody and antiviral treatment, was only available in Scotland, thus these data were summarised narratively.

Each TRE made available linked residential IDs, allowing us to calculate household size and look up the residing Lower-layer Super Output Area (LSOA). Using the LSOA, the appropriate index of multiple deprivation quintile and urban/rural classification could be joined.^15^, ^16^, ^17^, ^18^, ^19^ Finally, the residing healthcare administrative area was used to control for potential variation regarding the background prevalence of COVID-19 and the availability of vaccinations. These were NHS regions in England, local councils in Northern Ireland and health boards in Scotland and Wales.

### Statistical analysis

Our analytical approach consisted of two stages: an overall adjusted analysis of the sociodemographic characteristics and the outcome of interest, and then a specific analysis looking at the individual clinical conditions separately. Across both stages, we fitted Cox proportional hazard models on the time-censored severe outcome. Follow-up time was defined as days from September 1, 2022, so that any temporal changes in the prevalence of COVID-19 would be automatically controlled for. Time post-vaccination was then incorporated as a categorical time-varying covariate, whilst baseline characteristics were included as main effects.

For our overall analysis, all summary characteristics were included (i.e., age, sex, BMI, household size, deprivation, urban/rural areas and comorbidities), and we first fitted a single model for all those who had received either Cominarty or Spikevax, stratifying the baseline hazard by administrative area and by type of vaccine. The proportionality assumption was checked by testing and visually inspecting the Schoenfeld residuals. We then fitted subset models for each vaccine separately to generate vaccine-specific effects. For our analysis of specific clinical conditions, we fitted a separate model for each condition with the same approach as our overall analysis but only adjusted vaccination, age, sex, household size, deprivation, rural/urban i.e., no adjustment for other underlying clinical conditions.

Due to data governance arrangements with each TRE, analyses were performed separately for each nation and then combined in Public Health Scotland's TRE. Descriptive summaries were simply pooled by summing and recalculating percentages and rates per 1000 person-years. Adjusted hazard ratios (aHR) with corresponding 95% confidence intervals (CIs) were meta-analysed using a fixed-effect, generic inverse variance approach. Cohran's Q-test was used to assess the extent of heterogeneity. Finally, our sensitivity analysis consisted of repeating our approach using a broad definition of the outcome.

Statistical analyses were conducted by UA for England, LP for Northern Ireland, FA for Scotland, and SB for Wales and the corresponding meta-analyses in each nation's TRE. Results were independently checked by MJ, CR, UA, HA, DTB, and TS. All analyses were implemented in R (version 4.1.2 in England, 4.1.0 in Northern Ireland, version 3.6.3 in Scotland, and version 4.1.2 in Wales).

### Use of reporting guideline

We followed the Reporting of Studies Conducted using Observational Routinely-collected Data (RECORD) and the Strengthening the Reporting of Observational studies in Epidemiology (STROBE) checklists^20^^,^^21^ to guide transparent reporting of this cohort study (Table S2).

### Patient and public involvement

We have patient and public involvement engagement throughout the project. The details are available in Supplementary Materials Tables S4 and S5.

### Ethics and permissions

In England, ethical approval was granted by the Health Research Authority London Central Research Ethics Committee (reference number REC reference 21/HRA/2786; integrated research application system number 301740). In Northern Ireland, study approval was granted by the Honest Broker Service (HBS) Governance Board (project number 064; the HBS process does not require separate National Research Ethics Service governance approval). In Scotland, ethical approval was granted by the National Research Ethics Service Committee (Southeast Scotland 02; reference number 12/SS/0201), and the approval for data linkage was granted by the Public Benefit and Privacy Panel for Health and Social Care (reference number 1920-0279). In Wales, research conducted within the Secure Anonymised Information Linkage (SAIL) Databank was done with the permission and approval of the independent Information Governance Review Panel (project number 0911). Individual written patient consent was not required for this study.

### Role of the funding source

The funder of the study had no role in study design, data collection, data analysis, data interpretation, or the writing of the report.

## Results

Between September 1, 2022 and December 30, 2022, a total of 7,451,890 individuals aged 18 and older received a COVID-19 vaccination as part of the autumn 2022 booster programme in England, Northern Ireland, Scotland, and Wales (Table 1, Tables S6, S7 and S8). Those vaccinated were older than the general population (87.7% aged 50 years or older), more likely to be female (54.2%), and overweight (41.7% BMI 25.0–29.9). They received a dose of either the Comirnaty vaccine (55.7%) or the Spikevax vaccine (44.3%). Of those vaccinated, 3500 experienced a severe COVID-19 outcome, at a rate of 2.9 severe outcomes per 1000 person-years.

**Table 1:** Combined sample characteristics and rates of severe COVID-19 outcomes for individuals who received an autumn 2022 booster vaccination, across England (n = 4,348,220), Northern Ireland (n = 455,290), Scotland (n = 1,829,690), and Wales (n = 818,690).

	Overall		Comirnaty		Spikevax^a^	
	n (%)	Outcome (rate)	n (%)	Outcome (rate)	n (%)	Outcome (rate)
**Total**	7,451,890 (100.0%)	3500 (2.9)	4,149,040 (100.0%)	1370 (2.5)	3,292,460 (100.0%)	2120 (3.2)
**Autumn 2022 vaccination**						
Comirnaty	4,149,040 (55.7%)	1370 (2.5)	–	–	–	–
Spikevax	3,302,840 (44.3%)	2120 (3.2)	–	–	–	–
**Time post Autumn 2022 vaccination**						
2–3 weeks	7,410,530 (99.4%)	2190 (18.7)	4,107,830 (99.0%)	850 (13.7)	3,292,310 (100.0%)	1340 (24.5)
4–5 weeks	7,161,680 (96.1%)	2030 (18.7)	3,907,880 (94.2%)	770 (13.6)	3,243,410 (98.5%)	1260 (24.4)
6–7 weeks	6,600,560 (88.6%)	1840 (19.6)	3,460,230 (83.4%)	680 (14.5)	3,129,970 (95.1%)	1170 (25.1)
8–9 weeks	5,651,400 (75.8%)	1650 (21.8)	2,659,700 (64.1%)	540 (15.7)	2,981,670 (90.6%)	1110 (27.2)
10–11 weeks	4,318,160 (57.9%)	1290 (24.8)	1,589,780 (38.3%)	290 (15.3)	2,719,600 (82.6%)	1000 (30.4)
≥12 weeks	2,578,310 (34.6%)	960 (25.8)	478,380 (11.5%)	80 (16.2)	2,094,940 (63.6%)	880 (27.4)
**Number of previous COVID-19 vaccinations**						
3	5,413,160 (72.6%)	920 (1.1)	3,319,830 (80.0%)	480 (1.1)	2,085,980 (63.4%)	440 (1.1)
4	1,894,140 (25.4%)	2410 (6.6)	762,570 (18.4%)	830 (7.1)	1,128,630 (34.3%)	1580 (6.4)
5	144,580 (1.9%)	160 (6.2)	66,650 (1.6%)	60 (6.6)	77,850 (2.4%)	100 (6.0)
**Time since previous COVID-19 vaccination**						
<24 weeks	1,168,630 (15.7%)	1670 (6.9)	388,530 (9.4%)	460 (7.2)	777,720 (23.6%)	1210 (6.8)
≥24 weeks	6,283,260 (84.3%)	1830 (1.9)	3,760,500 (90.6%)	920 (1.9)	2,514,740 (76.4%)	910 (1.9)
**Sex**						
Female	4,040,810 (54.2%)	1710 (2.6)	2,213,360 (53.3%)	640 (2.2)	1,822,120 (55.3%)	1050 (2.9)
Male	3,411,070 (45.8%)	1790 (3.3)	1,935,680 (46.7%)	730 (2.9)	1,470,340 (44.7%)	1070 (3.7)
**Age**						
18–49 years	920,100 (12.3%)	60 (0.5)	537,670 (13.0%)	20 (0.3)	381,620 (11.6%)	40 (0.6)
50–64 years	2,767,200 (37.1%)	280 (0.8)	1,907,760 (46.0%)	130 (0.6)	856,560 (26.0%)	130 (0.9)
65–79 years	2,759,880 (37.0%)	1150 (2.3)	1,320,880 (31.8%)	500 (2.4)	1,434,110 (43.6%)	650 (2.1)
≥80 years	1,004,710 (13.5%)	2010 (10.2)	382,740 (9.2%)	720 (12.2)	620,160 (18.8%)	1290 (9.3)
**Ethnicity**^a^						
White	5,923,840 (84.7%)	2960 (3.0)	3,136,170 (84.7%)	1030 (2.5)	2,787,670 (84.7%)	1930 (3.4)
Asian	204,880 (2.9%)	60 (1.8)	104,870 (2.8%)	10 (0.8)	100,010 (3.0%)	40 (2.0)
Black	56,660 (0.8%)	10 (1.2)	31,960 (0.9%)	10 (2.7)	24,690 (0.7%)	10 (2.0)
Mixed	37,890 (0.5%)	10 (1.7)	20,820 (0.6%)	0 (0.0)	17,070 (0.5%)	10 (3.0)
Other	30,280 (0.4%)	0 (0.0)	16,490 (0.4%)	0 (0.0)	13,780 (0.4%)	0 (0.0)
Unknown	743,060 (10.6%)	220 (2.0)	393,830 (10.6%)	80 (1.6)	349,240 (10.6%)	130 (2.1)
**BMI**^a^						
<18.5	107,510 (1.5%)	180 (10.0)	54,770 (1.5%)	50 (7.1)	52,740 (1.6%)	140 (12.8)
18.5–24.9	1,845,890 (26.4%)	1110 (3.6)	989,730 (26.7%)	380 (2.9)	856,160 (26.0%)	730 (4.1)
25.0–29.9	2,918,540 (41.7%)	1150 (2.4)	1,494,830 (40.4%)	410 (2.1)	1,423,710 (43.2%)	740 (2.7)
30.0–39.9	1,795,540 (25.7%)	710 (2.4)	977,600 (26.4%)	260 (2.0)	817,930 (24.8%)	450 (2.8)
≥40.0	329,120 (4.7%)	110 (2.2)	187,210 (5.1%)	40 (1.7)	141,900 (4.3%)	60 (2.2)
**Number of QCovid risk groups**^a^						
0	2,840,040 (40.6%)	330 (0.8)	1,592,440 (43.0%)	130 (0.7)	1,247,610 (37.9%)	200 (0.8)
1	1,982,220 (28.3%)	560 (1.7)	1,048,920 (28.3%)	220 (1.6)	933,300 (28.3%)	340 (1.8)
2	1,037,770 (14.8%)	650 (3.7)	523,060 (14.1%)	220 (3.1)	514,720 (15.6%)	440 (4.1)
3	569,720 (8.1%)	580 (5.8)	277,610 (7.5%)	200 (5.2)	292,110 (8.9%)	390 (6.3)
4	299,890 (4.3%)	450 (8.4)	141,360 (3.8%)	160 (8.1)	158,530 (4.8%)	290 (8.5)
≥5	266,960 (3.8%)	690 (14.2)	120,780 (3.3%)	220 (13.1)	146,180 (4.4%)	470 (14.8)
**Number of BNF risk groups**^c^						
0	95,590 (21.0%)	0 (0.0)	93,930 (21.1%)	0 (0.0)	–	–
1	86,530 (19.0%)	20 (1.5)	84,580 (19.0%)	10 (0.8)	–	–
2	83,360 (18.3%)	20 (1.6)	81,400 (18.3%)	20 (1.6)	–	–
3	68,430 (15.0%)	40 (3.7)	66,770 (15.0%)	40 (3.9)	–	–
4	50,490 (11.1%)	40 (5.0)	49,180 (11.1%)	40 (5.2)	–	–
5	33,680 (7.4%)	40 (7.5)	32,800 (7.4%)	40 (7.8)	–	–
≥6	37,200 (8.2%)	70 (11.9)	36,240 (8.1%)	70 (12.3)	–	–
**Household size**^b^						
1 person	865,620 (27.9%)	570 (4.1)	411,360 (24.9%)	220 (3.8)	452,380 (31.4%)	340 (4.1)
2 people	1,191,330 (38.4%)	580 (3.0)	635,440 (38.5%)	240 (2.7)	552,180 (38.3%)	340 (3.3)
3–5 people	950,270 (30.6%)	250 (1.9)	551,340 (33.4%)	120 (1.7)	394,780 (27.4%)	120 (1.8)
6–10 people	68,950 (2.2%)	20 (2.0)	43,520 (2.6%)	0 (0.0)	24,890 (1.7%)	10 (2.3)
≥11 people	27,490 (0.9%)	90 (15.9)	9150 (0.6%)	10 (7.5)	18,270 (1.3%)	70 (16.1)
**Socioeconomic deprivation quintile**						
5th (Least)	1,942,060 (26.1%)	820 (2.6)	1,048,210 (25.3%)	330 (2.3)	892,060 (27.1%)	490 (2.8)
4th	1,721,810 (23.1%)	740 (2.7)	990,110 (23.9%)	300 (2.3)	729,800 (22.2%)	440 (3.0)
3rd	1,526,680 (20.5%)	660 (2.7)	883,290 (21.3%)	280 (2.4)	641,200 (19.5%)	380 (2.9)
2nd	1,288,760 (17.3%)	680 (3.3)	714,740 (17.2%)	240 (2.5)	571,780 (17.4%)	430 (3.8)
1st (Most)	972,550 (13.1%)	620 (4.0)	512,690 (12.4%)	240 (3.6)	457,620 (13.9%)	380 (4.3)
**Rural/urban area classification**						
Urban	5,694,210 (76.4%)	2840 (3.1)	3,076,630 (74.2%)	1080 (2.7)	2,611,270 (79.3%)	1760 (3.4)
Rural	1,757,670 (23.6%)	660 (2.3)	1,072,400 (25.8%)	290 (2.0)	681,180 (20.7%)	360 (2.6)

Rates are per 1000 person-years. Counts between 1 and 9 have been suppressed, all other counts rounded to nearest 10.

a England, Scotland and Wales only.

b Scotland, Wales, Northern Ireland only.

c Northern Ireland only.

**Fig. 2:**
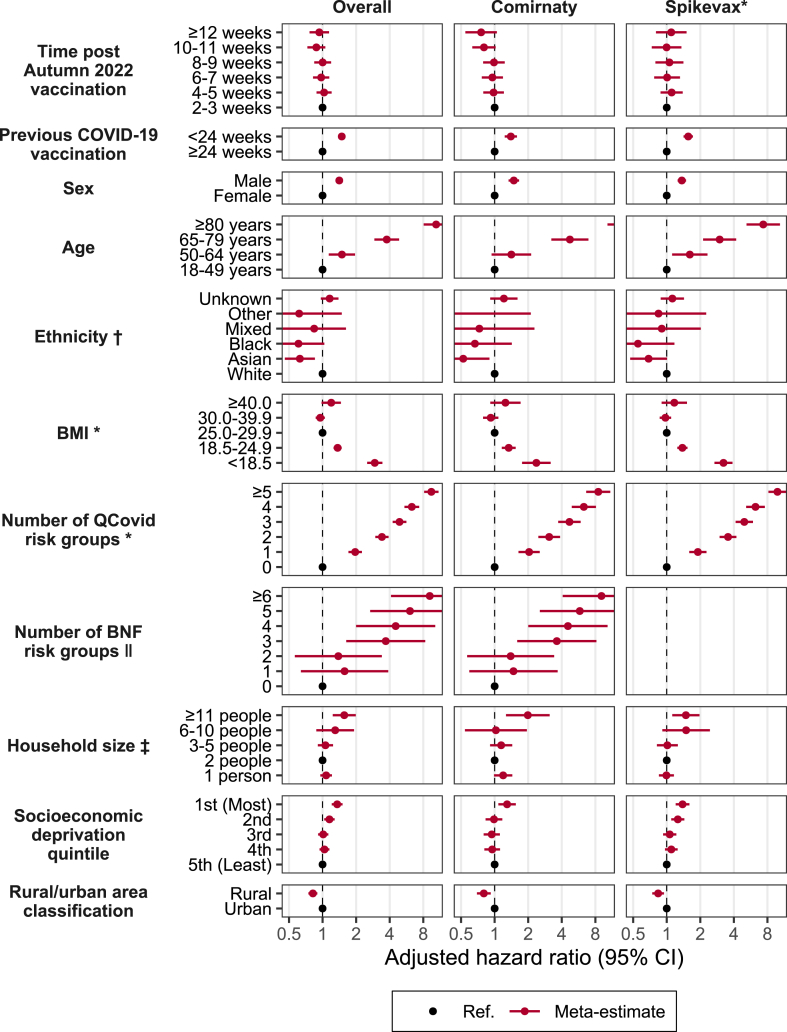
Meta, adjusted hazard ratios with 95% confidence intervals for vaccination, socio-demographics and clinical factors associated with severe COVID-19 outcomes, across England (n = 4,348,220), Northern Ireland (n = 455,290), Scotland (n = 1,829,690), and Wales (n = 818,690). ∗ England, Scotland and Wales only. † England only. ‡ Scotland, Wales, Northern Ireland only. ‖ Northern Ireland only.

Our main survival analysis (Fig. 2) indicated that neither Comirnaty or Spikevax showed evidence of waning up to 12 weeks post-vaccination, and associated risk factors were comparable across both vaccines. All two-week periods post-vaccination were found similar to the initial 2–3 week reference period. Males, older individuals, underweight individuals, or those with higher numbers of comorbidities were all at a greater risk of a severe COVID-19 outcome post the autumn 2022 booster dose, compared to their counterparts. Furthermore, individuals living with a larger number of people or in areas of greater deprivation were at a modest increased risk of experiencing a severe COVID-19 outcome.

Those who received a vaccination within 24 weeks prior to their autumn booster were aHR (95% CI) 1.49 (1.38–1.60) times more likely to experience a severe outcome compared to those who had their last vaccination 24 or more weeks prior (Fig. 2, Table S8). Males were 1.41 (1.32–1.51) times more likely than females to experience the severe outcome. In comparison to those aged 18–49, those aged 80 years or older were 10.43 (8.06–13.50) times more likely to experience the outcome. People in the Asian ethnic group were found to be 0.63 (0.46–0.85) times as likely to experience the outcome compared to those in the White ethnic group, whilst other ethnic minority groups differed non-significantly. For BMI, we found that those classified as underweight (BMI <18.5) were at the greatest risk of having a severe outcome, 2.94 (2.51–3.44) times more likely than those classified as overweight (BMI 25.0–29.9). Additionally, those classified as a healthy weight (BMI 18.5–24.9) were 1.36 (1.25–1.48) times more likely to experience a severe outcome than those classified as overweight.

Regarding number of comorbidities, we found that increasing number of QCOVID risk groups was positively associated with the risk of a severe outcome. Those with one underlying clinical condition were 1.96 (1.71–2.25) times more likely than those with no conditions, and those with 5 or more conditions were 9.45 (8.15–10.96) times more likely. For Northern Ireland, we used BNF chapters to measure the clinical conditions and observed a similar pattern to other nations using number of clinical conditions (with wide confidence intervals due to smaller sample size).

In terms of household size, we found a significant increase in risk for those living in a household of 11 or more people compared to those living in 2-person households. They were 1.56 (1.23–1.98) times more likely to experience a severe outcome, while all other household sizes had comparable risks. Regarding the residing area, compared to those living in the 5th quintile of socioeconomic deprivation (the least deprived areas), those living in the 4th and 3rd quintiles had similar risks. However, those living in the 2nd quintile and 1st quintile (the most deprived areas) were 1.15 (1.04–1.28) and 1.35 (1.21–1.51) times more likely to experience a severe outcome, respectively. We found those living in rural areas to be at 0.82 (0.75–0.90) times the risk of those living in urban areas.

For Wales, Northern Ireland, and Scotland, global non-proportionality hazard ratio tests did not show any substantial departures (Table S9), while for England a significant departure was observed. This is largely associated with BMI and age group. Inspecting the residual plots (Figure S1a–d) showed that the non-proportionality for BMI was minor. For age group the non-proportionality was more severe with definite curvature particularly at the longer follow up times. The implication is that the HR for age group in England should be interpreted as a weighted average of the time-varying hazard ratios.

### Comorbidities

**Fig. 3:**
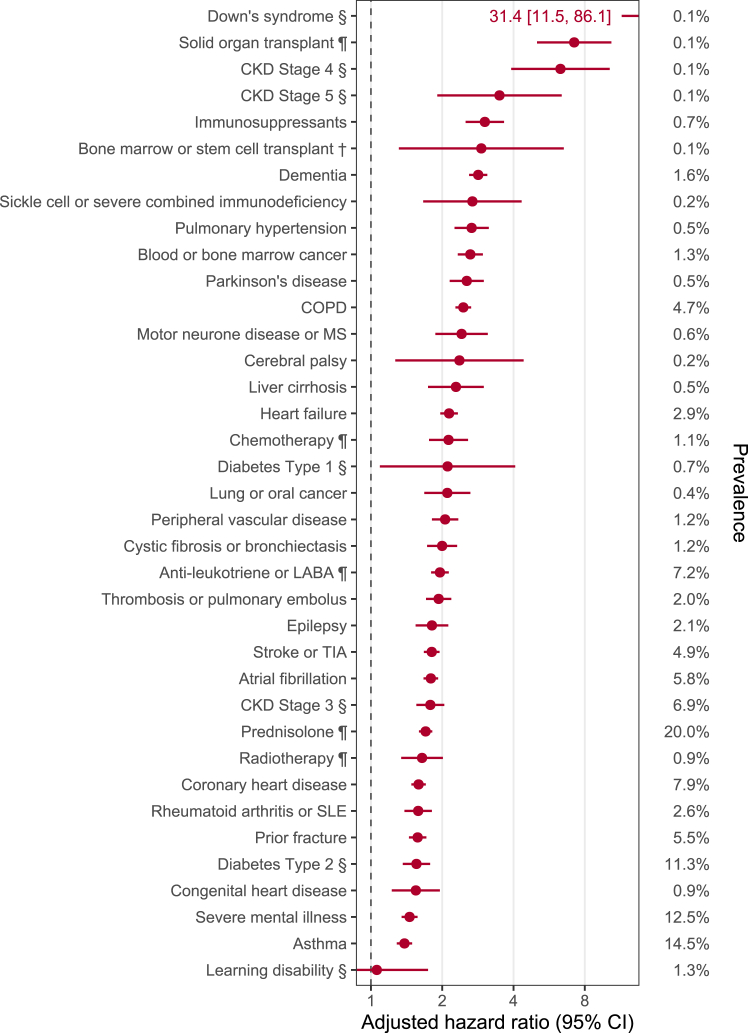
Meta, adjusted hazard ratios with 95% confidence intervals for individual QCovid clinical conditions associated with severe COVID-19 outcomes, across England (n = 4,348,220), Scotland (n = 1,829,690), and Wales (n = 818,690), only. Point size is mapped to the prevalence of the condition among the combined cohort. † England only. § Scotland and Wales only. ¶ England and Wales only.

The pooled counts and rates of severe COVID-19 outcome for individual QCOVID clinical conditions are shown in Table 2 (Table S10 for nation counts). We found that the majority of comorbidities were associated with an increased risk of severe COVID-19 outcomes (Fig. 3 and Table S11). However, due to the low prevalence of certain health conditions and the overall rate of events among those vaccinated, some estimates had a large degree of uncertainty. Nevertheless, we were able to identify at least a two-fold increase in risk for those with various chronic neurological conditions, immunodeficiency, chronic kidney disease, cancer, chronic respiratory disease or cardiovascular disease.

**Table 2:** Combined counts and rates for individual QCovid clinical conditions, across England (n = 4,348,220), Scotland (n = 1,829,690), and Wales (n = 818,690), only.

	n	%	Outcome	Rate
**Total**	6,996,600	100.0%	3260	2.9
Anti-leukotriene or LABA^c^	370,350	7.2%	360	5.7
Asthma	1,013,530	14.5%	530	3.3
Atrial fibrillation	404,530	5.8%	690	9.2
Blood or bone marrow cancer	89,580	1.3%	170	10.7
Bone marrow or stem cell transplant^a^	3400	0.1%	–	–
Cerebral palsy	12,390	0.2%	–	–
Chemotherapy^c^	55,260	1.1%	60	6.2
CKD Stage 3^b^	183,980	6.9%	320	9.3
CKD Stage 4^b^	2650	0.1%	20	39.2
CKD Stage 5^b^	3830	0.1%	10	13.4
Congenital heart disease	62,290	0.9%	50	4.8
COPD	327,080	4.7%	610	10.5
Coronary heart disease	554,740	7.9%	720	7.1
Cystic fibrosis or bronchiectasis	81,170	1.2%	130	8.8
Dementia	112,930	1.6%	490	22.2
Diabetes Type 1^b^	18,870	0.7%	–	–
Diabetes Type 2^b^	300,520	11.3%	290	5.7
Down's syndrome^b^	1350	0.1%	–	–
Epilepsy	149,860	2.1%	110	4.7
Heart failure	202,690	2.9%	420	11.3
Immunosuppressants	51,300	0.7%	50	5.8
Learning disability^b^	35,200	1.3%	–	–
Liver cirrhosis	32,690	0.5%	30	5.4
Lung or oral cancer	31,250	0.4%	50	9.0
Motor neurone disease or MS	45,110	0.6%	40	5.4
Parkinson's disease	38,190	0.5%	100	14.2
Peripheral vascular disease	84,960	1.2%	160	10.4
Prednisolone^c^	1,032,610	20.0%	820	4.6
Prior fracture	384,870	5.5%	440	6.6
Pulmonary hypertension	36,540	0.5%	90	13.5
Radiotherapy^c^	46,320	0.9%	50	6.0
Rheumatoid arthritis or SLE	179,390	2.6%	140	4.6
Severe mental illness	875,380	12.5%	480	3.4
Sickle cell or severe combined immunodeficiency	11,090	0.2%	–	–
Solid organ transplant^c^	7690	0.1%	10	7.7
Stroke or TIA	340,050	4.9%	560	9.0
Thrombosis or pulmonary embolus	137,500	2.0%	180	7.5

Rates are per 1000 person-years. Counts between 1 and 9 have been suppressed, all other counts rounded to nearest 10.

a England only.

b Scotland and Wales only.

c England and Wales only.

### COVID-19 therapeutic treatment

We were only able to access data on COVID-19 therapeutic treatment for those in the Scottish cohort. Given our study approach, this information was difficult to analyse due to how therapeutic treatment was made available. To be eligible for treatment, someone would have to test positive for COVID-19, have a symptomatic response, and be considered at highest risk of getting seriously ill. We provide a descriptive profile in Table S12 of those who had treatment, stratifying by whether this was before or after their autumn booster vaccination. The characteristics were consistent with the eligibility criteria. Of those vaccinated in Scotland (n = 1,829,690), 11,150 (0.6%) received therapeutic treatment before their autumn booster, and 690 received treatment within the study window after the booster.

### Sensitivity analysis

Using a broader definition of COVID-19 hospitalisation or death over the same study window, we found that the number of events was approximately double compared to using our strict definition, 6910, at a rate of 5.7 outcomes per 1000 person-years (Table S13). However, the estimated aHRs for the severe COVID-19 outcome under the broad definition were overall similar to our results from the strict definition (Table S14 and Figure S2). Exceptions were the general decrease in risk associated with older age groups, though we expect this is due to those aged 18–49 years old having disproportionately more outcomes based on broad definitions. The other main exception was that those with a BMI over 40.0 were of slightly greater risk of the broad outcome compared to the strict outcome.

## Discussion

We provide UK-wide evidence identifying individuals at increased risk of COVID-19 hospitalisation or death after having received the autumn 2022 booster dose. Our analysis identified risk factors that have been previously reported (e.g., strong positive associations with increasing age), but we also showed a range of additional risk groups, including Down's syndrome, large household size and cardiovascular disease. These findings have been shared with JCVI to inform future vaccination policy.

Our study has several strengths. This was a national study representing four UK nations identifying, characterising and quantifying the risk of severe COVID-19 outcomes in those who have received an autumn 2022 booster dose. The analysed population is representative for the UK's population (∼99% coverage for Northern Ireland and Scotland, 86% for Wales and 33% for England). Whilst selection bias is potentially a concern in England, previous work has shown that the Royal College of General Practitioners (RCGP) Research and Surveillance Centre (RSC) has a nationally representative population.^22^ Our primary outcome—COVID-19 hospitalisation or death was based on the strict definitions where only hospitalisation or death due to COVID-19 were included therefore the demographic and clinical factors identified in our study were very likely contributing to COVID-19 severe outcomes in a causal relationship. We also applied a broad definition for COVID-19 hospitalisation or death where hospitalisation or death related to COVID-19 (as the secondary or underlying factor) were also included to explore the overall hospital pressure associated with severe COVID-19 outcome. Despite direct comparisons not technically feasible, due to differences in who was eligible to be vaccinated at the time, the results under our analysis using broad definitions offered similar insights (similar hazard ratios) to our previous analyses–COVID-19 hospitalisation or death of the 2021 autumn booster,^6^^,^^23^ as well as our analyses of the post-first vaccine dose^7^ and in fully vaccinated people (two doses).^8^

Our study has several limitations. We only included a four-month study period in our study which did not capture all COVID-19 hospitalisation or death post-autumn 2022 booster dose. There may also have been different healthcare seeking behaviours and lower threshold for hospital admission (influenced by physician and hospital factors) in adults with pre-existing health conditions, which may have resulted in a higher risk of hospital admissions with COVID-19 although we restricted our main analysis to those hospitalisations due to COVID-19 instead of with COVID-19 (where COVID-19 was the secondary or underlying factor for hospital admission). We did not restrict the England hospitalisation information to emergency hospitalisation. This means there is a risk that some elective activity is being captured. However, this was more likely to happen under the broad definition and is unlikely to have impacted on our main findings. Censoring due to death from other causes or transfer out was not done in England. This will have minimal impact as less than 0.1% of individuals in the other 3 countries were censored. Our analysis was not able to include some potentially important confounders (such as tobacco exposure) due to the lack of reliable recording of these variables within electronic health records, with the consequence that residual confounding remains a possibility. Prior SARS-CoV-2 infection was not taken into consideration which may have affected the absolute rates presented in Table 1, where the raw numbers were pooled and a rate was calculated. However, the rates were unadjusted which did not address age structure. We did not stratify the analysis by the number of previous vaccine doses, different types of vaccines, mixing types of vaccines, other classification of age groups or the combination of age classification and age as these were not within the remit of our study or we did not have sufficient power. These are however important questions for future analyses. Pre-existing health conditions were defined by QCOVID risk prediction algorithm in England, Scotland, and Wales while by BNF chapters in Northern Ireland, which did not allow to combine data across four UK nations for this covariate. We used ICD-10 codes to define the outcome, which might introduce some random misclassification bias. Some potential confounders could be correlated to health conditions, for example, heart insufficiency and high BMI or cancer disease and underweight, which might possibly overestimate the impact of a single health condition. Due to information governance being a devolved issue in the UK, we could not carry out a single UK-wide analysis at the individual-level. Therefore, our approach was to implement separate cohort studies in each nation and combine the results assuming a single effect size for the UK. We therefore chose to undertake a fixed-effect meta-analysis. Lastly, the findings on vaccine-specific effects were exploratory and the reported test statistics were not adjusted for multiple comparisons. Missing data were handled using single imputation, which is unlikely to capture fully the uncertainty in relation to the prediction of missing values and could be less powerful than multiple imputations.

Similar findings have been reported on vaccine breakthrough from previous articles. One study from the ONS reported older adults (particularly those aged at least 80 years), males, living in a care home or in a socioeconomically deprived area were associated with an increased risk of COVID-19 death post-first booster dose.^23^ Based on the data from 465 facilities in a large US health care database, adults aged at least 65 years, were immunosuppressed, or had other underlying conditions were at increased risk of COVID-19 related severe outcome (hospitalisation or death) after completing a full primary COVID-19 vaccination series (receipt of two doses of an mRNA vaccine or a single dose of JNJ-78436735).^24^ All persons with severe outcomes had at least one of these risk factors, and 77.8% of those who died had four or more risk factors.^24^ Different risk factors for vaccine breakthrough have been reported in other studies. A population-based cohort study in UK found that lower educational attainment and at least three weekly visits to indoor public places were associated with a higher risk of SARS-CoV-2 infection in both post-primary and post-first booster vaccine.^25^ A prospective cohort study in Belgium has reported that vaccination with adenoviral-vector vaccines might be associated with breakthrough infections (laboratory confirmed SARS-CoV-2 infections) after full primary vaccination.^26^ In addition, higher rates of face mask use was reported associated with a lower risk of a positive SARS-CoV-2 test after full primary vaccination.^27^ Many of these factors associated with the failure of booster vaccines to protect from severe COVID-19 in some people might be related to relative degrees of immuno-compromise or immuno-senescence, such that individuals do not mount a sufficient antibody response to prevent severe disease. Added to this underlying state, will be the extent of social and domestic exposure to virus and the prevalence of variants with vaccine-escape in circulation.

In our study, most individuals who had severe COVID-19 outcome were being male, older, underweight, those with higher number of comorbidities, living in a household with a larger number of people, from a more deprived area and those with specific underlying health conditions. These results are similar to the risk profile for COVID-19 hospitalisation or death in vaccinated individuals who have received one, two or three (first booster) doses of vaccine.^6^, ^7^, ^8^ Risk of severe COVID-19 outcome is therefore not completely eliminated when fully vaccinated or offered booster doses; the results of this study suggest the importance of continued caution and non-pharmaceutical interventions as well as COVID-19 therapeutics, in particular for those at high risk.

### Conclusion

In summary, this UK-wide analysis has detailed the characterisation of individuals with an increased risk of COVID-19 hospitalisation or death post-autumn booster dose. The findings have been shared with JCVI to inform future plans for the roll-out of further COVID-19 booster programmes and COVID-19 therapeutics.

## Contributors

AS, CR, and TS conceived this study. AS, CR, TS and SB commented on the paper, oversaw the analysis, and edited the final manuscript. TS and SB led the writing of the paper. SdL and MJ conceived how Research and Surveillance Centre data could support this study and are the guarantors of these data; RG and RB extracted and prepared the data in England and UA conducted the analyses. LP and DTB were responsible for data cleaning, and LP contributed to the analysis in Northern Ireland. FA accessed and verified the underlying data and is responsible for data cleaning and analysis in Scotland. SB accessed and verified the underlying data and is responsible for data cleaning and analysis in Wales. SB led the meta-analysis with support from UA, FA and LP. CR oversaw all the analyses. All authors contributed to the study design. All authors contributed to drafting the paper and revised the manuscript for important intellectual content. All authors had final responsibility for the decision to submit for publication.

## Data sharing statement

All code used in this study is publicly available online. The data used in this study are sensitive due to individual level data and will not be made publicly available.

## Data availability

A data dictionary covering the data sources used in this study can be found at https://github.com/EAVE-II/EAVE-II-data-dictionary. All code developed for this analysis is available at https://github.com/HDRUK/uk-vaccine-breakthrough-autumn-2022https://github.com/EAVE-II. We will also deposit the meta-data information in Health Data Research Innovation Gateway on publication. The Statistical Analysis Plan for this study is available in the Supplementary Materials Protocol Section.

## Declaration of interests

AS and CR are members of the Scottish Government's CMO COVID-19 Advisory Group. AS and CR are members of NERVTAG's risk stratification subgroup. CR is a member of SPI-M. AS is a member of AstraZeneca's Thrombotic Thrombocytopenic Advisory Group and the Scottish Government's Standing Committee on Pandemics. All roles are unremunerated. RAL is a member of the Welsh Government COVID-19 Technical Advisory Group. All other co-authors report no conflict of interests. SdeL is Director of the RSC, through his university he has received vaccine related research funding from AstraZeneca, GSK, Sanofi, Seqirus, MSD and Takeda, and been member of advisory boards for AstraZeneca, GSK, Sanofi and Seqirus.
