# Supplementary material for: Risk of severe COVID-19 outcomes after autumn 2022 COVID-19 booster vaccinations: a pooled analysis of national prospective cohort studies involving 7.4 million adults in England, Northern Ireland, Scotland and Wales
Source: Lancet Reg Health Eur. 2023 Dec 12;37:100816. doi: 10.1016/j.lanepe.2023.100816 (PMC10757260; doi:10.1016/j.lanepe.2023.100816)
Supplement: Supplementary Tables and Figures [file mmc1.docx]

**Risk of severe COVID-19 outcomes after autumn 2022 COVID-19 booster vaccinations: a pooled analysis of national prospective cohort studies on 7.4 million adults in England, Northern Ireland, Scotland and Wales**

Supplementary material

Contents

[Reporting and Methodology 3](#_Toc145528359)

[**Table S1:** ICD-10 codes for COVID-19 illness 3](#_Toc145528360)

[**Table S2:** Reporting STROBE and RECORD checklists. 4](#_Toc145528361)

[**Table S3:** QCOVID risk groups and codes. 9](#_Toc145528362)

[Data sources 10](#_Toc145528363)

[Protocol 10](#_Toc145528364)

[**Research aims** 10](#_Toc145528365)

[**Cohort and sample design** 10](#_Toc145528366)

[**Outcome** 11](#_Toc145528367)

[**Exposure** 11](#_Toc145528368)

[**Covariates** 12](#_Toc145528369)

[**Exploratory analysis** 12](#_Toc145528370)

[**Describe cohort characteristics** 13](#_Toc145528371)

[**Main analysis of severe COVID-19 outcomes post autumn vaccination** 13](#_Toc145528372)

[**Specific clinical indicator analysis** 13](#_Toc145528373)

[Patient and public involvement with this study 14](#_Toc145528374)

[**Table S4**: GRIPP2 reporting checklist (short form) 14](#_Toc145528375)

[**GRIPP2 Reporting Guidelines: Short Form** 14](#_Toc145528376)

[**Table S5:** PPI deliverables in line with the research cycle. 15](#_Toc145528377)

[Underlying nation results 18](#_Toc145528378)

[**Table S6:** Number of people entering the study within each nation at 2-week intervals, along with number having the event. 18](#_Toc145528379)

[**Table S7:** Nation sample characteristics and rates of severe COVID-19 outcomes for individuals who received an Autumn 2022 booster vaccination. Rates are number of events per 1,000 person-years. Counts between 1 and 9 have been suppressed, all other counts rounded to nearest 10. 19](#_Toc145528380)

[**Table S8:** Nation and meta, adjusted hazard ratios with 95% confidence intervals, for vaccination, socio-demographics and clinical factors associated with severe COVID-19 outcomes. 29](#_Toc145528381)

[**Table S9:** Test results for the proportional hazards assumption for the main overall Cox regression models. 36](#_Toc145528382)

[**Figure S1a:** Smoothed trend of the Schoenfeld residuals for each variable in the overall main Cox regression model for England. 37](#_Toc145528383)

[**Figure S1b:** Smoothed trend of the Schoenfeld residuals for each variable in the overall main Cox regression model for Northern Ireland. 38](#_Toc145528384)

[**Figure S1c:** Smoothed trend of the Schoenfeld residuals for each variable in the overall main Cox regression model for Scotland. 39](#_Toc145528385)

[**Figure S1d:** Smoothed trend of the Schoenfeld residuals for each variable in the overall main Cox regression model for Wales. 40](#_Toc145528386)

[**Table S10:** Nation and pooled counts and rates of severe COVID-19 outcome for individual Qcovid clinical conditions, across England, Scotland and Wales, only. Counts between 1 and 9 have been suppressed, all other counts rounded to nearest 10. 41](#_Toc145528387)

[**Table S11:** Nation and meta, adjusted hazard ratios with 95% confidence intervals, for specific clinical conditions associated with severe COVID-19 outcomes, across England, Scotland and Wales, only. 44](#_Toc145528388)

[Autumn booster vaccination and COVID-19 therapeutics 46](#_Toc145528389)

[**Table S12:** Descriptive characteristics and rates of severe COVID-19 outcomes for individuals who received an Autumn 2022 booster and COVID-19 therapeutic treatment, either before or after booster vaccination, in Scotland only. 46](#_Toc145528390)

[Sensitivity analysis: Outcome under broad definitions 48](#_Toc145528391)

[**Table S13:** Nation and pooled sample characteristics and rates of broad severe COVID-19 outcomes for individuals who received an Autumn 2022 booster vaccination. Rates are number of events per 1,000 person-years. Counts between 1 and 9 have been suppressed, all other counts rounded to nearest 10. 48](#_Toc145528392)

[**Table S14:** Nation and meta, adjusted hazard ratios for vaccination, socio-demographics and clinical factors associated with severe COVID-19 outcomes under broad definitions. 58](#_Toc145528393)

[**Figure S2:** Meta, adjusted hazard ratios for broad and strict definitions of severe COVID-19 outcomes. * England, Scotland, Wales only. † Northern Ireland only. 65](#_Toc145528394)

[References 66](#_Toc145528395)

# Reporting and Methodology

## **Table S1:** ICD-10 codes for COVID-19 illness

| Code | Description |
| --- | --- |
| U07.1 | COVID-19, virus identified |
| U07.2 | COVID-19, virus not identified |

## **Table S2:** Reporting STROBE and RECORD checklists.

|  |  | **STROBE items** | **RECORD items** | **Location in manuscript where items are reported** |
| --- | --- | --- | --- | --- |
| **Title and abstract** |  |  |  |  |
|  | 1 | (a) Indicate the study’s design with a commonly used term in the title or the abstract (b) Provide in the abstract an informative and balanced summary of what was done and what was found | RECORD 1.1: The type of data used should be specified in the title or abstract. When possible, the name of the databases used should be included.  RECORD 1.2: If applicable, the geographic region and timeframe within which the study took place should be reported in the title or abstract.  RECORD 1.3: If linkage between databases was conducted for the study, this should be clearly stated in the title or abstract. | p. 1-4 |
| **Introduction** |  |  |  |  |
| Background rationale | 2 | Explain the scientific background and rationale for the investigation being reported |  | p. 5 |
| Objectives | 3 | State specific objectives, including any prespecified hypotheses |  | p. 5 |
| **Methods** |  |  |  |  |
| Study Design | 4 | Present key elements of study design early in the paper |  | P p. 6 |
| Setting | 5 | Describe the setting, locations, and relevant dates, including periods of recruitment, exposure, follow-up, and data collection |  | p. 6-9 |
| Participants | 6 | *(a) Cohort study* - Give the eligibility criteria, and the sources and methods of selection of participants. Describe methods of follow-up  *Case-control study* - Give the eligibility criteria, and the sources and methods of case ascertainment and control selection. Give the rationale for the choice of cases and controls  *Cross-sectional study* - Give the eligibility criteria, and the sources and methods of selection of participants  *(b) Cohort study* - For matched studies, give matching criteria and number of exposed and unexposed  *Case-control study* - For matched studies, give matching criteria and the number of controls per case | RECORD 6.1: The methods of study population selection (such as codes or algorithms used to identify subjects) should be listed in detail. If this is not possible, an explanation should be provided.  RECORD 6.2: Any validation studies of the codes or algorithms used to select the population should be referenced. If validation was conducted for this study and not published elsewhere, detailed methods and results should be provided.  RECORD 6.3: If the study involved linkage of databases, consider use of a flow diagram or other graphical display to demonstrate the data linkage process, including the number of individuals with linked data at each stage. | p. 6-8 |
| Variables | 7 | Clearly define all outcomes, exposures, predictors, potential confounders, and effect modifiers. Give diagnostic criteria, if applicable. | RECORD 7.1: A complete list of codes and algorithms used to classify exposures, outcomes, confounders, and effect modifiers should be provided. If these cannot be reported, an explanation should be provided. | p.7-8 |
| Data sources/ measurement | 8 | For each variable of interest, give sources of data and details of methods of assessment (measurement).  Describe comparability of assessment methods if there is more than one group |  | p. 6-8 |
| Bias | 9 | Describe any efforts to address potential sources of bias |  | p. 8-9 |
| Study size | 10 | Explain how the study size was arrived at |  | NA |
| Quantitative variables | 11 | Explain how quantitative variables were handled in the analyses. If applicable, describe which groupings were chosen, and why |  | p. 8-9 |
| Statistical methods | 12 | (a) Describe all statistical methods, including those used to control for confounding  (b) Describe any methods used to examine subgroups and interactions  (c) Explain how missing data were addressed  (d) *Cohort study* - If applicable, explain how loss to follow-up was addressed  *Case-control study* - If applicable, explain how matching of cases and controls was addressed  *Cross-sectional study* - If applicable, describe analytical methods taking account of sampling strategy  (e) Describe any sensitivity analyses |  | p. 8-9 |
| Data access and cleaning methods |  | .. | RECORD 12.1: Authors should describe the extent to which the investigators had access to the database population used to create the study population.  RECORD 12.2: Authors should provide information on the data cleaning methods used in the study. | p. 6-9 |
| Linkage |  | .. | RECORD 12.3: State whether the study included person-level, institutional-level, or other data linkage across two or more databases. The methods of linkage and methods of linkage quality evaluation should be provided. | p. 6-9 |
| **Results** |  |  |  |  |
| Participants | 13 | (a) Report the numbers of individuals at each stage of the study (*e.g.*, numbers potentially eligible, examined for eligibility, confirmed eligible, included in the study, completing follow-up, and analysed)  (b) Give reasons for non-participation at each stage.  (c) Consider use of a flow diagram | RECORD 13.1: Describe in detail the selection of the persons included in the study (*i.e.,* study population selection) including filtering based on data quality, data availability and linkage. The selection of included persons can be described in the text and/or by means of the study flow diagram. | p. 10-11 |
| Descriptive data | 14 | (a) Give characteristics of study participants (*e.g.*, demographic, clinical, social) and information on exposures and potential confounders  (b) Indicate the number of participants with missing data for each variable of interest  (c) *Cohort study* - summarise follow-up time (*e.g.*, average and total amount) |  | p. 10-11 |
| Outcome data | 15 | *Cohort study* - Report numbers of outcome events or summary measures over time  *Case-control study* - Report numbers in each exposure category, or summary measures of exposure  *Cross-sectional study* - Report numbers of outcome events or summary measures |  | p. 10-11 |
| Main results | 16 | (a) Give unadjusted estimates and, if applicable, confounder-adjusted estimates and their precision (e.g., 95% confidence interval). Make clear which confounders were adjusted for and why they were included  (b) Report category boundaries when continuous variables were categorized  (c) If relevant, consider translating estimates of relative risk into absolute risk for a meaningful time period |  | p. 10-11 |
| Other analyses | 17 | Report other analyses done—e.g., analyses of subgroups and interactions, and sensitivity analyses |  | p. 10-11 |
| **Discussion** |  |  |  |  |
| Key results | 18 | Summarise key results with reference to study objectives |  | p. 11 |
| Limitations | 19 | Discuss limitations of the study, taking into account sources of potential bias or imprecision. Discuss both direction and magnitude of any potential bias | RECORD 19.1: Discuss the implications of using data that were not created or collected to answer the specific research question(s). Include discussion of misclassification bias, unmeasured confounding, missing data, and changing eligibility over time, as they pertain to the study being reported. | p. 12 |
| Interpretation | 20 | Give a cautious overall interpretation of results considering objectives, limitations, multiplicity of analyses, results from similar studies, and other relevant evidence |  | p. 12 |
| Generalisability | 21 | Discuss the generalisability (external validity) of the study results |  | p. 12 |
| **Other Information** |  |  |  |  |
| Funding | 22 | Give the source of funding and the role of the funders for the present study and, if applicable, for the original study on which the present article is based |  | p. 3,9,13 |
| Accessibility of protocol, raw data, and programming code |  | .. | RECORD 22.1: Authors should provide information on how to access any supplemental information such as the study protocol, raw data, or programming code. | p. 13 |

STROBE: Strengthening the Reporting of Observational studies in Epidemiology. RECORD: Reporting of Studies Conducted using Observational Routinely-collected Data. NA: not applicable.

## **Table S3:** QCOVID risk groups and codes.

| **QCOVID risk group** | **Code** |
| --- | --- |
| Atrial fibrillation | Q_DIAG_AF |
| Asthma | Q_DIAG_ASTHMA |
| Blood cancer | Q_DIAG_BLOOD_CANCER |
| Heart failure | Q_DIAG_CCF |
| Cerebral palsy | Q_DIAG_CEREBALPALSY |
| Coronary heart disease | Q_DIAG_CHD |
| Cirrhosis | Q_DIAG_CIRRHOSIS |
| Congenital heart disease | Q_DIAG_CONGEN_HD |
| COPD | Q_DIAG_COPD |
| Dementia | Q_DIAG_DEMENTIA |
| Diabetes type 1 | Q_DIAG_DIABETES_1 |
| Diabetes type 2 | Q_DIAG_DIABETES_2 |
| Epilepsy | Q_DIAG_EPILEPSY |
| Fracture | Q_DIAG_FRACTURE |
| Neurological disorder | Q_DIAG_NEURO |
| Parkinson’s | Q_DIAG_PARKINSONS |
| Pulmonary hypertension | Q_DIAG_PULM_HYPER |
| Pulmonary rare | Q_DIAG_PULM_RARE |
| Peripheral vascular disease | Q_DIAG_PVD |
| Rheumatoid arthritis or SLE | Q_DIAG_RA_SLE |
| Respiratory cancer | Q_DIAG_RESP_CANCER |
| Severe mental illness | Q_DIAG_MENT_ILL |
| Sickle cell disease | Q_DIAG_SICKLE_CELL |
| Stroke/TIA | Q_DIAG_STROKE |
| Thrombosis or pulmonary embolus | Q_DIAG_VTE |
| Care housing category | Q_HOME_CAT |
| Learning disability or Down's | Q_LEARN_CAT |
| Kidney disease | Q_DIAG_CKD_LEVEL |
| More information on codes: <https://github.com/EAVE-II/EAVE-II-data-dictionary>  Ref: Clift, A.K., et al. Living risk prediction algorithm (QCOVID) for risk of hospital admission and mortality from coronavirus 19 in adults: national derivation and validation cohort study. BMJ 371, m3731 (2020). | |

# Data sources

In England, we used the nationally representative Oxford-Royal College of General Practitioners (RCGP) Research and Surveillance Centre (RSC) database, which has a near real-time feed of primary care data and covers around 32% of the English population (N>19 million). Data were pseudonymised in the OxfoRd Clinical Informatics Digital Hub (ORCHID) TRE.^1^ Pseudonymisation was conducted using a National Health Service (NHS) Digital approved process, allowing pseudonymised NHS numbers (unique national IDs) to link individual level data to other datasets to supplement primary care data; these datasets included the national immunisation management service for vaccine uptake, Hospital Episode Statistics for hospitalisation, and ONS data for certified cause of death.

In Northern Ireland, vaccination data from the Vaccine Management System were linked to relevant datasets using an anonymised study identifier that replaced each individual’s unique health and care number to construct the cohort, covering 1.9 million individuals (entire population). These datasets were: population data from the National Health Authority Information System (recording eligibility for health care in Northern Ireland, which included date of death); and medications dispensed by community pharmacists from the enhanced prescribing database. COVID-19 related hospitalisations were identified using the Patient Administration System, which captured all acute hospital sites in Northern Ireland. Primary care consultation and diagnostic data were not available for Northern Ireland; therefore, medication dispensation was used as a proxy measure for comorbidities.

In Scotland, the Early Pandemic Evaluation and Enhanced Surveillance of COVID-19 (EAVE II) is a Scotland-wide COVID-19 surveillance platform which has been used to identify and predict adults at increased risk of severe COVID-19 outcomes leading to hospitalisation and death.^2-6^ We used the Community Health Index (CHI) number, which is a unique identifier for each population member and used in all healthcare contacts across NHS Scotland, to deterministically link primary care data on 5.4 million people (around 99% of the population) from 940 general practices, secondary care data from Scottish Morbidity Record 01 (SMR01) and Rapid Preliminary Inpatient Data (RAPID), vaccination status data from the Turas Vaccination Management Tool (TVMT), and mortality data from National Records of Scotland (NRS).

In Wales, the Secure Anonymised Information Linkage (SAIL) Databank TRE^7,8^ used Anonymised Information Linkage Field, covering 3·2 million individuals (entire population), which is a pseudonymised unique identifier used in all healthcare contacts across Wales, to link population-level primary care records of 329 (84%) of 391 general practices across Wales, and all hospital admissions and deaths for the entire population from a cohort designed for studying COVID-19 related outcomes.^9^

# Protocol

## **Research aims**

1. What is the prevalence of COVID-19 associated hospitalisations or deaths post-autumn booster COVID-19 vaccine dose across individual UK nations and the UK overall?
2. What are the risk factors (demographic, ethnic group, socioeconomic, immunological and clinical features) of individuals with post-autumn booster dose COVID-19 severe outcomes?
3. Does the risk of severe COVID-19 outcomes vary by vaccine type, duration since vaccination and use of COVID-19 therapeutics?

## **Cohort and sample design**

A cohort of the general population of all those who have had an autumn booster dose:

- Alive and living on 1^st^ September 2022
- Have primary care records prior to 1^st^ September 2022
- Aged between 18 and 110 on 1^st^ September 2022
- Has previously received 3 doses prior to 1^st^ September 2022
- Has received an autumn booster dose (MD or PB only)

Follow-up:

- The study window starts on 1^st^ September 2022 and ends on 31^st^ December 2022
- An individual enters the study day 14 after receiving their autumn booster dose
- Follow-up time follows calendar time, so day 0 is 1^st^ September 2022, an individual’s start time (tstart) will then be the number of days since 1^st^ September 2022. For example:


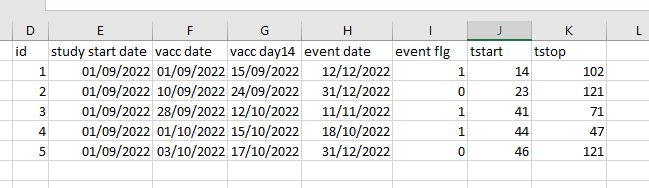


## **Outcome**

- Main outcome is first date of hospitalisation or death due to COVID-19 at least 14 days post-autumn booster vaccination
- The plan is to use strict definitions for COVID-19 hospitalisation and death for our main outcome, followed-by a sensitive analysis using two broader definitions
- Strict definitions:
  - COVID-19 hospitalisation is defined as an emergency hospital admission, with an ICD-10 code for COVID-19 in the first position of the first episode, after removing ICD-10 codes starting with R or Z
  - COVID-19 death is defined as a death with COVID-19 being listed as the primary cause of death
- Broader definitions:
  - COVID-19 hospitalisation is defined as an emergency hospital admission with an ICD-10 code for COVID-19 in any position of the first episode (i.e., admitted due to or with COVID-19)
  - COVID-19 death is defined as a death with COVID-19 being listed in any position relating to the death (i.e., primary, secondary cause, or underlying factor)
- Observations are censored before the end of the study window if the person:
  - Moved out of the respective nation
  - Died due to other causes

## **Exposure**

Exposure is time under autumn booster dose for each type of vaccine. Measures needed to define exposure are:

- Date of the autumn booster vaccination
- Name of vaccine: Pfizer-BioNTech (PB), Moderna (MD)
- For pervious doses, all the vaccines can be included

## **Covariates**

- Age: 18-49, 50-64, 65-79, 80+
- Sex: Male, Female
- Area deprivation quintile on 1^st^ September 2022
  - IMD (or respective national indicator), 1 is Most deprived, 5 is least deprived
- Ethnicity:
  - Categorised as per ONS five categories: White, Asian, Black, Mixed, Other
  - Code missing as Unknown
- Number of co-morbidities:
  - England, Scotland, Wales: Number of QCOVID (version 1) indicators as of 1^st^ September 2022, excluding BMI: 0, 1, 2, 3, 4, 5+
  - Northern Ireland: Number of BNF chapters with repeat prescriptions as per previous work
- Most recently recorded BMI in the last 5 years up to 1^st^ September 2022.
  - Five categories: <18.5, 18.5-24.9, 25-29.9, 30-39.9, 40+.
  - Wales: Imputed at 45% missing using a single imputation based on log (BMI) as the outcome and the following as covariates:
    - Simplified outcomes: ever had positive PCR, ever had COVID-19 hospital admission or death.
    - Simplified exposures: dose 2 name, booster name, with "not received" as a category.
    - All covariates used for analysis, including individual QCovid measures
  - Northern Ireland: BMI not available
- Time since previous vaccination: 0-23 weeks 6 days, 24 weeks or more
- Urban/rural classification of residence on 1^st^ September 2022. Categories collapsed:
  - Urban = (Urban city and town, Urban city and town in a sparse setting).
  - Rural = (Rural town and fringe, Rural town and fringe in a sparse setting, Rural village and dispersed, Rural village and dispersed in a sparse setting).
- NHS appropriate geography (e.g., Health Board).
- Number of members in the same household: 1, 2, 3-5, 6-10, 11+.
- COVID-19 therapeutics:
  - None
  - Only monoclonal antibody
  - Only antiviral treatment
  - Multiple
- Time of the COVID-19 therapeutics:
  - Pre autumn booster
  - Post autumn booster
  - Per and post autumn booster
  - No

## **Exploratory analysis**

For the full population cohort, to see what information we have before we implement the study:

- Plot weekly counts for:
  - Vaccination dose and type
  - COVID-19 hospitalisation, COVID-19 death
  - Move out of the nation (Wales only), death due to other causes
- Plot timings between the last dose of vaccine and autumn booster dose
- Empirical cumulative incidence of vaccination uptake by age groups: 18-64, 65-79, 80-110
- Univariate summary of characteristics
- Empirical cumulative incidence of COVID-19 hospitalisation/death by vaccination status and age groups: 18-64, 65-79, 80-110

## **Describe cohort characteristics**

Using the full cohort, make four tables of descriptive characteristic summaries for:

- For those who had autumn booster dose, total counts and column percentages, and stratified by booster vaccine name:
- Number of events and rate per 1,000 years whilst under autumn booster dose:

## **Main analysis of severe COVID-19 outcomes post autumn vaccination**

- Aim is to identify overall associations of the outcome with the vaccine, social, demographic, and health characteristics
- Outcome and censoring are defined above
- Model time to outcome using Cox PH, with follow-up time being measured as days since start of the study window, 1^st^ September 2022
- An individual enters the study on day 14 after their vaccination
- Example: if an individual gets vaccinated on 10^th^ September, they’ll enter the study on the 24^th^, which will be coded as tstart = 23
- To capture the relative performance of the vaccine, use a time-varying covariate to capture the time since vaccination. We use the following categorical, two-week, intervals:
  - Day 14-27 (i.e., 2-3 weeks)
  - Day 28-41
  - Day 42-55
  - Day 56-69
  - Day 70-83
  - Day 84+ (i.e., 12 weeks +)
- This way, the underlying hazard is the instantaneous hazard of the event each calendar day during the study period. As people get vaccinated at different points over the period, we need a way of separating out how effective the vaccine is doing vs the background rate of events
- We carry this out as an overall analysis, stratifying the baseline by the two vaccines people had received, as well as perform vaccine-specific subset analyses

## **Specific clinical indicator analysis**

- Aim is to identify risks associated with the specific clinical indicators rather than simply have them in a combined score like in the main analysis
- Use the analytical approach as the Main analysis, but instead of having the “number of co-morbidities" as a covariate, we instead include one clinical indicator at a time, fitting a separate adjusted model each time
- This means that we are finding the specific effects for each health condition, uncontrolled for the other health conditions, but controlled for the following background socio-demographic characteristics:
  - Sex
  - Age
  - Time since previous vaccination
  - Household size
  - Deprivation quintile
  - Urban/rural measure
- Only do this as an overall analysis, no need to repeat for specific vaccines

# Patient and public involvement with this study

**Table S4**: GRIPP2 reporting checklist (short form)

| Section and topic | Item | Reported on page No |
| --- | --- | --- |
| 1: Aim | Report the aim of PPI in the study | 8 |
| 2: Methods | Provide a clear description of the methods used for PPI in the study | 8 |
| 3: Study results | Outcomes—Report the results of PPI in the study, including both positive and negative outcomes | 9 |
| 4: Discussion and conclusions | Outcomes—Comment on the extent to which PPI influenced the study overall. Describe positive and negative effects | 10 |
| 5: Reflections/critical perspective | Comment critically on the study, reflecting on the things that went well and those that did not, so others can learn from this experience | 11 |

PPI=patient and public involvement

## **GRIPP2 Reporting Guidelines: Short Form**

Aim

There were three primary aims of patient and public involvement (PPI) in this study. Initially, we intended to align project activity with UK-wide public perspectives and information needs on booster vaccine breakthrough, by embedding PPI in decision-making structures and throughout the project. Secondly, we aimed to engage and communicate with audiences impacted by policy decisions resulting from the research (people at high risk of vaccine breakthrough, and their GPs). Thirdly, we sought to contribute to shared best practice of Patient and Public Involvement.

Methods

This study used data from several sources across the UK. In Scotland, data was routinely collected from the “Early Pandemic Evaluation and Enhanced Surveillance of COVID-19” (EAVE II) platform: <https://www.ed.ac.uk/usher/eave-ii/about-eave-ii/introduction-to-eave-ii>. In England, the Oxford Royal College of General Practitioners (RCGP) Research and Surveillance Centre (RSC) Clinical Informatics Digital Hub dataset via ORCHID was used. In Wales, the Controlling COVID-19 through enhanced population surveillance and intervention (ConCOV) platform was utilised via SAIL Databank. Lastly, we accessed Northern Ireland data through the Honest Broker Service which is a trusted research environment that enables controlled access to national health datasets, including the Vaccine Management System.

This research bid originated from extensive PPI input in our initial Vaccine Breakthrough project (VB1). Lynn Laidlaw [LL] and Deb Smith [DS] were invited to act as PPI leads for this project, given their lived experiences, PPI expertise as part of the EAVE II Public Advisory Group (PAG) and familiarity with National Core Studies Immunity (NCSi) research. Alongside, PPI co-ordinator Dr Lana Woolford [LW] and PPI Payments Administrator Laura Gonzalez-Rienda, they formed the PPI team for this study. In addition, wider collaboration with the Project Manager, Natalia Reglinska-Matveyev (NRM) to coordinate meeting attendance/actions and Communications & Engagement Officer, Gabriella Linning (GL), PPI related actions were prioritised and outputs were disseminated across media channels.

The PPI team has been involved in supporting PPI objectives and deliverables, informing key decision making, offering a public perspective on the grant application as well as interpretation of findings, evaluating PPI elements of the project, contributing to dissemination activities and authoring this GRIPP2 appendix. This work was carried out remotely, either using videoconferencing or asynchronously via email. Public members of the PPI Team were rewarded for time and expertise shared, in line with National Institute for Health and Care Research (NIHR) guidelines.

Results

Based on the aims outlined above, the PPI Team carried out PPI deliverables in line with the research cycle as summarised in **Table S5**.

## **Table S5:** PPI deliverables in line with the research cycle.

| Area of research cycle | Summary of deliverables |
| --- | --- |
| Identifying patient and public priorities | Identify research priorities from a wider public/patient perspective and research questions to strengthen the relevance of this study. |
| Grant development | Co-produce a PPI strategy for the project alongside project staff, based on reflections from VB1 and to shape the grant application, including the statistical analysis plan. |
| Undertaking project | PPI Leads to attend regular PPI meetings and monthly steering meetings to review content and inform key decision making. |
| Design | Conduct own research, assist in workshop planning and make suggestions for improving GP communication channels |
| Analysis and interpretation | Discuss the preliminary findings within the project team alongside wider EAVE II PAG. |
| Dissemination | Lead on presenting the PPI elements of the project to the wider EAVE II PAG. |
| Implementation | Discuss the implementation of results by offering training to researchers and involvement professionals/contributors as part of the NCS programme. |
| Evaluation | Reflect on the PPI activity in the project from different perspectives under the themes of Personal, Project and Structural impacts. |

This work has directly resulted in shaping two of the four main research aims. Specifically, on the inclusion of immunological data (for individuals with this type of health condition) when observing risk and the analysis of breakthrough risk in people eligible for COVID-19 therapeutics who are likely to be prioritised for vaccines. This input was also used in the early stages to produce a project summary and communications plan for the research grant proposal. Regular PPI input in early stages of project design was essential to inform a co-produced PPI strategy.

Similarly, contribution in dedicated PPI meetings or involvement in steering group sessions has provided opportunity to inform researchers about a “public perspective” and led to greater team accountability over time. Another impact was LL and DS researching gaps on GP communication channels (doing a mini literature review) for accessible health information to create a co-produced workshop to address these. Key research results were presented to the wider EAVE II PAG to inform the creation of a PPI report and/or NCSi case study.

We have also liaised with the PPI co-ordinator and contributors to create a full “summary in plain English” and infographic to communicate key findings. In addition, co-ordinated with external and project partners to disseminate these materials across various communication channels such as, social media and internal/external facing newsletters. Likewise, requesting and collating quotes from PPI members to create thumbnail graphics. An example of the summary and infographic can be accessed [here.](https://www.ed.ac.uk/usher/eave-ii/connected-projects/vaccine-breakthrough-project/our-publication/severe-covid-19-post-vaccination-primary-booster)

Discussion and Conclusion

The PPI strategy was used to improve practices such as, training provisions carried out in the original Vaccine Breakthrough (VB1) project. Regular contribution in day-to-day activities and alternate weekly meetings allowed for research and associated activities to shape the direction of key stages from a patient perspective. Innovative co-designed PPI was used to address wider structural questions and to create accessible public facing resources.

We felt that the media channels we used such as, websites, mainstream, or social media whilst available for patients seeking health information who may be at high risk of vaccine breakthrough, didn’t provide the individualised information that people wanted. Other barriers faced by people included health or digital literacy. We wanted to highlight the question, are GPs a viable channel for sharing individualised information with their patients and do GP’s have access to the type of information required to facilitate this? This felt like a significant research gap, particularly in a COVID-19 context.

As a result, we designed and recruited for a workshop to explore barriers and enablers of these GP communication channels. Unfortunately, we were unable to recruit regular or deep-end GPs, but did meet and receive a valuable perspective from an academic GP, with notes and reflections from the meeting shared with the project team. In addition, compiling perspectives from professional services staff and PPI leads. This revealed useful information about project activities and its wider context amongst NCSi studies.

Reflections

Contributors have felt supported, valued and rewarded throughout the study. The work was “meaningful" and they observed the value of a “trusted partnership” within the team. This led to more productive and pragmatic work. As a result, new skills were learned such as, “doing my own research” and greater enjoyment of working on wider issues. Similarly, contracts of engagement have been “really positive” for the structure of project delivery. However, there were delays in progression of study objectives that led to challenges in collaboration and reduced deliverables. This was primarily due to the short length of the study that led to significant time pressures. Deliverables may have been able to be met if the study had more time. Another challenge was reflecting the range of PPI experience, working in a larger group led to difficulty in resolving all queries. Also, the similarity of previous NCSi PPI work resulted in some confusion for contributors. As stated by contributors, it can be challenging to keep track of multiple projects!

Operationally, researchers noted the success of discussions about broader issues rather than details in PAG meetings. Yet, email communication and smaller discussions have also worked well. This was particularly relevant in the grant application stage. An additional impact was on the NCSi training programme that led to impact at a national level. However, certain deliverables were unable to fully met such as, GP workshops and dissemination work around publications as papers were not written to action these. This had an impact on the results and interpretation session that led to “frustration” given the ambitious PPI plans of the study. This also showcases an under resourcing of a central PPI lead within a short-term project.

Future PPI should consider the feasibility of practical implementation of results and acknowledge differences of PPI contributors/peer researcher’s dynamics on study progression. Also, consider introducing a “buddy system” or debriefs after sessions to address any questions or concerns. For researchers, planning smaller meetings with researchers (rather than larger sessions) may improve communication between colleagues and lead to more efficient decision making. If delays are predicted, re-structuring PPI via project design to ensure impact is not solely reliant on researcher activity can be beneficial. Lastly, employing a more tailored approach to mentorship (with funds to support this in the budget) for new or experienced members is recommended.

# Underlying nation results

## **Table S6:** Number of people entering the study within each nation at 2-week intervals, along with number having the event.

| **Time since study start (weeks)** | **England** | | **Northern Ireland** | | **Scotland** | | **Wales** | |
| --- | --- | --- | --- | --- | --- | --- | --- | --- |
|  | At risk | Events | At risk | Events | At risk | Events | At risk | Events |
| 0 | 199,080 | - | 280 | - | 10,460 | - | 11,010 | - |
| 2 | 199,080 | - | 280 | - | 10,460 | - | 11,010 | - |
| 4 | 199,080 | - | 280 | - | 119,820 | <10 | 95,720 | <10 |
| 6 | 941,220 | 40 | 43,010 | - | 465,490 | 40 | 207,540 | 10 |
| 8 | 1,974,210 | 90 | 145,670 | <10 | 823,430 | 60 | 367,510 | 30 |
| 10 | 2,979,330 | 100 | 313,520 | 20 | 1,145,690 | 80 | 526,390 | 40 |
| 12 | 3,674,860 | 100 | 378,230 | 30 | 1,425,420 | 90 | 646,290 | 40 |
| 14 | 4,060,210 | 140 | 430,170 | 40 | 1,671,650 | 160 | 738,440 | 70 |
| 16 | 4,241,300 | 400 | 448,110 | 60 | 1,802,610 | 270 | 794,590 | 140 |
| 17.3 (study end) | 4,305,190 | 1,110 | 453,680 | 80 | 1,821,050 | 130 | 814,160 | 110 |

## **Table S7:** Nation sample characteristics and rates of severe COVID-19 outcomes for individuals who received an Autumn 2022 booster vaccination. Rates are number of events per 1,000 person-years. Counts between 1 and 9 have been suppressed, all other counts rounded to nearest 10.

|  | **England** | | **Northern Ireland** | | **Scotland** | | **Wales** | | **Pooled** | |
| --- | --- | --- | --- | --- | --- | --- | --- | --- | --- | --- |
|  | **n (%)** | **Outcome (rate)** | **n (%)** | **Outcome (rate)** | **n (%)** | **Outcome (rate)** | **n (%)** | **Outcome (rate)** | **n (%)** | **Outcome (rate)** |
| **Overall** | | | | | | | | | | |
| **Total** | 4,348,220 (100.0%) | 1,990 (2.8) | 455,290 (100.0%) | 240 (3.4) | 1,829,690 (100.0%) | 820 (2.9) | 818,690 (100.0%) | 450 (3.3) | 7,451,890 (100.0%) | 3,500 (2.9) |
| **Autumn 2022 vaccination** | | | | | | | | | | |
| Comirnaty | 2,498,240 (57.5%) | 760 (2.3) | 444,900 (97.7%) | 230 (3.4) | 808,130 (44.2%) | 260 (2.5) | 397,770 (48.6%) | 120 (2.4) | 4,149,040 (55.7%) | 1,370 (2.5) |
| Spikevax | 1,849,980 (42.5%) | 1,230 (3.1) | 10,390 (2.3%) | - | 1,021,560 (55.8%) | 560 (3.2) | 420,920 (51.4%) | 330 (3.9) | 3,302,840 (44.3%) | 2,120 (3.2) |
| **Number of previous COVID-19 vaccinations** | | | | | | | | | | |
| 3 | 3,133,430 (72.1%) | 520 (1.1) | 345,140 (75.8%) | 80 (1.6) | 1,349,740 (73.8%) | 210 (1.1) | 584,860 (71.4%) | 110 (1.2) | 5,413,160 (72.6%) | 920 (1.1) |
| 4 | 1,121,880 (25.8%) | 1,380 (6.3) | 105,930 (23.3%) | 150 (8.5) | 453,910 (24.8%) | 580 (6.7) | 212,420 (25.9%) | 300 (7.0) | 1,894,140 (25.4%) | 2,410 (6.6) |
| 5 | 92,910 (2.1%) | 90 (5.3) | 4,220 (0.9%) | - | 26,040 (1.4%) | 30 (7.3) | 21,410 (2.6%) | 40 (9.7) | 144,580 (1.9%) | 160 (6.2) |
| **Time since previous COVID-19 vaccination** | | | | | | | | | | |
| <24 weeks | 649,840 (14.9%) | 880 (6.7) | 74,020 (16.3%) | 120 (8.7) | 292,640 (16.0%) | 410 (6.8) | 152,130 (18.6%) | 250 (7.3) | 1,168,630 (15.7%) | 1,670 (6.9) |
| ≥24 weeks | 3,698,380 (85.1%) | 1,110 (1.9) | 381,270 (83.7%) | 120 (2.1) | 1,537,050 (84.0%) | 410 (1.9) | 666,560 (81.4%) | 200 (2.0) | 6,283,260 (84.3%) | 1,830 (1.9) |
| **Sex** | | | | | | | | | | |
| Female | 2,343,540 (53.9%) | 980 (2.5) | 244,670 (53.7%) | 110 (2.8) | 1,010,620 (55.2%) | 410 (2.6) | 441,990 (54.0%) | 220 (2.9) | 4,040,810 (54.2%) | 1,710 (2.6) |
| Male | 2,004,680 (46.1%) | 1,010 (3.1) | 210,620 (46.3%) | 130 (4.1) | 819,070 (44.8%) | 420 (3.4) | 376,700 (46.0%) | 230 (3.8) | 3,411,070 (45.8%) | 1,790 (3.3) |
| **Age** | | | | | | | | | | |
| 18-49 years | 499,590 (11.5%) | 30 (0.4) | 51,770 (11.4%) | - | 268,050 (14.6%) | 20 (0.5) | 100,690 (12.3%) | 10 (0.9) | 920,100 (12.3%) | 60 (0.5) |
| 50-64 years | 1,620,370 (37.3%) | 140 (0.6) | 166,430 (36.6%) | 10 (0.5) | 685,110 (37.4%) | 80 (1.0) | 295,290 (36.1%) | 50 (1.2) | 2,767,200 (37.1%) | 280 (0.8) |
| 65-79 years | 1,612,450 (37.1%) | 600 (2.0) | 173,250 (38.1%) | 80 (2.8) | 664,040 (36.3%) | 320 (2.6) | 310,150 (37.9%) | 150 (2.6) | 2,759,880 (37.0%) | 1,150 (2.3) |
| ≥80 years | 615,810 (14.2%) | 1,220 (10.1) | 63,850 (14.0%) | 140 (12.9) | 212,500 (11.6%) | 410 (9.5) | 112,550 (13.7%) | 240 (10.4) | 1,004,710 (13.5%) | 2,010 (10.2) |
| **Ethnicity** | | | | | | | | | | |
| White | 3,796,620 (87.3%) | 1,790 (2.8) | - | - | 1,334,660 (72.9%) | 720 (3.5) | 792,560 (96.8%) | 450 (3.4) | 5,923,840 (84.7%) | 2,960 (3.0) |
| Asian | 171,010 (3.9%) | 40 (1.6) | - | - | 21,000 (1.1%) | 10 (3.9) | 12,880 (1.6%) | - | 204,880 (2.9%) | 60 (1.8) |
| Black | 51,420 (1.2%) | 10 (1.8) | - | - | 3,140 (0.2%) | 0 (0.0) | 2,100 (0.3%) | 0 (0.0) | 56,660 (0.8%) | 10 (1.2) |
| Mixed | 31,140 (0.7%) | - | - | - | 3,810 (0.2%) | - | 2,940 (0.4%) | - | 37,890 (0.5%) | 10 (1.7) |
| Other | 24,080 (0.6%) | - | - | - | 3,620 (0.2%) | 0 (0.0) | 2,580 (0.3%) | 0 (0.0) | 30,280 (0.4%) | 0 (0.0) |
| Unknown | 273,960 (6.3%) | 130 (2.9) | - | - | 463,470 (25.3%) | 90 (1.3) | 5,640 (0.7%) | - | 743,060 (10.6%) | 220 (2.0) |
| **BMI** | | | | | | | | | | |
| <18.5 | 75,900 (1.7%) | 130 (9.9) | - | - | 17,400 (1.0%) | 40 (14.1) | 14,220 (1.7%) | 20 (9.7) | 107,510 (1.5%) | 180 (10.0) |
| 18.5-24.9 | 1,388,650 (31.9%) | 770 (3.3) | - | - | 251,670 (13.8%) | 210 (5.0) | 205,570 (25.1%) | 140 (3.9) | 1,845,890 (26.4%) | 1,110 (3.6) |
| 25.0-29.9 | 1,572,030 (36.2%) | 640 (2.4) | - | - | 1,069,310 (58.4%) | 350 (2.2) | 277,200 (33.9%) | 160 (3.5) | 2,918,540 (41.7%) | 1,150 (2.4) |
| 30.0-39.9 | 1,115,970 (25.7%) | 410 (2.2) | - | - | 404,030 (22.1%) | 200 (3.2) | 275,530 (33.7%) | 110 (2.4) | 1,795,540 (25.7%) | 710 (2.4) |
| ≥40.0 | 195,670 (4.5%) | 60 (1.8) | - | - | 87,280 (4.8%) | 30 (2.3) | 46,170 (5.6%) | 30 (3.5) | 329,120 (4.7%) | 110 (2.2) |
| **Number of QCovid risk groups** | | | | | | | | | | |
| 0 | 1,567,960 (36.1%) | 160 (0.6) | - | - | 917,860 (50.2%) | 110 (0.8) | 354,220 (43.3%) | 60 (1.0) | 2,840,040 (40.6%) | 330 (0.8) |
| 1 | 1,168,760 (26.9%) | 270 (1.4) | - | - | 572,230 (31.3%) | 220 (2.5) | 241,220 (29.5%) | 70 (1.8) | 1,982,220 (28.3%) | 560 (1.7) |
| 2 | 704,880 (16.2%) | 350 (2.9) | - | - | 212,180 (11.6%) | 200 (5.7) | 120,720 (14.7%) | 100 (4.8) | 1,037,770 (14.8%) | 650 (3.7) |
| 3 | 431,530 (9.9%) | 360 (4.7) | - | - | 81,180 (4.4%) | 150 (10.3) | 57,010 (7.0%) | 80 (7.9) | 569,720 (8.1%) | 580 (5.8) |
| 4 | 242,600 (5.6%) | 310 (7.1) | - | - | 30,950 (1.7%) | 80 (14.4) | 26,350 (3.2%) | 60 (11.8) | 299,890 (4.3%) | 450 (8.4) |
| ≥5 | 232,490 (5.3%) | 550 (13.1) | - | - | 15,300 (0.8%) | 60 (19.7) | 19,170 (2.3%) | 80 (22.8) | 266,960 (3.8%) | 690 (14.2) |
| **Number of BNF risk groups** | | | | | | | | | | |
| 0 | - | - | 95,590 (21.0%) | - | - | - | - | - | 95,590 (21.0%) | 0 (0.0) |
| 1 | - | - | 86,530 (19.0%) | 20 (1.2) | - | - | - | - | 86,530 (19.0%) | 20 (1.5) |
| 2 | - | - | 83,360 (18.3%) | 20 (1.2) | - | - | - | - | 83,360 (18.3%) | 20 (1.6) |
| 3 | - | - | 68,430 (15.0%) | 40 (3.8) | - | - | - | - | 68,430 (15.0%) | 40 (3.7) |
| 4 | - | - | 50,490 (11.1%) | 40 (5.3) | - | - | - | - | 50,490 (11.1%) | 40 (5.0) |
| 5 | - | - | 33,680 (7.4%) | 40 (7.7) | - | - | - | - | 33,680 (7.4%) | 40 (7.5) |
| ≥6 | - | - | 37,200 (8.2%) | 70 (12.6) | - | - | - | - | 37,200 (8.2%) | 70 (11.9) |
| **Household size** | | | | | | | | | | |
| 1 person | - | - | 77,350 (17.0%) | 70 (5.5) | 632,700 (34.6%) | 370 (3.7) | 155,570 (19.0%) | 130 (4.9) | 865,620 (27.9%) | 570 (4.1) |
| 2 people | - | - | 153,480 (33.7%) | 100 (3.9) | 697,490 (38.1%) | 300 (2.7) | 340,360 (41.6%) | 180 (3.1) | 1,191,330 (38.4%) | 580 (3.0) |
| 3-5 people | - | - | 197,280 (43.3%) | 60 (2.1) | 467,190 (25.5%) | 100 (1.6) | 285,800 (34.9%) | 90 (2.0) | 950,270 (30.6%) | 250 (1.9) |
| 6-10 people | - | - | 22,580 (5.0%) | - | 22,070 (1.2%) | 10 (3.6) | 24,300 (3.0%) | 10 (2.9) | 68,950 (2.2%) | 20 (2.0) |
| ≥11 people | - | - | 4,600 (1.0%) | - | 10,240 (0.6%) | 40 (16.0) | 12,650 (1.5%) | 50 (17.0) | 27,490 (0.9%) | 90 (15.9) |
| **Socioeconomic deprivation quintile** | | | | | | | | | | |
| 5th (Least) | 1,200,860 (27.6%) | 510 (2.6) | 113,660 (25.0%) | 50 (2.8) | 420,010 (23.0%) | 140 (2.2) | 207,540 (25.4%) | 110 (3.2) | 1,942,060 (26.1%) | 820 (2.6) |
| 4th | 1,041,070 (23.9%) | 460 (2.6) | 100,480 (22.1%) | 60 (3.8) | 407,740 (22.3%) | 140 (2.3) | 172,520 (21.1%) | 80 (2.8) | 1,721,810 (23.1%) | 740 (2.7) |
| 3rd | 894,460 (20.6%) | 380 (2.6) | 89,490 (19.7%) | 40 (2.7) | 382,050 (20.9%) | 150 (2.6) | 160,690 (19.6%) | 90 (3.3) | 1,526,680 (20.5%) | 660 (2.7) |
| 2nd | 712,070 (16.4%) | 340 (2.9) | 84,410 (18.5%) | 50 (3.7) | 338,990 (18.5%) | 200 (3.8) | 153,300 (18.7%) | 90 (3.5) | 1,288,760 (17.3%) | 680 (3.3) |
| 1st (Most) | 499,760 (11.5%) | 300 (3.6) | 67,250 (14.8%) | 50 (4.4) | 280,910 (15.4%) | 190 (4.4) | 124,630 (15.2%) | 90 (4.1) | 972,550 (13.1%) | 620 (4.0) |
| **Rural/urban area classification** | | | | | | | | | | |
| Urban | 3,304,990 (76.0%) | 1,610 (2.9) | 292,190 (64.2%) | 160 (3.6) | 1,534,030 (83.8%) | 720 (3.1) | 563,000 (68.8%) | 340 (3.6) | 5,694,210 (76.4%) | 2,840 (3.1) |
| Rural | 1,043,230 (24.0%) | 380 (2.2) | 163,100 (35.8%) | 80 (3.1) | 295,660 (16.2%) | 100 (2.2) | 255,690 (31.2%) | 110 (2.7) | 1,757,670 (23.6%) | 660 (2.3) |
| **Comirnaty** | | | | | | | | | | |
| **Total** | 2,498,240 (100.0%) | 760 (2.3) | 444,900 (100.0%) | 230 (3.4) | 808,130 (100.0%) | 260 (2.5) | 397,770 (100.0%) | 120 (2.4) | 4,149,040 (100.0%) | 1,370 (2.5) |
| **Number of previous COVID-19 vaccinations** | | | | | | | | | | |
| 3 | 2,026,170 (81.1%) | 260 (1.0) | 337,790 (75.9%) | 80 (1.6) | 637,210 (78.9%) | 90 (1.2) | 318,650 (80.1%) | 50 (1.2) | 3,319,830 (80.0%) | 480 (1.1) |
| 4 | 426,430 (17.1%) | 460 (7.4) | 102,970 (23.1%) | 150 (8.5) | 160,030 (19.8%) | 160 (5.9) | 73,140 (18.4%) | 70 (6.6) | 762,570 (18.4%) | 830 (7.1) |
| 5 | 45,640 (1.8%) | 40 (7.2) | 4,140 (0.9%) | - | 10,880 (1.3%) | 10 (9.4) | 5,980 (1.5%) | - | 66,650 (1.6%) | 60 (6.6) |
| **Time since previous COVID-19 vaccination** | | | | | | | | | | |
| <24 weeks | 204,840 (8.2%) | 230 (7.5) | 71,650 (16.1%) | 120 (8.8) | 82,170 (10.2%) | 90 (5.8) | 29,870 (7.5%) | 30 (5.4) | 388,530 (9.4%) | 460 (7.2) |
| ≥24 weeks | 2,293,400 (91.8%) | 530 (1.8) | 373,250 (83.9%) | 120 (2.1) | 725,960 (89.8%) | 170 (1.9) | 367,890 (92.5%) | 100 (2.1) | 3,760,500 (90.6%) | 920 (1.9) |
| **Sex** | | | | | | | | | | |
| Female | 1,335,530 (53.5%) | 380 (2.1) | 239,340 (53.8%) | 100 (2.9) | 431,820 (53.4%) | 120 (2.0) | 206,680 (52.0%) | 50 (2.0) | 2,213,360 (53.3%) | 640 (2.2) |
| Male | 1,162,720 (46.5%) | 380 (2.5) | 205,560 (46.2%) | 130 (4.1) | 376,310 (46.6%) | 150 (3.0) | 191,090 (48.0%) | 70 (2.9) | 1,935,680 (46.7%) | 730 (2.9) |
| **Age** | | | | | | | | | | |
| 18-49 years | 327,620 (13.1%) | 10 (0.4) | 50,970 (11.5%) | - | 110,800 (13.7%) | - | 48,280 (12.1%) | - | 537,670 (13.0%) | 20 (0.3) |
| 50-64 years | 1,233,380 (49.4%) | 80 (0.6) | 163,540 (36.8%) | 10 (0.4) | 344,730 (42.7%) | 30 (0.9) | 166,110 (41.8%) | 10 (0.8) | 1,907,760 (46.0%) | 130 (0.6) |
| 65-79 years | 713,500 (28.6%) | 250 (2.3) | 168,350 (37.8%) | 80 (2.9) | 291,950 (36.1%) | 120 (2.5) | 147,080 (37.0%) | 50 (2.2) | 1,320,880 (31.8%) | 500 (2.4) |
| ≥80 years | 223,740 (9.0%) | 410 (12.6) | 62,040 (13.9%) | 140 (12.9) | 60,660 (7.5%) | 100 (9.9) | 36,300 (9.1%) | 60 (11.2) | 382,740 (9.2%) | 720 (12.2) |
| **Ethnicity** | | | | | | | | | | |
| White | 2,172,000 (86.9%) | 680 (2.4) | - | - | 579,440 (71.7%) | 230 (3.0) | 384,730 (96.7%) | 120 (2.5) | 3,136,170 (84.7%) | 1,030 (2.5) |
| Asian | 94,090 (3.8%) | 10 (1.3) | - | - | 4,560 (0.6%) | 0 (0.0) | 6,220 (1.6%) | 0 (0.0) | 104,870 (2.8%) | 10 (0.8) |
| Black | 30,270 (1.2%) | - | - | - | 680 (0.1%) | 0 (0.0) | 1,010 (0.3%) | 0 (0.0) | 31,960 (0.9%) | 10 (2.7) |
| Mixed | 18,300 (0.7%) | - | - | - | 1,070 (0.1%) | 0 (0.0) | 1,460 (0.4%) | 0 (0.0) | 20,820 (0.6%) | 0 (0.0) |
| Other | 14,010 (0.6%) | - | - | - | 1,100 (0.1%) | 0 (0.0) | 1,380 (0.3%) | 0 (0.0) | 16,490 (0.4%) | 0 (0.0) |
| Unknown | 169,580 (6.8%) | 50 (2.5) | - | - | 221,280 (27.4%) | 30 (1.0) | 2,970 (0.7%) | - | 393,830 (10.6%) | 80 (1.6) |
| **BMI** | | | | | | | | | | |
| <18.5 | 42,180 (1.7%) | 40 (7.1) | - | - | 6,270 (0.8%) | - | 6,320 (1.6%) | - | 54,770 (1.5%) | 50 (7.1) |
| 18.5-24.9 | 791,660 (31.7%) | 280 (2.8) | - | - | 101,210 (12.5%) | 60 (4.3) | 96,860 (24.4%) | 40 (2.8) | 989,730 (26.7%) | 380 (2.9) |
| 25.0-29.9 | 893,320 (35.8%) | 250 (2.2) | - | - | 467,460 (57.8%) | 110 (1.8) | 134,050 (33.7%) | 50 (2.7) | 1,494,830 (40.4%) | 410 (2.1) |
| 30.0-39.9 | 651,730 (26.1%) | 160 (1.8) | - | - | 189,300 (23.4%) | 70 (2.9) | 136,570 (34.3%) | 30 (1.5) | 977,600 (26.4%) | 260 (2.0) |
| ≥40.0 | 119,350 (4.8%) | 20 (1.5) | - | - | 43,900 (5.4%) | 10 (2.1) | 23,970 (6.0%) | 10 (3.8) | 187,210 (5.1%) | 40 (1.7) |
| **Number of QCovid risk groups** | | | | | | | | | | |
| 0 | 995,800 (39.9%) | 70 (0.5) | - | - | 412,440 (51.0%) | 40 (0.8) | 184,200 (46.3%) | 20 (0.9) | 1,592,440 (43.0%) | 130 (0.7) |
| 1 | 673,270 (26.9%) | 120 (1.4) | - | - | 257,850 (31.9%) | 80 (2.2) | 117,800 (29.6%) | 20 (1.5) | 1,048,920 (28.3%) | 220 (1.6) |
| 2 | 378,030 (15.1%) | 140 (2.7) | - | - | 90,210 (11.2%) | 60 (4.7) | 54,820 (13.8%) | 20 (2.6) | 523,060 (14.1%) | 220 (3.1) |
| 3 | 222,330 (8.9%) | 130 (4.3) | - | - | 31,510 (3.9%) | 40 (8.8) | 23,770 (6.0%) | 30 (7.7) | 277,610 (7.5%) | 200 (5.2) |
| 4 | 119,790 (4.8%) | 110 (6.8) | - | - | 11,170 (1.4%) | 30 (15.2) | 10,400 (2.6%) | 20 (10.4) | 141,360 (3.8%) | 160 (8.1) |
| ≥5 | 109,030 (4.4%) | 190 (12.4) | - | - | 4,950 (0.6%) | 10 (15.7) | 6,790 (1.7%) | 20 (22.6) | 120,780 (3.3%) | 220 (13.1) |
| **Number of BNF risk groups** | | | | | | | | | | |
| 0 | - | - | 93,930 (21.1%) | - | - | - | - | - | 93,930 (21.1%) | 0 (0.0) |
| 1 | - | - | 84,580 (19.0%) | 10 (1.1) | - | - | - | - | 84,580 (19.0%) | 10 (0.8) |
| 2 | - | - | 81,400 (18.3%) | 20 (1.3) | - | - | - | - | 81,400 (18.3%) | 20 (1.6) |
| 3 | - | - | 66,770 (15.0%) | 40 (3.9) | - | - | - | - | 66,770 (15.0%) | 40 (3.9) |
| 4 | - | - | 49,180 (11.1%) | 40 (5.4) | - | - | - | - | 49,180 (11.1%) | 40 (5.2) |
| 5 | - | - | 32,800 (7.4%) | 40 (7.6) | - | - | - | - | 32,800 (7.4%) | 40 (7.8) |
| ≥6 | - | - | 36,240 (8.1%) | 70 (12.8) | - | - | - | - | 36,240 (8.1%) | 70 (12.3) |
| **Household size** | | | | | | | | | | |
| 1 person | - | - | 75,460 (17.0%) | 60 (5.5) | 265,170 (32.8%) | 120 (3.3) | 70,730 (17.8%) | 40 (4.2) | 411,360 (24.9%) | 220 (3.8) |
| 2 people | - | - | 149,760 (33.7%) | 90 (4.0) | 323,760 (40.1%) | 100 (2.2) | 161,920 (40.7%) | 50 (2.3) | 635,440 (38.5%) | 240 (2.7) |
| 3-5 people | - | - | 193,110 (43.4%) | 60 (2.1) | 209,090 (25.9%) | 40 (1.6) | 149,140 (37.5%) | 20 (1.3) | 551,340 (33.4%) | 120 (1.7) |
| 6-10 people | - | - | 22,040 (5.0%) | - | 8,910 (1.1%) | - | 12,570 (3.2%) | - | 43,520 (2.6%) | 0 (0.0) |
| ≥11 people | - | - | 4,530 (1.0%) | - | 1,210 (0.1%) | - | 3,410 (0.9%) | - | 9,150 (0.6%) | 10 (7.5) |
| **Socioeconomic deprivation quintile** | | | | | | | | | | |
| 5th (Least) | 702,540 (28.1%) | 210 (2.2) | 111,870 (25.1%) | 50 (2.9) | 133,090 (16.5%) | 40 (2.2) | 100,710 (25.3%) | 30 (2.3) | 1,048,210 (25.3%) | 330 (2.3) |
| 4th | 604,750 (24.2%) | 170 (2.1) | 98,580 (22.2%) | 60 (3.8) | 196,850 (24.4%) | 50 (1.8) | 89,940 (22.6%) | 30 (2.2) | 990,110 (23.9%) | 300 (2.3) |
| 3rd | 512,010 (20.5%) | 150 (2.3) | 87,290 (19.6%) | 40 (2.7) | 203,840 (25.2%) | 50 (2.0) | 80,150 (20.1%) | 30 (2.5) | 883,290 (21.3%) | 280 (2.4) |
| 2nd | 404,800 (16.2%) | 120 (2.4) | 82,160 (18.5%) | 40 (3.5) | 155,420 (19.2%) | 60 (2.9) | 72,360 (18.2%) | 20 (2.5) | 714,740 (17.2%) | 240 (2.5) |
| 1st (Most) | 274,140 (11.0%) | 110 (3.2) | 65,000 (14.6%) | 50 (4.6) | 118,940 (14.7%) | 60 (4.0) | 54,610 (13.7%) | 20 (2.9) | 512,690 (12.4%) | 240 (3.6) |
| **Rural/urban area classification** | | | | | | | | | | |
| Urban | 1,873,140 (75.0%) | 610 (2.5) | 285,890 (64.3%) | 160 (3.6) | 655,300 (81.1%) | 230 (2.6) | 262,310 (65.9%) | 80 (2.5) | 3,076,630 (74.2%) | 1,080 (2.7) |
| Rural | 625,110 (25.0%) | 150 (1.7) | 159,000 (35.7%) | 70 (3.1) | 152,830 (18.9%) | 30 (1.7) | 135,460 (34.1%) | 40 (2.3) | 1,072,400 (25.8%) | 290 (2.0) |
| **Spikevax** | | | | | | | | | | |
| **Total** | 1,849,980 (100.0%) | 1,230 (3.1) | - | - | 1,021,560 (100.0%) | 560 (3.2) | 420,920 (100.0%) | 330 (3.9) | 3,292,460 (100.0%) | 2,120 (3.2) |
| **Number of previous COVID-19 vaccinations** | | | | | | | | | | |
| 3 | 1,107,250 (59.9%) | 260 (1.1) | - | - | 712,520 (69.7%) | 120 (1.1) | 266,210 (63.2%) | 60 (1.3) | 2,085,980 (63.4%) | 440 (1.1) |
| 4 | 695,460 (37.6%) | 930 (5.9) | - | - | 293,880 (28.8%) | 420 (7.0) | 139,290 (33.1%) | 230 (7.2) | 1,128,630 (34.3%) | 1,580 (6.4) |
| 5 | 47,270 (2.6%) | 40 (4.3) | - | - | 15,160 (1.5%) | 20 (6.2) | 15,430 (3.7%) | 40 (10.3) | 77,850 (2.4%) | 100 (6.0) |
| **Time since previous COVID-19 vaccination** | | | | | | | | | | |
| <24 weeks | 445,000 (24.1%) | 660 (6.5) | - | - | 210,470 (20.6%) | 320 (7.2) | 122,250 (29.0%) | 230 (7.6) | 777,720 (23.6%) | 1,210 (6.8) |
| ≥24 weeks | 1,404,980 (75.9%) | 570 (2.0) | - | - | 811,090 (79.4%) | 240 (1.8) | 298,670 (71.0%) | 100 (1.9) | 2,514,740 (76.4%) | 910 (1.9) |
| **Sex** | | | | | | | | | | |
| Female | 1,008,010 (54.5%) | 600 (2.8) | - | - | 578,800 (56.7%) | 290 (2.9) | 235,310 (55.9%) | 160 (3.4) | 1,822,120 (55.3%) | 1,050 (2.9) |
| Male | 841,960 (45.5%) | 630 (3.5) | - | - | 442,760 (43.3%) | 270 (3.7) | 185,610 (44.1%) | 170 (4.5) | 1,470,340 (44.7%) | 1,070 (3.7) |
| **Age** | | | | | | | | | | |
| 18-49 years | 171,970 (9.3%) | 20 (0.4) | - | - | 157,250 (15.4%) | 10 (0.5) | 52,410 (12.5%) | 10 (1.1) | 381,620 (11.6%) | 40 (0.6) |
| 50-64 years | 386,990 (20.9%) | 60 (0.7) | - | - | 340,380 (33.3%) | 50 (1.0) | 129,180 (30.7%) | 30 (1.5) | 856,560 (26.0%) | 130 (0.9) |
| 65-79 years | 898,940 (48.6%) | 350 (1.8) | - | - | 372,090 (36.4%) | 200 (2.7) | 163,080 (38.7%) | 100 (3.0) | 1,434,110 (43.6%) | 650 (2.1) |
| ≥80 years | 392,070 (21.2%) | 810 (9.1) | - | - | 151,840 (14.9%) | 310 (9.4) | 76,250 (18.1%) | 180 (10.2) | 620,160 (18.8%) | 1,290 (9.3) |
| **Ethnicity** | | | | | | | | | | |
| White | 1,624,620 (87.8%) | 1,110 (3.2) | - | - | 755,220 (73.9%) | 490 (3.7) | 407,830 (96.9%) | 330 (4.0) | 2,787,670 (84.7%) | 1,930 (3.4) |
| Asian | 76,920 (4.2%) | 30 (1.8) | - | - | 16,430 (1.6%) | 10 (4.7) | 6,660 (1.6%) | - | 100,010 (3.0%) | 40 (2.0) |
| Black | 21,140 (1.1%) | - | - | - | 2,460 (0.2%) | 0 (0.0) | 1,080 (0.3%) | 0 (0.0) | 24,690 (0.7%) | 10 (2.0) |
| Mixed | 12,850 (0.7%) | - | - | - | 2,740 (0.3%) | - | 1,480 (0.4%) | - | 17,070 (0.5%) | 10 (3.0) |
| Other | 10,070 (0.5%) | - | - | - | 2,520 (0.2%) | 0 (0.0) | 1,200 (0.3%) | 0 (0.0) | 13,780 (0.4%) | 0 (0.0) |
| Unknown | 104,380 (5.6%) | 70 (3.3) | - | - | 242,190 (23.7%) | 60 (1.6) | 2,680 (0.6%) | 0 (0.0) | 349,240 (10.6%) | 130 (2.1) |
| **BMI** | | | | | | | | | | |
| <18.5 | 33,720 (1.8%) | 90 (12.0) | - | - | 11,120 (1.1%) | 30 (15.9) | 7,900 (1.9%) | 20 (11.4) | 52,740 (1.6%) | 140 (12.8) |
| 18.5-24.9 | 596,990 (32.3%) | 480 (3.8) | - | - | 150,460 (14.7%) | 150 (5.3) | 108,710 (25.8%) | 100 (4.5) | 856,160 (26.0%) | 730 (4.1) |
| 25.0-29.9 | 678,710 (36.7%) | 380 (2.6) | - | - | 601,850 (58.9%) | 240 (2.4) | 143,150 (34.0%) | 110 (3.9) | 1,423,710 (43.2%) | 740 (2.7) |
| 30.0-39.9 | 464,240 (25.1%) | 250 (2.5) | - | - | 214,740 (21.0%) | 130 (3.4) | 138,960 (33.0%) | 80 (3.0) | 817,930 (24.8%) | 450 (2.8) |
| ≥40.0 | 76,320 (4.1%) | 30 (2.0) | - | - | 43,390 (4.2%) | 20 (2.5) | 22,200 (5.3%) | 10 (3.4) | 141,900 (4.3%) | 60 (2.2) |
| **Number of QCovid risk groups** | | | | | | | | | | |
| 0 | 572,160 (30.9%) | 90 (0.7) | - | - | 505,420 (49.5%) | 70 (0.8) | 170,020 (40.4%) | 40 (1.1) | 1,247,610 (37.9%) | 200 (0.8) |
| 1 | 495,500 (26.8%) | 150 (1.4) | - | - | 314,380 (30.8%) | 140 (2.7) | 123,420 (29.3%) | 50 (2.0) | 933,300 (28.3%) | 340 (1.8) |
| 2 | 326,850 (17.7%) | 220 (3.1) | - | - | 121,970 (11.9%) | 140 (6.3) | 65,910 (15.7%) | 80 (6.1) | 514,720 (15.6%) | 440 (4.1) |
| 3 | 209,200 (11.3%) | 230 (5.0) | - | - | 49,670 (4.9%) | 110 (11.1) | 33,240 (7.9%) | 60 (8.0) | 292,110 (8.9%) | 390 (6.3) |
| 4 | 122,810 (6.6%) | 190 (7.2) | - | - | 19,780 (1.9%) | 60 (14.0) | 15,950 (3.8%) | 40 (12.3) | 158,530 (4.8%) | 290 (8.5) |
| ≥5 | 123,450 (6.7%) | 360 (13.4) | - | - | 10,350 (1.0%) | 40 (21.2) | 12,380 (2.9%) | 60 (22.8) | 146,180 (4.4%) | 470 (14.8) |
| **Household size** | | | | | | | | | | |
| 1 person | - | - | - | - | 367,530 (36.0%) | 250 (3.9) | 84,850 (20.2%) | 90 (5.2) | 452,380 (31.4%) | 340 (4.1) |
| 2 people | - | - | - | - | 373,740 (36.6%) | 210 (3.1) | 178,440 (42.4%) | 130 (3.5) | 552,180 (38.3%) | 340 (3.3) |
| 3-5 people | - | - | - | - | 258,110 (25.3%) | 60 (1.5) | 136,670 (32.5%) | 60 (2.4) | 394,780 (27.4%) | 120 (1.8) |
| 6-10 people | - | - | - | - | 13,160 (1.3%) | - | 11,730 (2.8%) | - | 24,890 (1.7%) | 10 (2.3) |
| ≥11 people | - | - | - | - | 9,030 (0.9%) | 30 (15.0) | 9,240 (2.2%) | 40 (16.4) | 18,270 (1.3%) | 70 (16.1) |
| **Socioeconomic deprivation quintile** | | | | | | | | | | |
| 5th (Least) | 498,320 (26.9%) | 310 (2.9) | - | - | 286,920 (28.1%) | 100 (2.2) | 106,830 (25.4%) | 80 (3.7) | 892,060 (27.1%) | 490 (2.8) |
| 4th | 436,320 (23.6%) | 290 (3.1) | - | - | 210,900 (20.6%) | 100 (2.6) | 82,580 (19.6%) | 50 (3.2) | 729,800 (22.2%) | 440 (3.0) |
| 3rd | 382,450 (20.7%) | 230 (2.8) | - | - | 178,210 (17.4%) | 100 (3.1) | 80,540 (19.1%) | 60 (3.8) | 641,200 (19.5%) | 380 (2.9) |
| 2nd | 307,270 (16.6%) | 220 (3.3) | - | - | 183,570 (18.0%) | 140 (4.3) | 80,950 (19.2%) | 70 (4.1) | 571,780 (17.4%) | 430 (3.8) |
| 1st (Most) | 225,620 (12.2%) | 190 (3.9) | - | - | 161,970 (15.9%) | 130 (4.6) | 70,020 (16.6%) | 70 (4.7) | 457,620 (13.9%) | 380 (4.3) |
| **Rural/urban area classification** | | | | | | | | | | |
| Urban | 1,431,850 (77.4%) | 1,000 (3.3) | - | - | 878,740 (86.0%) | 500 (3.3) | 300,680 (71.4%) | 260 (4.2) | 2,611,270 (79.3%) | 1,760 (3.4) |
| Rural | 418,120 (22.6%) | 230 (2.6) | - | - | 142,820 (14.0%) | 60 (2.6) | 120,240 (28.6%) | 70 (3.0) | 681,180 (20.7%) | 360 (2.6) |

## **Table S8:** Nation and meta, adjusted hazard ratios with 95% confidence intervals, for vaccination, socio-demographics and clinical factors associated with severe COVID-19 outcomes.

|  | **England** | **NI** | **Scotland** | **Wales** | **Meta-analysis** | **Q-test** |
| --- | --- | --- | --- | --- | --- | --- |
| **Overall** |  |  |  |  |  |  |
| **Time post Autumn 2022 vaccination** | | | | | |  |
| 2-3 weeks | 1.00 | 1.00 | 1.00 | 1.00 | 1.00 |  |
| 4-5 weeks | 1.04 (0.83, 1.30) | 0.81 (0.49, 1.35) | 0.94 (0.71, 1.25) | 1.44 (0.95, 2.19) | 1.03 (0.88, 1.21) | 3.78 (p=0.2860) |
| 6-7 weeks | 0.92 (0.72, 1.17) | 0.67 (0.38, 1.17) | 1.07 (0.79, 1.44) | 1.24 (0.79, 1.95) | 0.97 (0.82, 1.15) | 3.36 (p=0.3388) |
| 8-9 weeks | 0.98 (0.76, 1.25) | 0.74 (0.41, 1.31) | 0.90 (0.65, 1.26) | 1.62 (1.02, 2.57) | 1.00 (0.84, 1.19) | 5.61 (p=0.1319) |
| 10-11 weeks | 0.88 (0.68, 1.13) | 0.54 (0.28, 1.03) | 0.78 (0.54, 1.12) | 1.49 (0.91, 2.45) | 0.88 (0.73, 1.06) | 7.01 (p=0.0715) |
| ≥12 weeks | 0.85 (0.64, 1.12) | 0.71 (0.33, 1.52) | 0.97 (0.66, 1.44) | 1.42 (0.83, 2.42) | 0.93 (0.76, 1.14) | 3.36 (p=0.3393) |
| **Time since previous COVID-19 vaccination** | | | | | |  |
| <24 weeks | 1.45 (1.32, 1.60) | 1.44 (1.06, 1.97) | 1.60 (1.36, 1.88) | 1.52 (1.19, 1.93) | 1.49 (1.38, 1.60) | 1.09 (p=0.7793) |
| ≥24 weeks | 1.00 | 1.00 | 1.00 | 1.00 | 1.00 |  |
| **Sex** | | | | | |  |
| Female | 1.00 | 1.00 | 1.00 | 1.00 | 1.00 |  |
| Male | 1.39 (1.27, 1.52) | 1.72 (1.32, 2.23) | 1.42 (1.23, 1.63) | 1.35 (1.12, 1.64) | 1.41 (1.32, 1.51) | 2.46 (p=0.4817) |
| **Age** | | | | | |  |
| 18-49 years | 1.00 | 1.00 | 1.00 | 1.00 | 1.00 |  |
| 50-64 years | 1.73 (1.16, 2.58) | 0.45 (0.17, 1.22) | 1.88 (1.11, 3.19) | 1.23 (0.67, 2.24) | 1.49 (1.14, 1.96) | 7.22 (p=0.0653) |
| 65-79 years | 4.96 (3.40, 7.21) | 2.16 (0.92, 5.05) | 4.52 (2.72, 7.50) | 2.03 (1.14, 3.59) | 3.76 (2.92, 4.85) | 8.71 (p=0.0334) |
| ≥80 years | 16.20 (11.13, 23.57) | 6.76 (2.86, 16.02) | 9.80 (5.84, 16.44) | 4.70 (2.61, 8.46) | 10.43 (8.06, 13.50) | 13.38 (p=0.0039) |
| **Ethnicity** | | | | | |  |
| White | 1.00 | - | - | - | 1.00 |  |
| Asian | 0.63 (0.46, 0.85) | - | - | - | 0.63 (0.46, 0.85) |  |
| Black | 0.61 (0.36, 1.04) | - | - | - | 0.61 (0.36, 1.04) |  |
| Mixed | 0.84 (0.44, 1.62) | - | - | - | 0.84 (0.44, 1.62) |  |
| Other | 0.62 (0.26, 1.49) | - | - | - | 0.62 (0.26, 1.49) |  |
| Unknown | 1.16 (0.96, 1.39) | - | - | - | 1.16 (0.96, 1.39) |  |
| **BMI** | | | | | |  |
| <18.5 | 3.03 (2.50, 3.69) | - | 3.30 (2.36, 4.61) | 2.05 (1.33, 3.18) | 2.94 (2.51, 3.44) | 3.16 (p=0.2063) |
| 18.5-24.9 | 1.40 (1.26, 1.55) | - | 1.49 (1.25, 1.78) | 1.05 (0.83, 1.32) | 1.36 (1.25, 1.48) | 6.18 (p=0.0456) |
| 25.0-29.9 | 1.00 | - | 1.00 | 1.00 | 1.00 |  |
| 30.0-39.9 | 0.95 (0.84, 1.08) | - | 1.06 (0.89, 1.27) | 0.77 (0.60, 0.99) | 0.95 (0.87, 1.05) | 4.31 (p=0.1159) |
| ≥40.0 | 1.12 (0.85, 1.48) | - | 1.16 (0.79, 1.72) | 1.45 (0.94, 2.24) | 1.20 (0.98, 1.46) | 1.03 (p=0.5976) |
| **Number of QCovid risk groups** | | | | | |  |
| 0 | 1.00 | - | 1.00 | 1.00 | 1.00 |  |
| 1 | 1.88 (1.54, 2.29) | - | 2.34 (1.86, 2.94) | 1.50 (1.05, 2.13) | 1.96 (1.71, 2.25) | 4.66 (p=0.0974) |
| 2 | 3.19 (2.64, 3.87) | - | 3.80 (2.99, 4.83) | 3.36 (2.41, 4.69) | 3.41 (2.97, 3.91) | 1.25 (p=0.5361) |
| 3 | 4.59 (3.79, 5.57) | - | 5.63 (4.35, 7.29) | 4.69 (3.30, 6.67) | 4.90 (4.25, 5.64) | 1.61 (p=0.4474) |
| 4 | 6.13 (5.03, 7.47) | - | 6.84 (5.06, 9.25) | 6.20 (4.23, 9.08) | 6.31 (5.42, 7.35) | 0.37 (p=0.8305) |
| ≥5 | 9.39 (7.80, 11.30) | - | 8.55 (6.10, 11.98) | 10.86 (7.58, 15.56) | 9.45 (8.15, 10.96) | 0.92 (p=0.6302) |
| **Number of BNF risk groups** | | | | | |  |
| 0 | - | 1.00 | - | - | 1.00 |  |
| 1 | - | 1.58 (0.64, 3.88) | - | - | 1.58 (0.64, 3.88) |  |
| 2 | - | 1.38 (0.56, 3.40) | - | - | 1.38 (0.56, 3.40) |  |
| 3 | - | 3.69 (1.63, 8.34) | - | - | 3.69 (1.63, 8.34) |  |
| 4 | - | 4.53 (2.00, 10.25) | - | - | 4.53 (2.00, 10.25) |  |
| 5 | - | 6.08 (2.67, 13.82) | - | - | 6.08 (2.67, 13.82) |  |
| ≥6 | - | 9.14 (4.11, 20.32) | - | - | 9.14 (4.11, 20.32) |  |
| **Household size** | | | | | |  |
| 1 person | - | 1.13 (0.82, 1.55) | 1.04 (0.89, 1.22) | 1.13 (0.90, 1.43) | 1.08 (0.96, 1.21) | 0.45 (p=0.7997) |
| 2 people | - | 1.00 | 1.00 | 1.00 | 1.00 |  |
| 3-5 people | - | 1.02 (0.73, 1.42) | 1.07 (0.85, 1.36) | 1.08 (0.82, 1.41) | 1.06 (0.91, 1.24) | 0.07 (p=0.9669) |
| 6-10 people | - | 0.73 (0.29, 1.81) | 1.62 (0.89, 2.97) | 1.34 (0.73, 2.48) | 1.30 (0.88, 1.92) | 2.08 (p=0.3529) |
| ≥11 people | - | 1.11 (0.55, 2.24) | 1.42 (0.99, 2.04) | 1.87 (1.32, 2.66) | 1.56 (1.23, 1.98) | 2.17 (p=0.3374) |
| **Socioeconomic deprivation quintile** | | | | | |  |
| 5th (Least) | 1.00 | 1.00 | 1.00 | 1.00 | 1.00 |  |
| 4th | 1.05 (0.92, 1.19) | 1.27 (0.85, 1.90) | 1.04 (0.82, 1.32) | 0.88 (0.65, 1.19) | 1.04 (0.94, 1.15) | 2.18 (p=0.5364) |
| 3rd | 1.00 (0.87, 1.14) | 0.81 (0.51, 1.28) | 1.14 (0.90, 1.44) | 0.99 (0.74, 1.33) | 1.01 (0.91, 1.13) | 1.97 (p=0.5793) |
| 2nd | 1.09 (0.94, 1.25) | 1.11 (0.72, 1.71) | 1.47 (1.18, 1.83) | 0.99 (0.74, 1.33) | 1.15 (1.04, 1.28) | 6.37 (p=0.0951) |
| 1st (Most) | 1.36 (1.17, 1.58) | 1.35 (0.86, 2.09) | 1.57 (1.25, 1.97) | 1.04 (0.78, 1.40) | 1.35 (1.21, 1.51) | 4.67 (p=0.1975) |
| **Rural/urban area classification** | | | | | |  |
| Urban | 1.00 | 1.00 | 1.00 | 1.00 | 1.00 |  |
| Rural | 0.78 (0.70, 0.88) | 0.84 (0.62, 1.14) | 0.89 (0.71, 1.12) | 0.88 (0.69, 1.12) | 0.82 (0.75, 0.90) | 1.48 (p=0.6866) |
| **Comirnaty** |  |  |  |  |  |  |
| **Time post Autumn 2022 vaccination** | | | | | |  |
| 2-3 weeks | 1.00 | 1.00 | 1.00 | 1.00 | 1.00 |  |
| 4-5 weeks | 1.00 (0.74, 1.34) | 0.84 (0.50, 1.42) | 0.91 (0.57, 1.43) | 1.31 (0.67, 2.53) | 0.98 (0.79, 1.21) | 1.18 (p=0.7586) |
| 6-7 weeks | 0.90 (0.67, 1.22) | 0.69 (0.39, 1.21) | 1.12 (0.70, 1.81) | 1.46 (0.73, 2.89) | 0.95 (0.77, 1.19) | 3.30 (p=0.3474) |
| 8-9 weeks | 0.96 (0.72, 1.29) | 0.76 (0.42, 1.36) | 0.91 (0.53, 1.54) | 1.91 (0.95, 3.84) | 0.99 (0.79, 1.23) | 4.36 (p=0.2254) |
| 10-11 weeks | 0.80 (0.59, 1.09) | 0.55 (0.28, 1.05) | 0.80 (0.44, 1.44) | 1.46 (0.66, 3.23) | 0.80 (0.63, 1.02) | 3.55 (p=0.3141) |
| ≥12 weeks | 0.66 (0.43, 1.02) | 0.77 (0.36, 1.66) | 1.00 (0.49, 2.05) | 1.02 (0.26, 3.91) | 0.76 (0.55, 1.05) | 1.15 (p=0.7657) |
| **Time since previous COVID-19 vaccination** | | | | | |  |
| <24 weeks | 1.40 (1.19, 1.65) | 1.42 (1.04, 1.95) | 1.57 (1.16, 2.11) | 0.98 (0.61, 1.57) | 1.40 (1.23, 1.58) | 2.76 (p=0.4309) |
| ≥24 weeks | 1.00 | 1.00 | 1.00 | 1.00 | 1.00 |  |
| **Sex** | | | | | |  |
| Female | 1.00 | 1.00 | 1.00 | 1.00 | 1.00 |  |
| Male | 1.39 (1.20, 1.60) | 1.67 (1.28, 2.17) | 1.60 (1.25, 2.06) | 1.57 (1.09, 2.27) | 1.49 (1.33, 1.66) | 2.05 (p=0.5629) |
| **Age** | | | | | |  |
| 18-49 years | 1.00 | 1.00 | 1.00 | 1.00 | 1.00 |  |
| 50-64 years | 1.87 (1.06, 3.31) | 0.41 (0.15, 1.14) | 1.80 (0.75, 4.33) | 1.43 (0.41, 5.00) | 1.41 (0.94, 2.12) | 6.89 (p=0.0754) |
| 65-79 years | 6.64 (3.86, 11.42) | 2.22 (0.95, 5.20) | 5.23 (2.21, 12.39) | 3.30 (0.99, 11.02) | 4.73 (3.22, 6.93) | 4.94 (p=0.1765) |
| ≥80 years | 22.88 (13.32, 39.32) | 6.80 (2.87, 16.12) | 13.52 (5.59, 32.70) | 11.20 (3.25, 38.65) | 15.13 (10.28, 22.27) | 5.83 (p=0.1202) |
| **Ethnicity** | | | | | |  |
| White | 1.00 | - | - | - | 1.00 |  |
| Asian | 0.52 (0.30, 0.90) | - | - | - | 0.52 (0.30, 0.90) | 0.00 (p=1.0000) |
| Black | 0.67 (0.31, 1.42) | - | - | - | 0.67 (0.31, 1.42) | 0.00 (p=1.0000) |
| Mixed | 0.73 (0.23, 2.27) | - | - | - | 0.73 (0.23, 2.27) | 0.00 (p=1.0000) |
| Other | 0.30 (0.04, 2.11) | - | - | - | 0.30 (0.04, 2.11) | 0.00 (p=1.0000) |
| Unknown | 1.21 (0.91, 1.60) | - | - | - | 1.21 (0.91, 1.60) | 0.00 (p=1.0000) |
| **BMI** | | | | | |  |
| <18.5 | 2.33 (1.65, 3.30) | - | 3.20 (1.55, 6.62) | 1.59 (0.62, 4.04) | 2.37 (1.76, 3.18) | 1.36 (p=0.5055) |
| 18.5-24.9 | 1.31 (1.10, 1.55) | - | 1.72 (1.25, 2.37) | 0.96 (0.62, 1.49) | 1.34 (1.16, 1.54) | 4.61 (p=0.1000) |
| 25.0-29.9 | 1.00 | - | 1.00 | 1.00 | 1.00 |  |
| 30.0-39.9 | 0.89 (0.72, 1.08) | - | 1.20 (0.89, 1.63) | 0.59 (0.36, 0.96) | 0.92 (0.79, 1.08) | 6.36 (p=0.0416) |
| ≥40.0 | 1.03 (0.68, 1.58) | - | 1.26 (0.67, 2.37) | 2.01 (1.02, 3.97) | 1.25 (0.91, 1.71) | 2.68 (p=0.2621) |
| **Number of QCovid risk groups** | | | | | |  |
| 0 | 1.00 | - | 1.00 | 1.00 | 1.00 |  |
| 1 | 2.18 (1.61, 2.93) | - | 2.16 (1.48, 3.16) | 1.34 (0.74, 2.45) | 2.04 (1.64, 2.54) | 2.13 (p=0.3451) |
| 2 | 3.29 (2.44, 4.43) | - | 3.33 (2.22, 4.98) | 1.92 (1.02, 3.62) | 3.08 (2.46, 3.86) | 2.47 (p=0.2914) |
| 3 | 4.53 (3.35, 6.14) | - | 4.98 (3.18, 7.79) | 4.79 (2.62, 8.74) | 4.69 (3.72, 5.91) | 0.12 (p=0.9412) |
| 4 | 6.12 (4.48, 8.37) | - | 7.40 (4.42, 12.37) | 5.47 (2.74, 10.91) | 6.30 (4.91, 8.09) | 0.57 (p=0.7528) |
| ≥5 | 8.54 (6.37, 11.47) | - | 6.87 (3.53, 13.36) | 10.12 (5.29, 19.34) | 8.50 (6.63, 10.89) | 0.67 (p=0.7142) |
| **Number of BNF risk groups** | | | | | |  |
| 0 | - | 1.00 | - | - | 1.00 |  |
| 1 | - | 1.48 (0.59, 3.67) | - | - | 1.48 (0.59, 3.67) | 0.00 (p=1.0000) |
| 2 | - | 1.39 (0.57, 3.42) | - | - | 1.39 (0.57, 3.42) | 0.00 (p=1.0000) |
| 3 | - | 3.61 (1.59, 8.18) | - | - | 3.61 (1.59, 8.18) | 0.00 (p=1.0000) |
| 4 | - | 4.54 (2.00, 10.29) | - | - | 4.54 (2.00, 10.29) | 0.00 (p=1.0000) |
| 5 | - | 5.80 (2.54, 13.23) | - | - | 5.80 (2.54, 13.23) | 0.00 (p=1.0000) |
| ≥6 | - | 9.08 (4.08, 20.19) | - | - | 9.08 (4.08, 20.19) | 0.00 (p=1.0000) |
| **Household size** | | | | | |  |
| 1 person | - | 1.10 (0.79, 1.52) | 1.22 (0.93, 1.60) | 1.31 (0.85, 2.03) | 1.19 (0.99, 1.44) | 0.48 (p=0.7872) |
| 2 people | - | 1.00 | 1.00 | 1.00 | 1.00 |  |
| 3-5 people | - | 1.05 (0.75, 1.47) | 1.39 (0.94, 2.07) | 1.00 (0.60, 1.68) | 1.14 (0.91, 1.44) | 1.46 (p=0.4820) |
| 6-10 people | - | 0.76 (0.31, 1.87) | 1.58 (0.39, 6.42) | 1.27 (0.39, 4.09) | 1.03 (0.54, 1.94) | 0.92 (p=0.6326) |
| ≥11 people | - | 1.12 (0.56, 2.25) | 3.00 (1.18, 7.60) | 2.96 (1.38, 6.33) | 1.98 (1.26, 3.11) | 4.39 (p=0.1111) |
| **Socioeconomic deprivation quintile** | | | | | |  |
| 5th (Least) | 1.00 | 1.00 | 1.00 | 1.00 | 1.00 |  |
| 4th | 0.94 (0.76, 1.15) | 1.27 (0.85, 1.90) | 0.77 (0.50, 1.19) | 0.86 (0.50, 1.48) | 0.95 (0.81, 1.12) | 3.03 (p=0.3864) |
| 3rd | 1.00 (0.81, 1.24) | 0.83 (0.52, 1.32) | 0.84 (0.55, 1.29) | 0.90 (0.52, 1.56) | 0.94 (0.80, 1.11) | 0.90 (p=0.8260) |
| 2nd | 0.95 (0.75, 1.19) | 1.04 (0.67, 1.62) | 1.11 (0.74, 1.68) | 0.92 (0.52, 1.62) | 0.99 (0.83, 1.17) | 0.59 (p=0.8987) |
| 1st (Most) | 1.26 (0.99, 1.61) | 1.38 (0.89, 2.15) | 1.46 (0.97, 2.20) | 1.02 (0.57, 1.84) | 1.29 (1.08, 1.55) | 1.07 (p=0.7843) |
| **Rural/urban area classification** | | | | | |  |
| Urban | 1.00 | 1.00 | 1.00 | 1.00 | 1.00 |  |
| Rural | 0.77 (0.63, 0.93) | 0.84 (0.62, 1.15) | 0.74 (0.50, 1.09) | 1.01 (0.65, 1.55) | 0.80 (0.69, 0.92) | 1.55 (p=0.6709) |
| **Spikevax** |  |  |  |  |  |  |
| **Time post Autumn 2022 vaccination** | | | | | |  |
| 2-3 weeks | 1.00 | - | 1.00 | 1.00 | 1.00 |  |
| 4-5 weeks | 1.10 (0.78, 1.56) | - | 0.96 (0.67, 1.38) | 1.54 (0.89, 2.66) | 1.11 (0.88, 1.39) | 2.02 (p=0.3644) |
| 6-7 weeks | 0.94 (0.60, 1.46) | - | 1.03 (0.69, 1.52) | 1.11 (0.60, 2.04) | 1.01 (0.77, 1.32) | 0.20 (p=0.9053) |
| 8-9 weeks | 1.07 (0.64, 1.77) | - | 0.90 (0.59, 1.38) | 1.46 (0.78, 2.71) | 1.06 (0.79, 1.41) | 1.55 (p=0.4602) |
| 10-11 weeks | 1.09 (0.64, 1.85) | - | 0.77 (0.49, 1.21) | 1.49 (0.78, 2.85) | 1.00 (0.73, 1.35) | 2.84 (p=0.2423) |
| ≥12 weeks | 1.08 (0.63, 1.86) | - | 0.98 (0.61, 1.58) | 1.41 (0.72, 2.76) | 1.10 (0.80, 1.50) | 0.75 (p=0.6874) |
| **Time since previous COVID-19 vaccination** | | | | | |  |
| <24 weeks | 1.48 (1.31, 1.67) | - | 1.64 (1.35, 1.99) | 1.88 (1.40, 2.52) | 1.56 (1.42, 1.72) | 2.48 (p=0.2888) |
| ≥24 weeks | 1.00 | - | 1.00 | 1.00 | 1.00 |  |
| **Sex** | | | | | |  |
| Female | 1.00 | - | 1.00 | 1.00 | 1.00 |  |
| Male | 1.40 (1.25, 1.57) | - | 1.34 (1.13, 1.59) | 1.28 (1.03, 1.61) | 1.37 (1.25, 1.49) | 0.49 (p=0.7827) |
| **Age** | | | | | |  |
| 18-49 years | 1.00 | - | 1.00 | 1.00 | 1.00 |  |
| 50-64 years | 1.67 (0.94, 2.96) | - | 2.01 (1.04, 3.89) | 1.21 (0.61, 2.39) | 1.61 (1.12, 2.32) | 1.13 (p=0.5681) |
| 65-79 years | 3.50 (2.08, 5.89) | - | 4.16 (2.22, 7.78) | 1.62 (0.85, 3.12) | 2.98 (2.12, 4.20) | 4.78 (p=0.0916) |
| ≥80 years | 11.08 (6.59, 18.63) | - | 8.31 (4.39, 15.73) | 3.23 (1.66, 6.31) | 7.34 (5.19, 10.36) | 8.34 (p=0.0154) |
| **Ethnicity** | | | | | |  |
| White | 1.00 | - | - | - | 1.00 |  |
| Asian | 0.69 (0.47, 1.01) | - | - | - | 0.69 (0.47, 1.01) | 0.00 (p=1.0000) |
| Black | 0.55 (0.26, 1.17) | - | - | - | 0.55 (0.26, 1.17) | 0.00 (p=1.0000) |
| Mixed | 0.90 (0.40, 2.02) | - | - | - | 0.90 (0.40, 2.02) | 0.00 (p=1.0000) |
| Other | 0.84 (0.32, 2.26) | - | - | - | 0.84 (0.32, 2.26) | 0.00 (p=1.0000) |
| Unknown | 1.12 (0.88, 1.43) | - | - | - | 1.12 (0.88, 1.43) | 0.00 (p=1.0000) |
| **BMI** | | | | | |  |
| <18.5 | 3.48 (2.75, 4.41) | - | 3.30 (2.26, 4.81) | 2.24 (1.36, 3.67) | 3.23 (2.68, 3.89) | 2.51 (p=0.2848) |
| 18.5-24.9 | 1.46 (1.27, 1.67) | - | 1.41 (1.14, 1.74) | 1.09 (0.83, 1.42) | 1.38 (1.24, 1.53) | 3.67 (p=0.1600) |
| 25.0-29.9 | 1.00 | - | 1.00 | 1.00 | 1.00 |  |
| 30.0-39.9 | 1.00 (0.85, 1.18) | - | 1.00 (0.80, 1.25) | 0.85 (0.64, 1.13) | 0.97 (0.86, 1.10) | 1.02 (p=0.6019) |
| ≥40.0 | 1.18 (0.82, 1.70) | - | 1.13 (0.68, 1.85) | 1.21 (0.68, 2.12) | 1.17 (0.90, 1.52) | 0.03 (p=0.9827) |
| **Number of QCovid risk groups** | | | | | |  |
| 0 | 1.00 | - | 1.00 | 1.00 | 1.00 |  |
| 1 | 1.66 (1.28, 2.16) | - | 2.43 (1.82, 3.25) | 1.57 (1.02, 2.43) | 1.90 (1.59, 2.27) | 4.52 (p=0.1044) |
| 2 | 3.07 (2.39, 3.95) | - | 4.06 (3.01, 5.47) | 4.02 (2.69, 6.02) | 3.55 (2.98, 4.22) | 2.40 (p=0.3009) |
| 3 | 4.53 (3.53, 5.81) | - | 5.96 (4.33, 8.20) | 4.64 (3.01, 7.16) | 4.96 (4.15, 5.93) | 1.88 (p=0.3900) |
| 4 | 5.99 (4.64, 7.75) | - | 6.67 (4.60, 9.68) | 6.48 (4.08, 10.29) | 6.25 (5.16, 7.57) | 0.24 (p=0.8852) |
| ≥5 | 9.66 (7.60, 12.27) | - | 9.32 (6.26, 13.89) | 11.16 (7.22, 17.27) | 9.84 (8.17, 11.85) | 0.42 (p=0.8120) |
| **Household size** | | | | | |  |
| 1 person | - | - | 0.96 (0.80, 1.16) | 1.07 (0.81, 1.40) | 0.99 (0.85, 1.16) | 0.40 (p=0.5263) |
| 2 people | - | - | 1.00 | 1.00 | 1.00 |  |
| 3-5 people | - | - | 0.93 (0.69, 1.26) | 1.11 (0.81, 1.53) | 1.01 (0.82, 1.26) | 0.63 (p=0.4283) |
| 6-10 people | - | - | 1.59 (0.81, 3.12) | 1.38 (0.67, 2.84) | 1.49 (0.91, 2.44) | 0.08 (p=0.7763) |
| ≥11 people | - | - | 1.25 (0.84, 1.86) | 1.76 (1.18, 2.62) | 1.49 (1.12, 1.97) | 1.41 (p=0.2358) |
| **Socioeconomic deprivation quintile** | | | | | |  |
| 5th (Least) | 1.00 | - | 1.00 | 1.00 | 1.00 |  |
| 4th | 1.12 (0.96, 1.32) | - | 1.17 (0.89, 1.56) | 0.89 (0.62, 1.27) | 1.10 (0.96, 1.25) | 1.64 (p=0.4406) |
| 3rd | 1.00 (0.84, 1.19) | - | 1.29 (0.97, 1.71) | 1.03 (0.73, 1.46) | 1.06 (0.93, 1.22) | 2.37 (p=0.3056) |
| 2nd | 1.18 (0.99, 1.41) | - | 1.63 (1.25, 2.11) | 1.02 (0.72, 1.43) | 1.26 (1.10, 1.44) | 5.70 (p=0.0578) |
| 1st (Most) | 1.42 (1.17, 1.71) | - | 1.58 (1.21, 2.08) | 1.06 (0.75, 1.48) | 1.39 (1.20, 1.60) | 3.43 (p=0.1800) |
| **Rural/urban area classification** | | | | | |  |
| Urban | 1.00 | - | 1.00 | 1.00 | 1.00 |  |
| Rural | 0.80 (0.69, 0.93) | - | 1.00 (0.76, 1.33) | 0.83 (0.62, 1.11) | 0.84 (0.74, 0.95) | 2.03 (p=0.3617) |

## **Table S9:** Test results for the proportional hazards assumption for the main overall Cox regression models.

|  | **England** | | | **Northern Ireland** | | | **Scotland** | | | **Wales** | | |
| --- | --- | --- | --- | --- | --- | --- | --- | --- | --- | --- | --- | --- |
|  | chisq | df | p | chisq | df | p | chisq | df | p | chisq | df | p |
| Global Test | 70.40 | 29 | <0.001 | 20.63 | 25 | 0.713 | 25.84 | 28 | 0.582 | 30.48 | 28 | 0.341 |
| **Time post Autumn 2022 vaccination** | | | | | | | | | | | | |
| 2-3 weeks | 0.13 | 1 | 0.716 | 8.03 | 1 | 0.005 | 0.06 | 1 | 0.806 | 1.03 | 1 | 0.311 |
| 4-5 weeks | 2.31 | 1 | 0.128 | 0.07 | 1 | 0.790 | 4.18 | 1 | 0.041 | 0.50 | 1 | 0.481 |
| 6-7 weeks | 3.35 | 1 | 0.067 | 0.33 | 1 | 0.565 | 1.19 | 1 | 0.275 | 0.00 | 1 | 0.973 |
| 8-9 weeks | 0.77 | 1 | 0.381 | 0.09 | 1 | 0.760 | 0.47 | 1 | 0.494 | 0.51 | 1 | 0.475 |
| 10-11 weeks | 0.39 | 1 | 0.533 | 0.54 | 1 | 0.461 | 0.42 | 1 | 0.515 | 0.34 | 1 | 0.559 |
| **Time since previous COVID-19 vaccination** | | | | | | | | | | | | |
| <24 weeks | 0.60 | 1 | 0.439 | 0.26 | 1 | 0.611 | 0.96 | 1 | 0.326 | 0.39 | 1 | 0.532 |
| **Sex** | | | | | | | | | | | | |
| Male | 3.00 | 1 | 0.083 | 1.04 | 1 | 0.307 | <0.01 | 1 | 0.981 | 0.24 | 1 | 0.626 |
| **Age** | | | | | | | | | | | | |
| 50-64 years | 9.96 | 1 | 0.002 | 1.50 | 1 | 0.220 | 1.35 | 1 | 0.246 | 0.01 | 1 | 0.904 |
| 65-79 years | 0.50 | 1 | 0.480 | 0.36 | 1 | 0.546 | 0.00 | 1 | 0.962 | 1.98 | 1 | 0.159 |
| ≥80 years | 7.03 | 1 | 0.008 | 0.05 | 1 | 0.816 | 0.20 | 1 | 0.654 | 0.88 | 1 | 0.348 |
| **Ethnicity** |  |  |  |  |  |  |  |  |  |  |  |  |
| Asian | 0.59 | 1 | 0.442 | - | - | - | - | - | - | - | - | - |
| Black | 0.20 | 1 | 0.657 | - | - | - | - | - | - | - | - | - |
| Mixed | 0.24 | 1 | 0.625 | - | - | - | - | - | - | - | - | - |
| Other | 3.09 | 1 | 0.079 | - | - | - | - | - | - | - | - | - |
| (Missing) | 0.38 | 1 | 0.535 | - | - | - | - | - | - | - | - | - |
| **BMI** | | | | | | | | | | | | |
| <18.5 | 6.15 | 1 | 0.013 | - | - | - | 0.46 | 1 | 0.496 | 2.06 | 1 | 0.151 |
| 18.5-24.9 | 8.20 | 1 | 0.004 | - | - | - | 0.07 | 1 | 0.788 | 0.11 | 1 | 0.735 |
| 30.0-39.9 | 9.27 | 1 | 0.002 | - | - | - | 3.73 | 1 | 0.053 | 0.21 | 1 | 0.646 |
| ≥40.0 | 0.47 | 1 | 0.491 | - | - | - | 0.06 | 1 | 0.811 | 1.34 | 1 | 0.247 |
| **Number of QCovid risk groups** | | | | | | | | | | | | |
| 1 | 0.09 | 1 | 0.768 | - | - | - | 0.34 | 1 | 0.560 | 2.37 | 1 | 0.123 |
| 2 | 0.42 | 1 | 0.515 | - | - | - | 0.07 | 1 | 0.788 | 2.05 | 1 | 0.152 |
| 3 | 4.49 | 1 | 0.034 | - | - | - | 0.53 | 1 | 0.465 | 4.78 | 1 | 0.029 |
| 4 | 0.48 | 1 | 0.490 | - | - | - | 0.09 | 1 | 0.767 | 0.89 | 1 | 0.346 |
| ≥5 | 3.36 | 1 | 0.067 | - | - | - | 1.16 | 1 | 0.281 | 0.47 | 1 | 0.495 |
| **Number of BNF risk groups** | | | | | | | | | | | | |
| 1 | - | - | - | 0.59 | 1 | 0.443 | - | - | - | - | - | - |
| 2 | - | - | - | 0.00 | 1 | 0.965 | - | - | - | - | - | - |
| 3 | - | - | - | 0.10 | 1 | 0.747 | - | - | - | - | - | - |
| 4 | - | - | - | 1.25 | 1 | 0.264 | - | - | - | - | - | - |
| 5 | - | - | - | 0.74 | 1 | 0.389 | - | - | - | - | - | - |
| ≥6 | - | - | - | 0.28 | 1 | 0.599 | - | - | - | - | - | - |
| **Household size** | | | | | | | | | | | | |
| 1 person | - | - | - | 0.04 | 1 | 0.847 | 0.36 | 1 | 0.550 | 0.07 | 1 | 0.793 |
| 3-5 people | - | - | - | 0.49 | 1 | 0.482 | 0.97 | 1 | 0.324 | 1.09 | 1 | 0.296 |
| 6-10 people | - | - | - | 1.31 | 1 | 0.252 | 0.88 | 1 | 0.348 | 4.70 | 1 | 0.030 |
| 11+ people | - | - | - | 0.12 | 1 | 0.730 | 0.06 | 1 | 0.813 | 1.59 | 1 | 0.207 |
| **Socioeconomic deprivation quintile** | | | | | | | | | | | | |
| 1st (Most) | 8.14 | 1 | 0.004 | 0.14 | 1 | 0.706 | 2.67 | 1 | 0.102 | 0.31 | 1 | 0.575 |
| 2nd | 0.14 | 1 | 0.710 | 0.19 | 1 | 0.665 | 1.02 | 1 | 0.312 | 0.21 | 1 | 0.651 |
| 3rd | 0.63 | 1 | 0.428 | 0.01 | 1 | 0.908 | 0.00 | 1 | 0.967 | 0.66 | 1 | 0.416 |
| 4th | 1.69 | 1 | 0.193 | 0.10 | 1 | 0.748 | 0.51 | 1 | 0.473 | 0.11 | 1 | 0.735 |
| **Rural/urban area classification** | | | | | | | | | | | | |
| Rural | 4.23 | 1 | 0.040 | 0.09 | 1 | 0.759 | 2.32 | 1 | 0.128 | 0.23 | 1 | 0.632 |

## **Figure S1a:** Smoothed trend of the Schoenfeld residuals for each variable in the overall main Cox regression model for England.


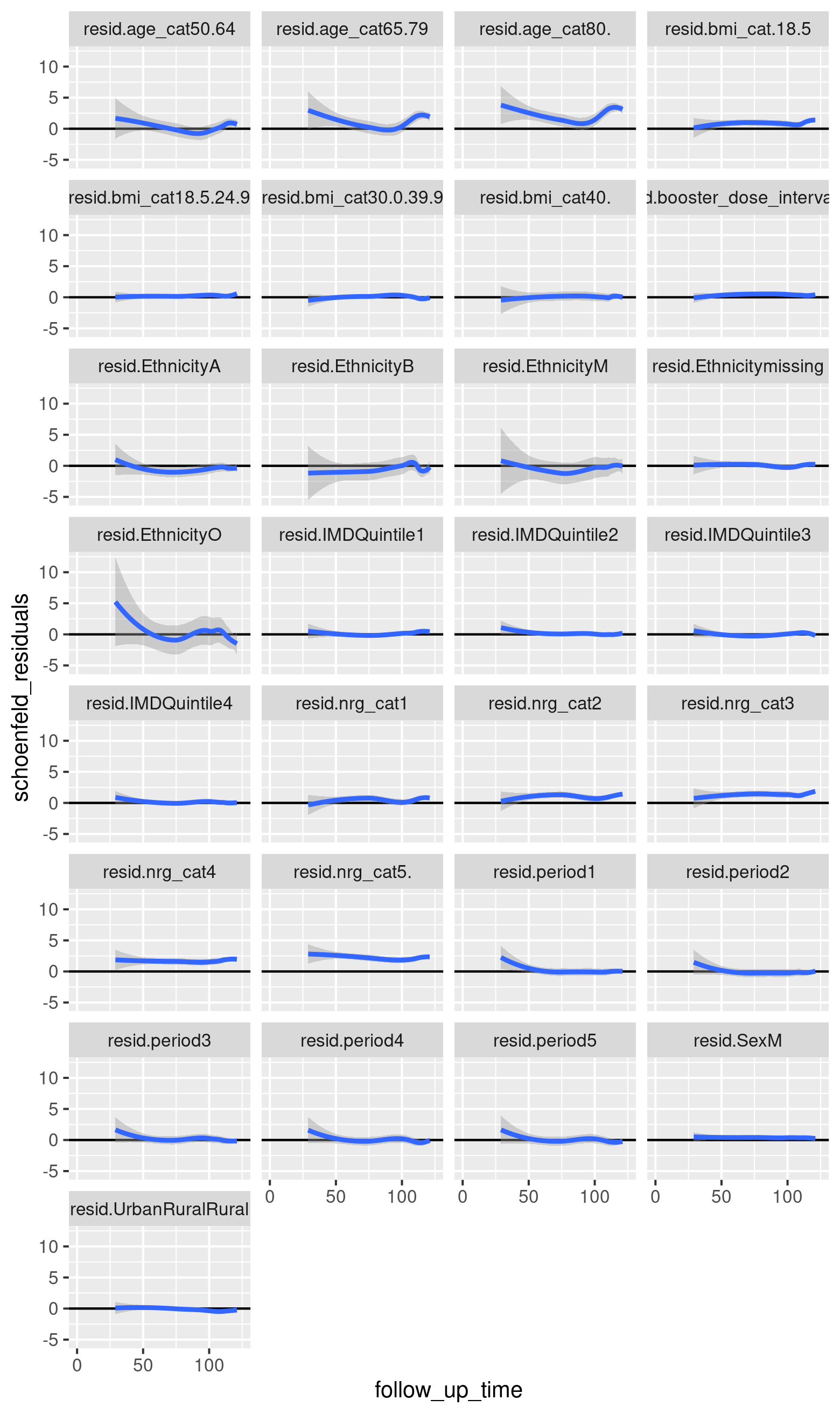


## **Figure S1b:** Smoothed trend of the Schoenfeld residuals for each variable in the overall main Cox regression model for Northern Ireland.


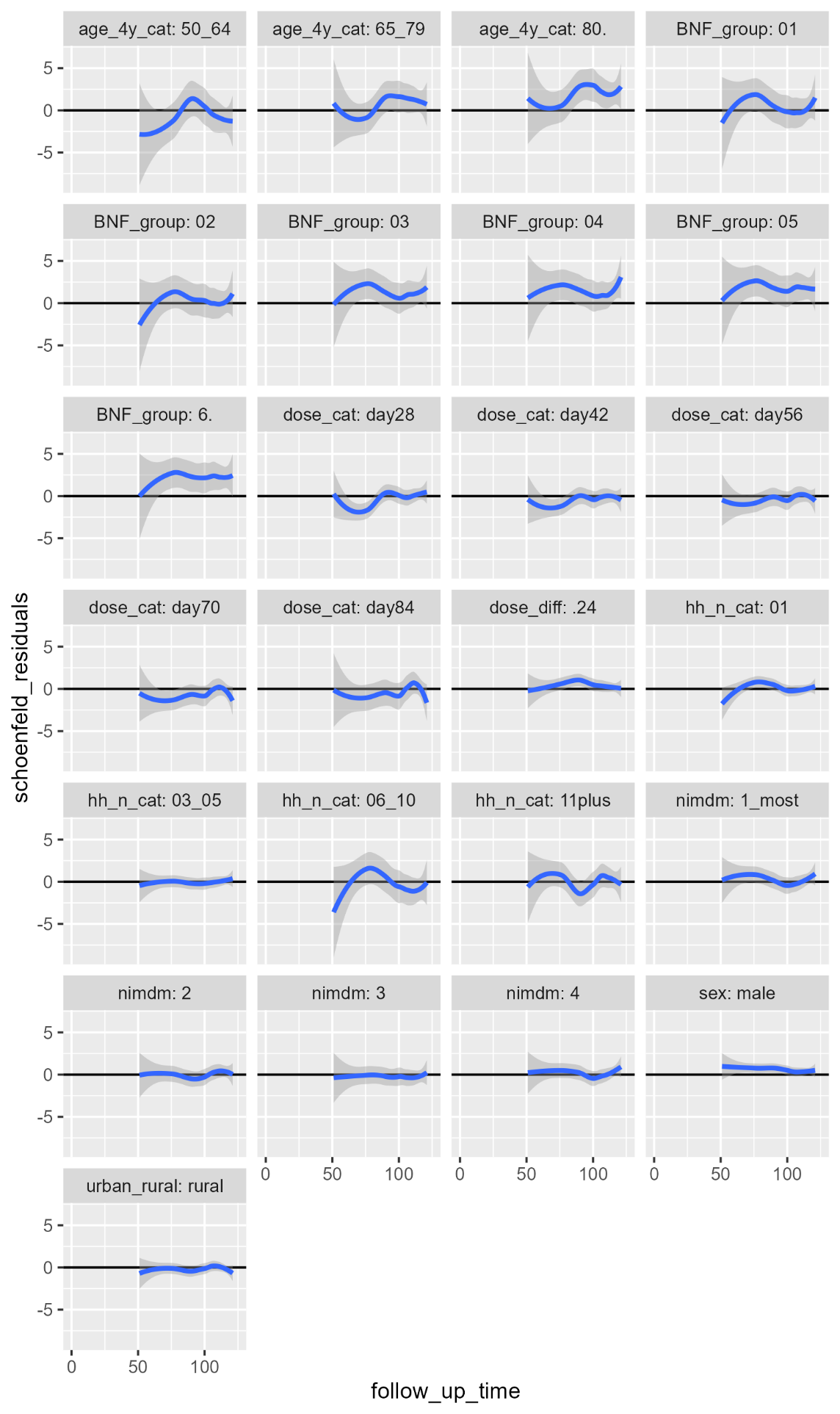


## **Figure S1c:** Smoothed trend of the Schoenfeld residuals for each variable in the overall main Cox regression model for Scotland.


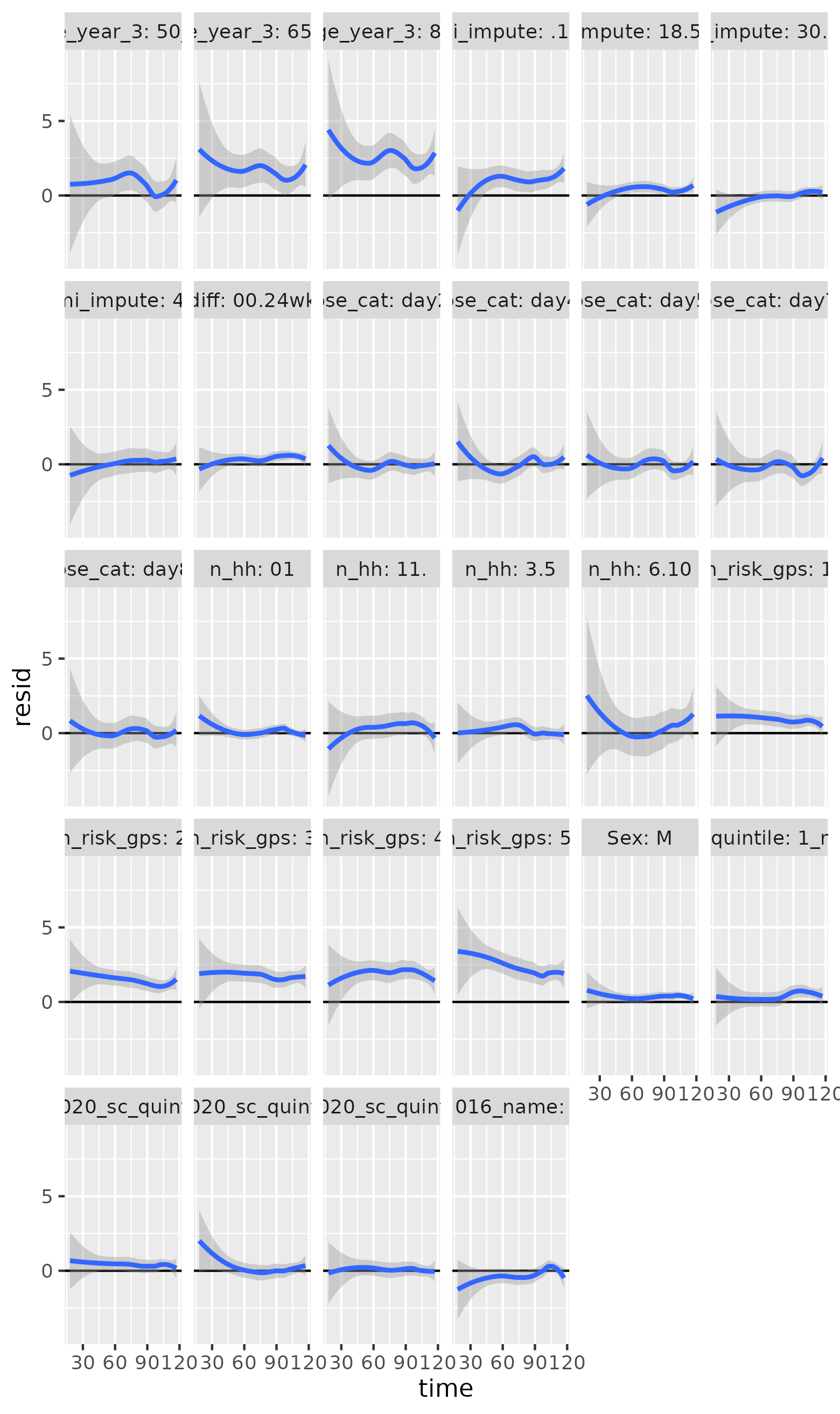


## **Figure S1d:** Smoothed trend of the Schoenfeld residuals for each variable in the overall main Cox regression model for Wales.


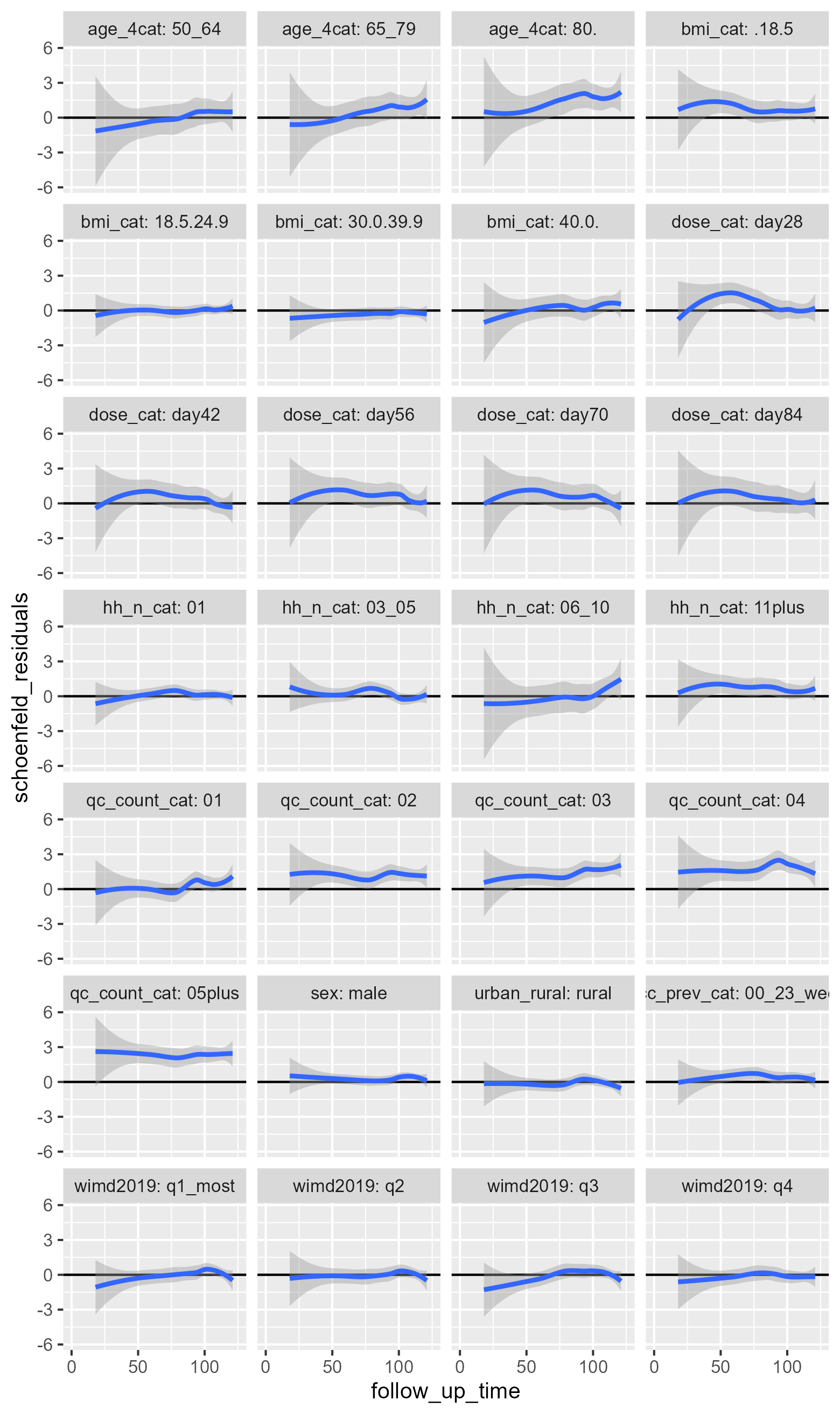


##

## **Table S10:** Nation and pooled counts and rates of severe COVID-19 outcome for individual Qcovid clinical conditions, across England, Scotland and Wales, only. Counts between 1 and 9 have been suppressed, all other counts rounded to nearest 10.

|  | **England** | | **Scotland** | | **Wales** | | **Pooled** | |
| --- | --- | --- | --- | --- | --- | --- | --- | --- |
|  | **n (%)** | **Outcome (rate)** | **n (%)** | **Outcome (rate)** | **n (%)** | **Outcome (rate)** | **n (%)** | **Outcome (rate)** |
| **Total** | 4,348,220 (100.0%) | 1,990 (2.8) | 1,829,690 (100.0%) | 820 (2.9) | 818,690 (100.0%) | 450 (3.3) | 6,996,600 (100.0%) | 3,260 (2.9) |
| Anti-leukotriene or LABA | 344,610 (7.9%) | 320 (5.4) | - | - | 25,740 (3.1%) | 40 (9.0) | 370,350 (7.2%) | 360 (5.7) |
| Asthma | 646,300 (14.9%) | 310 (2.9) | 242,630 (13.3%) | 130 (3.7) | 124,600 (15.2%) | 90 (4.4) | 1,013,530 (14.5%) | 530 (3.3) |
| Atrial fibrillation | 265,590 (6.1%) | 450 (9.1) | 89,770 (4.9%) | 140 (8.5) | 49,180 (6.0%) | 100 (10.7) | 404,530 (5.8%) | 690 (9.2) |
| Blood or bone marrow cancer | 67,260 (1.5%) | 120 (9.9) | 14,540 (0.8%) | 30 (13.7) | 7,780 (0.9%) | 20 (12.6) | 89,580 (1.3%) | 170 (10.7) |
| Bone marrow or stem cell transplant | 3,400 (0.1%) | - | - | - | - | - | 3,400 (0.1%) | - |
| Cerebral palsy | 7,940 (0.2%) | - | 3,640 (0.2%) | - | 820 (0.1%) | 0 (0.0) | 12,390 (0.2%) | - |
| Chemotherapy | 50,890 (1.2%) | 60 (6.3) | - | - | 4,370 (0.5%) | 10 (11.7) | 55,260 (1.1%) | 60 (6.2) |
| CKD Stage 3 | - | - | 132,870 (7.3%) | 220 (8.8) | 51,110 (6.2%) | 100 (10.2) | 183,980 (6.9%) | 320 (9.3) |
| CKD Stage 4 | - | - | 50 (0.0%) | - | 2,600 (0.3%) | 20 (39.9) | 2,650 (0.1%) | 20 (39.2) |
| CKD Stage 5 | - | - | 50 (0.0%) | 0 (0.0) | 3,780 (0.5%) | 10 (13.5) | 3,830 (0.1%) | 10 (13.4) |
| Congenital heart disease | 38,750 (0.9%) | 20 (3.6) | 11,210 (0.6%) | - | 12,340 (1.5%) | 20 (9.1) | 62,290 (0.9%) | 50 (4.8) |
| COPD | 200,950 (4.6%) | 340 (9.4) | 83,700 (4.6%) | 170 (11.7) | 42,430 (5.2%) | 100 (12.9) | 327,080 (4.7%) | 610 (10.5) |
| Coronary heart disease | 342,460 (7.9%) | 440 (6.9) | 147,770 (8.1%) | 190 (7.4) | 64,510 (7.9%) | 90 (7.5) | 554,740 (7.9%) | 720 (7.1) |
| Cystic fibrosis or bronchiectasis | 55,600 (1.3%) | 70 (6.5) | 18,270 (1.0%) | 40 (12.7) | 7,310 (0.9%) | 20 (14.6) | 81,170 (1.2%) | 130 (8.8) |
| Dementia | 83,470 (1.9%) | 390 (24.5) | 16,470 (0.9%) | 40 (11.8) | 12,990 (1.6%) | 60 (23.0) | 112,930 (1.6%) | 490 (22.2) |
| Diabetes Type 1 | - | - | 14,300 (0.8%) | - | 4,570 (0.6%) | - | 18,870 (0.7%) | - |
| Diabetes Type 2 | - | - | 188,150 (10.3%) | 170 (5.5) | 112,370 (13.7%) | 120 (6.1) | 300,520 (11.3%) | 290 (5.7) |
| Down's syndrome | - | - | 1,190 (0.1%) | - | 160 (0.0%) | 0 (0.0) | 1,350 (0.1%) | - |
| Epilepsy | 99,250 (2.3%) | 60 (3.9) | 37,120 (2.0%) | 30 (4.9) | 13,490 (1.6%) | 20 (9.6) | 149,860 (2.1%) | 110 (4.7) |
| Heart failure | 144,310 (3.3%) | 300 (11.2) | 34,720 (1.9%) | 60 (10.4) | 23,660 (2.9%) | 60 (13.5) | 202,690 (2.9%) | 420 (11.3) |
| Immunosuppressants | 48,240 (1.1%) | 50 (6.1) | 1,000 (0.1%) | - | 2,060 (0.3%) | - | 51,300 (0.7%) | 50 (5.8) |
| Learning disability | - | - | 22,550 (1.2%) | - | 12,650 (1.5%) | - | 35,200 (1.3%) | - |
| Liver cirrhosis | 20,140 (0.5%) | 30 (7.8) | 7,690 (0.4%) | - | 4,860 (0.6%) | - | 32,690 (0.5%) | 30 (5.4) |
| Lung or oral cancer | 23,270 (0.5%) | 40 (10.4) | 3,880 (0.2%) | - | 4,100 (0.5%) | - | 31,250 (0.4%) | 50 (9.0) |
| Motor neurone disease or MS | 27,520 (0.6%) | 30 (5.8) | 13,720 (0.7%) | - | 3,870 (0.5%) | - | 45,110 (0.6%) | 40 (5.4) |
| Parkinson's disease | 26,410 (0.6%) | 70 (14.9) | 7,560 (0.4%) | 20 (11.7) | 4,220 (0.5%) | 10 (12.9) | 38,190 (0.5%) | 100 (14.2) |
| Peripheral vascular disease | 63,360 (1.5%) | 120 (10.2) | 9,300 (0.5%) | - | 12,300 (1.5%) | 30 (13.3) | 84,960 (1.2%) | 160 (10.4) |
| Prednisolone | 1,022,240 (23.5%) | 790 (4.5) | - | - | 10,370 (1.3%) | 30 (15.5) | 1,032,610 (20.0%) | 820 (4.6) |
| Prior fracture | 235,700 (5.4%) | 270 (6.5) | 109,580 (6.0%) | 120 (6.6) | 39,590 (4.8%) | 50 (7.1) | 384,870 (5.5%) | 440 (6.6) |
| Pulmonary hypertension | 28,630 (0.7%) | 70 (13.6) | 5,110 (0.3%) | 20 (16.4) | 2,800 (0.3%) | - | 36,540 (0.5%) | 90 (13.5) |
| Radiotherapy | 45,310 (1.0%) | 50 (6.5) | - | - | 1,010 (0.1%) | - | 46,320 (0.9%) | 50 (6.0) |
| Rheumatoid arthritis or SLE | 106,010 (2.4%) | 80 (4.6) | 57,780 (3.2%) | 40 (4.5) | 15,600 (1.9%) | 20 (6.6) | 179,390 (2.6%) | 140 (4.6) |
| Severe mental illness | 723,790 (16.6%) | 380 (3.3) | 34,580 (1.9%) | 10 (2.7) | 117,010 (14.3%) | 80 (4.2) | 875,380 (12.5%) | 480 (3.4) |
| Sickle cell or severe combined immunodeficiency | 10,230 (0.2%) | - | 140 (0.0%) | 0 (0.0) | 720 (0.1%) | - | 11,090 (0.2%) | - |
| Solid organ transplant | 7,100 (0.2%) | 10 (8.5) | - | - | 590 (0.1%) | 0 (0.0) | 7,690 (0.1%) | 10 (7.7) |
| Stroke or TIA | 212,740 (4.9%) | 350 (8.9) | 87,370 (4.8%) | 140 (8.9) | 39,940 (4.9%) | 80 (10.8) | 340,050 (4.9%) | 560 (9.0) |
| Thrombosis or pulmonary embolus | 75,170 (1.7%) | 100 (7.6) | 31,210 (1.7%) | 40 (7.2) | 31,120 (3.8%) | 40 (7.3) | 137,500 (2.0%) | 180 (7.5) |

## **Table S11:** Nation and meta, adjusted hazard ratios with 95% confidence intervals, for specific clinical conditions associated with severe COVID-19 outcomes, across England, Scotland and Wales, only.

|  | **England** | **Scotland** | **Wales** | **Meta-analysis** | **Q-test** |
| --- | --- | --- | --- | --- | --- |
| Down's syndrome | - | 31.40 (11.45, 86.07) | 0.00 (0.00, Inf) | 31.40 (11.45, 86.07) | 0.00 (p=0.9870) |
| Solid organ transplant | 7.22 (5.03, 10.36) | - | 0.00 (0.00, Inf) | 7.22 (5.03, 10.36) | 0.00 (p=0.9841) |
| CKD Stage 4 | - | 23.80 (3.33, 170.42) | 5.81 (3.54, 9.52) | 6.31 (3.91, 10.20) | 1.86 (p=0.1731) |
| CKD Stage 5 | - | 0.00 (0.00, Inf) | 3.49 (1.91, 6.39) | 3.49 (1.91, 6.39) | 0.00 (p=0.9866) |
| Immunosuppressants | 3.04 (2.52, 3.68) | 2.61 (0.37, 18.57) | 2.57 (0.82, 8.06) | 3.03 (2.51, 3.65) | 0.10 (p=0.9494) |
| Bone marrow or stem cell transplant | 2.92 (1.31, 6.52) | - | - | 2.92 (1.31, 6.52) | 0.00 (p=1.0000) |
| Dementia | 2.98 (2.71, 3.27) | 1.29 (0.91, 1.84) | 3.16 (2.27, 4.39) | 2.84 (2.60, 3.10) | 20.51 (p=<0.0001) |
| Sickle cell or severe combined immunodeficiency | 2.21 (1.31, 3.75) | 0.00 (0.00, Inf) | 6.59 (2.11, 20.57) | 2.68 (1.66, 4.33) | 2.91 (p=0.2339) |
| Pulmonary hypertension | 2.70 (2.26, 3.23) | 2.96 (1.77, 4.94) | 1.19 (0.44, 3.18) | 2.66 (2.25, 3.14) | 2.76 (p=0.2519) |
| Blood or bone marrow cancer | 2.56 (2.23, 2.93) | 3.40 (2.40, 4.83) | 2.29 (1.40, 3.73) | 2.63 (2.33, 2.97) | 2.55 (p=0.2790) |
| Parkinson's disease | 2.63 (2.19, 3.16) | 2.24 (1.36, 3.68) | 2.02 (1.07, 3.81) | 2.54 (2.15, 3.00) | 0.89 (p=0.6422) |
| COPD | 2.18 (1.99, 2.39) | 3.11 (2.61, 3.70) | 3.45 (2.75, 4.31) | 2.46 (2.28, 2.65) | 22.42 (p=<0.0001) |
| Motor neurone disease or MS | 2.61 (1.98, 3.44) | 1.79 (0.89, 3.60) | 0.52 (0.07, 3.67) | 2.41 (1.87, 3.12) | 3.38 (p=0.1847) |
| Cerebral palsy | 1.84 (0.87, 3.87) | 4.27 (1.36, 13.33) | 0.00 (0.00, Inf) | 2.36 (1.27, 4.41) | 1.47 (p=0.4791) |
| Liver cirrhosis | 2.53 (1.89, 3.38) | 0.52 (0.13, 2.07) | 1.65 (0.68, 3.99) | 2.29 (1.74, 3.00) | 5.38 (p=0.0679) |
| Heart failure | 2.12 (1.93, 2.33) | 1.97 (1.52, 2.55) | 2.49 (1.90, 3.27) | 2.14 (1.96, 2.33) | 1.66 (p=0.4358) |
| Chemotherapy | 1.96 (1.61, 2.40) | - | 4.12 (2.30, 7.36) | 2.13 (1.76, 2.57) | 5.56 (p=0.0183) |
| Diabetes Type 1 | - | 2.02 (0.90, 4.53) | 2.28 (0.73, 7.13) | 2.11 (1.09, 4.07) | 0.03 (p=0.8658) |
| Lung or oral cancer | 2.12 (1.66, 2.71) | 1.95 (0.81, 4.70) | 2.03 (0.96, 4.30) | 2.10 (1.68, 2.63) | 0.04 (p=0.9802) |
| Peripheral vascular disease | 2.06 (1.79, 2.36) | 1.18 (0.61, 2.28) | 2.47 (1.69, 3.61) | 2.06 (1.81, 2.34) | 3.63 (p=0.1628) |
| Cystic fibrosis or bronchiectasis | 1.66 (1.39, 1.98) | 3.14 (2.28, 4.32) | 2.86 (1.78, 4.59) | 2.00 (1.73, 2.31) | 14.20 (p=0.0008) |
| Anti-leukotriene or LABA | 1.92 (1.76, 2.10) | - | 2.56 (1.83, 3.57) | 1.96 (1.79, 2.13) | 2.64 (p=0.1044) |
| Thrombosis or pulmonary embolus | 2.02 (1.74, 2.33) | 1.79 (1.28, 2.49) | 1.69 (1.24, 2.32) | 1.93 (1.71, 2.18) | 1.20 (p=0.5478) |
| Epilepsy | 1.71 (1.42, 2.05) | 1.90 (1.28, 2.81) | 2.58 (1.56, 4.26) | 1.81 (1.54, 2.12) | 2.35 (p=0.3091) |
| Stroke or TIA | 1.77 (1.62, 1.93) | 1.84 (1.53, 2.22) | 2.08 (1.63, 2.66) | 1.81 (1.67, 1.95) | 1.57 (p=0.4562) |
| Atrial fibrillation | 1.82 (1.68, 1.97) | 1.64 (1.36, 1.98) | 1.80 (1.43, 2.28) | 1.79 (1.67, 1.92) | 1.01 (p=0.6048) |
| CKD Stage 3 | - | 1.74 (1.48, 2.05) | 1.87 (1.48, 2.37) | 1.78 (1.56, 2.04) | 0.25 (p=0.6186) |
| Prednisolone | 1.66 (1.55, 1.77) | - | 3.81 (2.65, 5.46) | 1.70 (1.59, 1.82) | 19.67 (p=<0.0001) |
| Radiotherapy | 1.65 (1.35, 2.02) | - | 1.09 (0.15, 7.78) | 1.64 (1.34, 2.01) | 0.17 (p=0.6810) |
| Coronary heart disease | 1.59 (1.46, 1.72) | 1.66 (1.41, 1.96) | 1.45 (1.14, 1.84) | 1.59 (1.48, 1.70) | 0.83 (p=0.6597) |
| Rheumatoid arthritis or SLE | 1.56 (1.33, 1.82) | 1.39 (1.01, 1.92) | 2.22 (1.46, 3.38) | 1.58 (1.39, 1.81) | 3.20 (p=0.2015) |
| Prior fracture | 1.59 (1.44, 1.75) | 1.59 (1.30, 1.94) | 1.43 (1.06, 1.93) | 1.57 (1.45, 1.71) | 0.43 (p=0.8057) |
| Diabetes Type 2 | - | 1.50 (1.27, 1.78) | 1.64 (1.33, 2.03) | 1.56 (1.36, 1.78) | 0.41 (p=0.5231) |
| Congenital heart disease | 1.57 (1.20, 2.06) | 1.01 (0.32, 3.13) | 1.60 (0.97, 2.64) | 1.55 (1.22, 1.95) | 0.58 (p=0.7471) |
| Severe mental illness | 1.45 (1.33, 1.57) | 1.23 (0.71, 2.14) | 1.61 (1.26, 2.07) | 1.46 (1.35, 1.57) | 1.04 (p=0.5952) |
| Asthma | 1.33 (1.22, 1.45) | 1.58 (1.31, 1.91) | 1.49 (1.18, 1.88) | 1.39 (1.29, 1.49) | 3.07 (p=0.2155) |
| Learning disability | - | 1.07 (0.53, 2.16) | 1.04 (0.51, 2.12) | 1.06 (0.64, 1.74) | 0.00 (p=0.9508) |

# Autumn booster vaccination and COVID-19 therapeutics

## **Table S12:** Descriptive characteristics and rates of severe COVID-19 outcomes for individuals who received an Autumn 2022 booster and COVID-19 therapeutic treatment, either before or after booster vaccination, in Scotland only.

|  | **Therapeutic treatment before Autumn booster** | | **Therapeutic treatment after Autumn booster** | |
| --- | --- | --- | --- | --- |
|  | **n (%)** | **Events (rate)** | **n (%)** | **Events (rate)** |
| **Total** | 11,150 (100.0%) | 10 (7.8) | 690 (100.0%) | 100 (883.4) |
| **Number of previous COVID-19 vaccinations** | | | | |
| 3 | 3,270 (29.3%) | - | 150 (21.7%) | 30 (1126.9) |
| 4 | 4,770 (42.7%) | - | 370 (53.7%) | 60 (986.8) |
| 5 | 3,120 (28.0%) | - | 170 (24.6%) | 10 (469.8) |
| **Time since previous COVID-19 vaccination** | | | | |
| <24 weeks | 4,560 (40.9%) | - | 350 (51.2%) | 50 (812.4) |
| ≥24 weeks | 6,590 (59.1%) | - | 340 (48.8%) | 50 (975.4) |
| **Sex** |  |  |  |  |
| Female | 6,600 (59.1%) | - | 380 (55.6%) | 50 (701.8) |
| Male | 4,560 (40.9%) | - | 310 (44.4%) | 60 (1122.9) |
| **Age** | | | | |
| 18-49 years | 2,960 (26.6%) | - | 90 (13.3%) | - |
| 50-64 years | 4,020 (36.1%) | - | 180 (25.5%) | 20 (535.8) |
| 65-79 years | 3,370 (30.2%) | - | 270 (39.7%) | 50 (1080.7) |
| ≥80 years | 800 (7.2%) | - | 150 (21.6%) | 40 (1314.4) |
| **Ethnicity** | | | | |
| White | 9,880 (88.6%) | - | 610 (88.1%) | 90 (898.3) |
| Asian | 120 (1.0%) | - | - | - |
| Black | 30 (0.2%) | - | - | 0 (0.0) |
| Mixed | 30 (0.3%) | - | - | - |
| Other | 30 (0.2%) | - | - | - |
| Unknown | 1,080 (9.7%) | - | 70 (10.7%) | - |
| **BMI** | | | | |
| <18.5 | 150 (1.3%) | - | 20 (2.5%) | <10 |
| 18.5-24.9 | 1,870 (16.8%) | - | 150 (21.3%) | 30 (1013.7) |
| 25.0-29.9 | 5,890 (52.8%) | - | 320 (46.0%) | 40 (696.1) |
| 30.0-39.9 | 2,670 (24.0%) | - | 170 (24.6%) | 30 (1009.3) |
| ≥40.0 | 570 (5.2%) | - | 40 (5.6%) | <10 |
| **Number of QCovid risk groups** | | | | |
| 0 | 2,330 (20.9%) | - | 120 (18.0%) | 10 (454.0) |
| 1 | 4,770 (42.7%) | - | 260 (38.1%) | 40 (843.0) |
| 2 | 2,380 (21.4%) | - | 150 (21.9%) | 30 (1120.6) |
| 3 | 1,040 (9.3%) | - | 80 (12.2%) | 10 (894.6) |
| 4 | 430 (3.8%) | - | 40 (5.2%) | - |
| ≥5 | 210 (1.9%) | - | 30 (4.8%) | - |
| **Household size** | | | | |
| 2 people | 4,190 (37.6%) | - | 270 (39.4%) | 40 (795.2) |
| 1 person | 3,200 (28.7%) | - | 270 (39.1%) | 50 (1012.8) |
| 3-5 people | 3,560 (31.9%) | - | 140 (20.8%) | 20 (806.6) |
| 6-10 people | 180 (1.6%) | - | - | - |
| ≥11 people | 20 (0.2%) | - | - | 0 (0.0) |
| **Socioeconomic deprivation quintile** | | | | |
| 5th (Least) | 3,000 (26.9%) | - | 150 (21.6%) | 20 (614.9) |
| 4th | 2,640 (23.7%) | - | 140 (20.8%) | 20 (767.0) |
| 3rd | 2,140 (19.2%) | - | 140 (20.8%) | 20 (658.2) |
| 2nd | 1,900 (17.1%) | - | 140 (19.7%) | 30 (1074.5) |
| 1st (Most) | 1,470 (13.2%) | - | 120 (17.1%) | 30 (1445.9) |
| **Rural/urban area classification** | | | | |
| Urban | 9,500 (85.1%) | - | 580 (84.1%) | 100 (1006.7) |
| Rural | 1,660 (14.9%) | - | 110 (15.9%) | - |
| **Therapeutics before booster** | | | | |
| 0 | - | - | 620 (90.2%) | 100 (968.9) |
| 1 | 2,800 (25.1%) | - | 20 (3.0%) | 0 (0.0) |
| 2 | 8,200 (73.5%) | - | 50 (6.5%) | - |
| 3 | 140 (1.2%) | - | - | 0 (0.0) |
| 4 | 20 (0.2%) | - | - | - |
| **Therapeutics after booster** | | | | |
| 0 | 11,090 (99.4%) | - | - | - |
| 1 | - | - | 80 (12.2%) | 20 (1217.3) |
| 2 | 70 (0.6%) | - | 600 (86.3%) | 80 (798.2) |
| 3 | - | - | 10 (1.5%) | - |
| 4 | - | - | - | 0 (0.0) |
| **Type of treatment** | | | | |
| Multiple therapeutics | 160 (1.4%) | - | 30 (4.3%) | <10 |
| Not specified | 20 (0.2%) | - | - | 0 (0.0) |
| Only antiviral treatment | 8,200 (73.5%) | - | 580 (83.4%) | 80 (827.1) |
| Only monoclonal antibody | 2,780 (24.9%) | - | 80 (12.2%) | 20 (1217.3) |

# Sensitivity analysis: Outcome under broad definitions

## **Table S13:** Nation and pooled sample characteristics and rates of broad severe COVID-19 outcomes for individuals who received an Autumn 2022 booster vaccination. Rates are number of events per 1,000 person-years. Counts between 1 and 9 have been suppressed, all other counts rounded to nearest 10.

|  | **England** | | **Northern Ireland** | | **Scotland** | | **Wales** | | **Pooled** | |
| --- | --- | --- | --- | --- | --- | --- | --- | --- | --- | --- |
|  | **n (%)** | **Outcome (rate)** | **n (%)** | **Outcome (rate)** | **n (%)** | **Outcome (rate)** | **n (%)** | **Outcome (rate)** | **n (%)** | **Outcome (rate)** |
| **Overall** | | | | | | | | | | |
| **Total** | 4,348,220 (100.0%) | 3,800 (5.3) | 455,230 (100.0%) | 610 (8.9) | 1,829,210 (100.0%) | 1,620 (5.8) | 818,640 (100.0%) | 890 (6.6) | 7,451,300 (100.0%) | 6,910 (5.7) |
| **Number of previous COVID-19 vaccinations** | | | | | | | | | | |
| 3 | 3,133,430 (72.1%) | 1,090 (2.2) | 345,120 (75.8%) | 210 (4.1) | 1,349,530 (73.8%) | 430 (2.3) | 584,840 (71.4%) | 220 (2.4) | 5,412,910 (72.6%) | 1,940 (2.4) |
| 4 | 1,121,880 (25.8%) | 2,520 (11.5) | 105,900 (23.3%) | 400 (22.2) | 453,670 (24.8%) | 1,130 (13.0) | 212,390 (25.9%) | 600 (14.1) | 1,893,840 (25.4%) | 4,640 (12.7) |
| 5 | 92,910 (2.1%) | 200 (11.8) | 4,220 (0.9%) | - | 26,020 (1.4%) | 60 (15.2) | 21,410 (2.6%) | 70 (16.3) | 144,560 (1.9%) | 330 (12.9) |
| **Time since previous COVID-19 vaccination** | | | | | | | | | | |
| <24 weeks | 649,840 (14.9%) | 1,580 (11.9) | 74,000 (16.3%) | 330 (23.9) | 292,550 (16.0%) | 780 (12.9) | 152,110 (18.6%) | 490 (14.2) | 1,168,500 (15.7%) | 3,180 (13.2) |
| ≥24 weeks | 3,698,380 (85.1%) | 2,220 (3.8) | 381,230 (83.7%) | 290 (5.2) | 1,536,660 (84.0%) | 840 (3.8) | 666,530 (81.4%) | 400 (3.9) | 6,282,800 (84.3%) | 3,740 (3.9) |
| **Sex** | | | | | | | | | | |
| Female | 2,343,540 (53.9%) | 1,870 (4.8) | 244,630 (53.7%) | 300 (7.9) | 1,010,360 (55.2%) | 810 (5.1) | 441,970 (54.0%) | 450 (6.0) | 4,040,500 (54.2%) | 3,430 (5.2) |
| Male | 2,004,680 (46.1%) | 1,930 (5.8) | 210,600 (46.3%) | 320 (10.0) | 818,850 (44.8%) | 800 (6.6) | 376,670 (46.0%) | 440 (7.3) | 3,410,800 (45.8%) | 3,490 (6.4) |
| **Age** | | | | | | | | | | |
| 18-49 years | 499,590 (11.5%) | 160 (2.1) | 51,770 (11.4%) | 10 (1.8) | 268,020 (14.7%) | 40 (1.0) | 100,690 (12.3%) | 30 (1.9) | 920,070 (12.3%) | 230 (1.7) |
| 50-64 years | 1,620,370 (37.3%) | 340 (1.5) | 166,420 (36.6%) | 30 (1.2) | 685,020 (37.4%) | 140 (1.8) | 295,290 (36.1%) | 80 (2.0) | 2,767,100 (37.1%) | 590 (1.6) |
| 65-79 years | 1,612,450 (37.1%) | 1,230 (4.1) | 173,230 (38.1%) | 210 (7.2) | 663,840 (36.3%) | 610 (5.1) | 310,130 (37.9%) | 290 (5.3) | 2,759,650 (37.0%) | 2,340 (4.6) |
| ≥80 years | 615,810 (14.2%) | 2,070 (17.1) | 63,810 (14.0%) | 370 (34.2) | 212,330 (11.6%) | 820 (19.2) | 112,530 (13.7%) | 490 (21.0) | 1,004,480 (13.5%) | 3,760 (19.0) |
| **Ethnicity** | | | | | | | | | | |
| White | 3,796,620 (87.3%) | 3,430 (5.4) | - | - | 1,334,240 (72.9%) | 1,380 (6.7) | 792,510 (96.8%) | 870 (6.7) | 5,923,380 (84.7%) | 5,690 (5.8) |
| Asian | 171,010 (3.9%) | 80 (3.0) | - | - | 20,990 (1.1%) | 20 (5.9) | 12,880 (1.6%) | 10 (4.6) | 204,880 (2.9%) | 110 (3.4) |
| Black | 51,420 (1.2%) | 40 (4.5) | - | - | 3,140 (0.2%) | 0 (0.0) | 2,100 (0.3%) | 0 (0.0) | 56,660 (0.8%) | 40 (4.6) |
| Mixed | 31,140 (0.7%) | 20 (3.9) | - | - | 3,810 (0.2%) | - | 2,940 (0.4%) | - | 37,890 (0.5%) | 20 (3.4) |
| Other | 24,080 (0.6%) | 10 (2.6) | - | - | 3,620 (0.2%) | 0 (0.0) | 2,580 (0.3%) | 0 (0.0) | 30,280 (0.4%) | 10 (2.1) |
| Unknown | 273,960 (6.3%) | 220 (5.0) | - | - | 463,410 (25.3%) | 210 (3.2) | 5,640 (0.7%) | - | 743,000 (10.6%) | 430 (3.9) |
| **BMI** | | | | | | | | | | |
| <18.5 | 75,900 (1.7%) | 200 (15.5) | - | - | 17,380 (1.0%) | 60 (20.8) | 14,220 (1.7%) | 40 (16.2) | 107,490 (1.5%) | 300 (16.7) |
| 18.5-24.9 | 1,388,650 (31.9%) | 1,390 (6.0) | - | - | 251,570 (13.8%) | 410 (10.0) | 205,550 (25.1%) | 290 (8.3) | 1,845,780 (26.4%) | 2,080 (6.8) |
| 25.0-29.9 | 1,572,030 (36.2%) | 1,250 (4.8) | - | - | 1,069,080 (58.4%) | 710 (4.4) | 277,180 (33.9%) | 300 (6.5) | 2,918,300 (41.7%) | 2,260 (4.8) |
| 30.0-39.9 | 1,115,970 (25.7%) | 840 (4.5) | - | - | 403,920 (22.1%) | 380 (6.0) | 275,520 (33.7%) | 220 (4.8) | 1,795,410 (25.7%) | 1,430 (4.9) |
| ≥40.0 | 195,670 (4.5%) | 130 (4.0) | - | - | 87,260 (4.8%) | 60 (5.0) | 46,170 (5.6%) | 40 (6.0) | 329,100 (4.7%) | 230 (4.5) |
| **Number of QCovid risk groups** | | | | | | | | | | |
| 0 | 1,567,960 (36.1%) | 340 (1.4) | - | - | 917,780 (50.2%) | 240 (1.8) | 354,210 (43.3%) | 110 (2.0) | 2,839,950 (40.6%) | 700 (1.6) |
| 1 | 1,168,760 (26.9%) | 590 (3.0) | - | - | 572,070 (31.3%) | 450 (5.1) | 241,210 (29.5%) | 180 (4.4) | 1,982,050 (28.3%) | 1,220 (3.8) |
| 2 | 704,880 (16.2%) | 700 (5.8) | - | - | 212,070 (11.6%) | 400 (11.4) | 120,710 (14.7%) | 190 (8.9) | 1,037,660 (14.8%) | 1,300 (7.3) |
| 3 | 431,530 (9.9%) | 680 (8.9) | - | - | 81,110 (4.4%) | 280 (19.5) | 57,000 (7.0%) | 160 (15.3) | 569,640 (8.1%) | 1,120 (11.2) |
| 4 | 242,600 (5.6%) | 530 (12.3) | - | - | 30,910 (1.7%) | 140 (25.6) | 26,340 (3.2%) | 120 (23.4) | 299,850 (4.3%) | 790 (14.7) |
| ≥5 | 232,490 (5.3%) | 960 (22.7) | - | - | 15,280 (0.8%) | 90 (33.1) | 19,160 (2.3%) | 140 (38.7) | 266,920 (3.8%) | 1,190 (24.5) |
| **Number of BNF risk groups** | | | | | | | | | | |
| 0 | - | - | 95,590 (21.0%) | 20 (1.4) | - | - | - | - | 95,590 (21.0%) | 20 (1.5) |
| 1 | - | - | 86,520 (19.0%) | 50 (3.6) | - | - | - | - | 86,520 (19.0%) | 50 (3.8) |
| 2 | - | - | 83,350 (18.3%) | 50 (4.2) | - | - | - | - | 83,350 (18.3%) | 50 (3.9) |
| 3 | - | - | 68,420 (15.0%) | 100 (9.4) | - | - | - | - | 68,420 (15.0%) | 100 (9.4) |
| 4 | - | - | 50,490 (11.1%) | 110 (14.2) | - | - | - | - | 50,490 (11.1%) | 110 (13.8) |
| 5 | - | - | 33,670 (7.4%) | 120 (21.8) | - | - | - | - | 33,670 (7.4%) | 120 (22.6) |
| ≥6 | - | - | 37,180 (8.2%) | 170 (28.1) | - | - | - | - | 37,180 (8.2%) | 170 (29.0) |
| **Household size** | | | | | | | | | | |
| 1 person | - | - | 77,330 (17.0%) | 200 (16.2) | 632,480 (34.6%) | 780 (7.7) | 155,550 (19.0%) | 290 (10.5) | 865,360 (27.9%) | 1,270 (9.0) |
| 2 people | - | - | 153,450 (33.7%) | 240 (9.9) | 697,320 (38.1%) | 590 (5.3) | 340,350 (41.6%) | 360 (6.3) | 1,191,120 (38.4%) | 1,190 (6.2) |
| 3-5 people | - | - | 197,270 (43.3%) | 140 (4.7) | 467,120 (25.5%) | 180 (2.9) | 285,800 (34.9%) | 140 (3.2) | 950,190 (30.6%) | 460 (3.4) |
| 6-10 people | - | - | 22,580 (5.0%) | 20 (7.3) | 22,070 (1.2%) | 20 (5.2) | 24,300 (3.0%) | 20 (6.1) | 68,940 (2.2%) | 60 (5.9) |
| ≥11 people | - | - | 4,600 (1.0%) | 20 (24.0) | 10,240 (0.6%) | 50 (21.6) | 12,640 (1.5%) | 80 (29.1) | 27,480 (0.9%) | 150 (26.5) |
| **Socioeconomic deprivation quintile** | | | | | | | | | | |
| 5th (Least) | 1,200,860 (27.6%) | 1,020 (5.1) | 113,650 (25.0%) | 140 (8.0) | 419,920 (23.0%) | 280 (4.3) | 207,530 (25.4%) | 230 (6.4) | 1,941,960 (26.1%) | 1,660 (5.2) |
| 4th | 1,041,070 (23.9%) | 880 (5.1) | 100,470 (22.1%) | 130 (8.8) | 407,630 (22.3%) | 300 (4.9) | 172,510 (21.1%) | 150 (5.3) | 1,721,680 (23.1%) | 1,460 (5.2) |
| 3rd | 894,460 (20.6%) | 730 (4.9) | 89,480 (19.7%) | 110 (8.0) | 381,950 (20.9%) | 330 (5.7) | 160,690 (19.6%) | 160 (6.3) | 1,526,580 (20.5%) | 1,340 (5.4) |
| 2nd | 712,070 (16.4%) | 640 (5.4) | 84,400 (18.5%) | 120 (9.1) | 338,880 (18.5%) | 380 (7.3) | 153,290 (18.7%) | 170 (6.9) | 1,288,640 (17.3%) | 1,310 (6.3) |
| 1st (Most) | 499,760 (11.5%) | 530 (6.5) | 67,240 (14.8%) | 110 (11.0) | 280,830 (15.4%) | 320 (7.5) | 124,630 (15.2%) | 170 (8.4) | 972,460 (13.1%) | 1,130 (7.3) |
| **Rural/urban area classification** | | | | | | | | | | |
| Urban | 3,304,990 (76.0%) | 3,060 (5.6) | 292,150 (64.2%) | 420 (9.5) | 1,533,630 (83.8%) | 1,410 (6.0) | 562,960 (68.8%) | 640 (6.8) | 5,693,730 (76.4%) | 5,530 (6.0) |
| Rural | 1,043,230 (24.0%) | 740 (4.3) | 163,080 (35.8%) | 190 (7.8) | 295,580 (16.2%) | 200 (4.6) | 255,680 (31.2%) | 250 (6.0) | 1,757,570 (23.6%) | 1,380 (4.9) |
| **Cominarty** | | | | | | | | | | |
| **Total** | 2,498,240 (100.0%) | 1,450 (4.4) | 444,840 (100.0%) | 600 (8.9) | 807,880 (100.0%) | 530 (5.0) | 397,750 (100.0%) | 260 (5.1) | 4,148,710 (100.0%) | 2,840 (5.2) |
| **Number of previous COVID-19 vaccinations** | | | | | | | | | | |
| 3 | 2,026,170 (81.1%) | 520 (2.0) | 337,780 (75.9%) | 210 (4.1) | 637,100 (78.9%) | 170 (2.2) | 318,650 (80.1%) | 100 (2.4) | 3,319,700 (80.0%) | 1,000 (2.4) |
| 4 | 426,430 (17.1%) | 840 (13.5) | 102,930 (23.1%) | 390 (22.3) | 159,910 (19.8%) | 330 (12.5) | 73,130 (18.4%) | 150 (14.2) | 762,400 (18.4%) | 1,720 (14.7) |
| 5 | 45,640 (1.8%) | 90 (14.3) | 4,140 (0.9%) | - | 10,870 (1.3%) | 30 (18.1) | 5,980 (1.5%) | 10 (13.5) | 66,630 (1.6%) | 130 (14.3) |
| **Time since previous COVID-19 vaccination** | | | | | | | | | | |
| <24 weeks | 204,840 (8.2%) | 400 (13.2) | 71,630 (16.1%) | 320 (24.0) | 82,140 (10.2%) | 190 (12.6) | 29,870 (7.5%) | 70 (13.0) | 388,480 (9.4%) | 980 (15.4) |
| ≥24 weeks | 2,293,400 (91.8%) | 1,050 (3.5) | 373,210 (83.9%) | 280 (5.2) | 725,740 (89.8%) | 340 (3.7) | 367,880 (92.5%) | 190 (4.2) | 3,760,240 (90.6%) | 1,860 (3.8) |
| **Sex** | | | | | | | | | | |
| Female | 1,335,530 (53.5%) | 720 (4.1) | 239,310 (53.8%) | 290 (8.0) | 431,690 (53.4%) | 240 (4.1) | 206,670 (52.0%) | 120 (4.5) | 2,213,200 (53.3%) | 1,370 (4.6) |
| Male | 1,162,720 (46.5%) | 730 (4.8) | 205,540 (46.2%) | 310 (9.9) | 376,190 (46.6%) | 300 (6.0) | 191,080 (48.0%) | 140 (5.7) | 1,935,530 (46.7%) | 1,470 (5.8) |
| **Age** | | | | | | | | | | |
| 18-49 years | 327,620 (13.1%) | 80 (2.1) | 50,970 (11.5%) | 10 (1.7) | 110,790 (13.7%) | 20 (1.6) | 48,280 (12.1%) | - | 537,660 (13.0%) | 110 (1.8) |
| 50-64 years | 1,233,380 (49.4%) | 190 (1.3) | 163,530 (36.8%) | 30 (1.2) | 344,670 (42.7%) | 60 (1.7) | 166,110 (41.8%) | 30 (1.6) | 1,907,690 (46.0%) | 310 (1.4) |
| 65-79 years | 713,500 (28.6%) | 470 (4.4) | 168,330 (37.8%) | 200 (7.4) | 291,840 (36.1%) | 230 (4.8) | 147,070 (37.0%) | 100 (4.5) | 1,320,740 (31.8%) | 1,000 (4.9) |
| ≥80 years | 223,740 (9.0%) | 710 (21.7) | 62,010 (13.9%) | 360 (34.3) | 60,580 (7.5%) | 220 (21.4) | 36,290 (9.1%) | 120 (22.8) | 382,620 (9.2%) | 1,410 (24.0) |
| **Ethnicity** | | | | | | | | | | |
| White | 2,172,000 (86.9%) | 1,310 (4.6) | - | - | 579,220 (71.7%) | 470 (6.1) | 384,710 (96.7%) | 250 (5.2) | 3,135,930 (84.7%) | 2,020 (4.9) |
| Asian | 94,090 (3.8%) | 30 (2.8) | - | - | 4,560 (0.6%) | 0 (0.0) | 6,220 (1.6%) | - | 104,870 (2.8%) | 30 (2.4) |
| Black | 30,270 (1.2%) | 20 (4.9) | - | - | 680 (0.1%) | 0 (0.0) | 1,010 (0.3%) | 0 (0.0) | 31,960 (0.9%) | 20 (5.4) |
| Mixed | 18,300 (0.7%) | - | - | - | 1,070 (0.1%) | 0 (0.0) | 1,460 (0.4%) | 0 (0.0) | 20,820 (0.6%) | 10 (4.0) |
| Other | 14,010 (0.6%) | - | - | - | 1,100 (0.1%) | 0 (0.0) | 1,380 (0.3%) | 0 (0.0) | 16,490 (0.4%) | 0 (0.0) |
| Unknown | 169,580 (6.8%) | 90 (4.0) | - | - | 221,250 (27.4%) | 60 (2.2) | 2,970 (0.7%) | - (-) | 393,800 (10.6%) | 150 (3.0) |
| **BMI** | | | | | | | | | | |
| <18.5 | 42,180 (1.7%) | 70 (12.3) | - | - | 6,260 (0.8%) | 20 (18.0) | 6,320 (1.6%) | - (-) | 54,760 (1.5%) | 80 (11.4) |
| 18.5-24.9 | 791,660 (31.7%) | 520 (5.0) | - | - | 101,160 (12.5%) | 130 (9.2) | 96,850 (24.4%) | 70 (5.8) | 989,670 (26.7%) | 710 (5.5) |
| 25.0-29.9 | 893,320 (35.8%) | 480 (4.1) | - | - | 467,340 (57.8%) | 220 (3.7) | 134,040 (33.7%) | 100 (5.7) | 1,494,710 (40.4%) | 800 (4.1) |
| 30.0-39.9 | 651,730 (26.1%) | 330 (3.9) | - | - | 189,230 (23.4%) | 140 (5.4) | 136,560 (34.3%) | 60 (3.8) | 977,520 (26.4%) | 530 (4.1) |
| ≥40.0 | 119,350 (4.8%) | 60 (3.6) | - | - | 43,890 (5.4%) | 30 (5.2) | 23,970 (6.0%) | 20 (5.5) | 187,200 (5.1%) | 100 (4.1) |
| **Number of QCovid risk groups** | | | | | | | | | | |
| 0 | 995,800 (39.9%) | 150 (1.2) | - | - | 412,390 (51.0%) | 90 (1.8) | 184,200 (46.3%) | 40 (1.9) | 1,592,390 (43.0%) | 280 (1.4) |
| 1 | 673,270 (26.9%) | 250 (2.8) | - | - | 257,780 (31.9%) | 150 (4.5) | 117,800 (29.6%) | 60 (3.6) | 1,048,840 (28.3%) | 460 (3.3) |
| 2 | 378,030 (15.1%) | 260 (5.1) | - | - | 90,150 (11.2%) | 140 (10.5) | 54,810 (13.8%) | 50 (6.3) | 522,990 (14.1%) | 440 (6.2) |
| 3 | 222,330 (8.9%) | 250 (8.0) | - | - | 31,470 (3.9%) | 80 (16.4) | 23,760 (6.0%) | 50 (14.5) | 277,560 (7.5%) | 370 (9.6) |
| 4 | 119,790 (4.8%) | 200 (12.2) | - | - | 11,150 (1.4%) | 50 (28.2) | 10,400 (2.6%) | 30 (19.4) | 141,340 (3.8%) | 280 (14.2) |
| ≥5 | 109,030 (4.4%) | 340 (22.6) | - | - | 4,940 (0.6%) | 20 (28.8) | 6,780 (1.7%) | 40 (39.9) | 120,750 (3.3%) | 400 (23.8) |
| **Number of BNF risk groups** | | | | | | | | | | |
| 0 | - | - | 93,920 (72.2%) | 20 (1.3) | - | - | - | - | 93,920 (72.2%) | 20 (1.5) |
| ≥6 | - | - | 36,220 (27.8%) | 160 (28.6) | - | - | - | - | 36,220 (27.8%) | 160 (28.1) |
| **Household size** | | | | | | | | | | |
| 1 person | - | - | 75,450 (17.0%) | 190 (16.2) | 265,050 (32.8%) | 270 (7.5) | 70,720 (17.8%) | 80 (8.8) | 411,220 (24.9%) | 540 (9.4) |
| 2 people | - | - | 149,730 (33.7%) | 230 (10.0) | 323,660 (40.1%) | 190 (4.3) | 161,920 (40.7%) | 110 (5.3) | 635,310 (38.5%) | 530 (5.9) |
| 3-5 people | - | - | 193,100 (43.4%) | 130 (4.7) | 209,060 (25.9%) | 60 (2.5) | 149,140 (37.5%) | 40 (2.3) | 551,300 (33.4%) | 230 (3.3) |
| 6-10 people | - | - | 22,040 (5.0%) | 20 (7.2) | 8,910 (1.1%) | - | 12,570 (3.2%) | - | 43,520 (2.6%) | 20 (3.5) |
| ≥11 people | - | - | 4,530 (1.0%) | 20 (24.5) | 1,210 (0.1%) | - | 3,410 (0.9%) | 10 (29.0) | 9,150 (0.6%) | 40 (30.1) |
| **Socioeconomic deprivation quintile** | | | | | | | | | | |
| 5th (Least) | 702,540 (28.1%) | 400 (4.3) | 111,860 (25.1%) | 140 (8.1) | 133,060 (16.5%) | 70 (3.8) | 100,710 (25.3%) | 70 (4.9) | 1,048,170 (25.3%) | 680 (4.8) |
| 4th | 604,750 (24.2%) | 330 (4.1) | 98,570 (22.2%) | 130 (8.7) | 196,780 (24.4%) | 110 (4.2) | 89,940 (22.6%) | 50 (4.5) | 990,040 (23.9%) | 610 (4.6) |
| 3rd | 512,010 (20.5%) | 280 (4.2) | 87,280 (19.6%) | 110 (8.1) | 203,790 (25.2%) | 130 (4.9) | 80,150 (20.2%) | 50 (5.0) | 883,220 (21.3%) | 570 (4.9) |
| 2nd | 404,800 (16.2%) | 240 (4.6) | 82,150 (18.5%) | 110 (8.9) | 155,360 (19.2%) | 130 (6.1) | 72,350 (18.2%) | 40 (4.7) | 714,660 (17.2%) | 510 (5.4) |
| 1st (Most) | 274,140 (11.0%) | 200 (5.9) | 64,990 (14.6%) | 110 (11.4) | 118,900 (14.7%) | 100 (6.5) | 54,610 (13.7%) | 50 (7.0) | 512,640 (12.4%) | 460 (7.0) |
| **Rural/urban area classification** | | | | | | | | | | |
| Urban | 1,873,140 (75.0%) | 1,170 (4.8) | 285,860 (64.3%) | 410 (9.5) | 655,090 (81.1%) | 450 (5.3) | 262,300 (65.9%) | 170 (5.1) | 3,076,390 (74.2%) | 2,200 (5.4) |
| Rural | 625,110 (25.0%) | 280 (3.4) | 158,990 (35.7%) | 190 (7.8) | 152,790 (18.9%) | 80 (3.8) | 135,450 (34.1%) | 80 (4.9) | 1,072,340 (25.8%) | 630 (4.4) |
| **Spikevax** | | | | | | | | | | |
| **Total** | 1,849,980 (100.0%) | 2,350 (5.9) | - | - | 1,021,330 (100.0%) | 1,090 (6.3) | 420,890 (100.0%) | 630 (7.5) | 3,292,200 (100.0%) | 4,060 (6.2) |
| **Number of previous COVID-19 vaccinations** | | | | | | | | | | |
| 3 | 1,107,250 (59.9%) | 570 (2.5) | - | - | 712,430 (69.8%) | 250 (2.3) | 266,200 (63.2%) | 120 (2.5) | 2,085,880 (63.4%) | 940 (2.4) |
| 4 | 695,460 (37.6%) | 1,670 (10.7) | - | - | 293,760 (28.8%) | 800 (13.2) | 139,270 (33.1%) | 450 (14.0) | 1,128,480 (34.3%) | 2,920 (11.8) |
| 5 | 47,270 (2.6%) | 110 (10.3) | - | - | 15,150 (1.5%) | 40 (13.5) | 15,430 (3.7%) | 60 (17.0) | 77,840 (2.4%) | 200 (12.1) |
| **Time since previous COVID-19 vaccination** | | | | | | | | | | |
| <24 weeks | 445,000 (24.1%) | 1,180 (11.6) | - | - | 210,420 (20.6%) | 590 (13.0) | 122,240 (29.0%) | 430 (14.4) | 777,650 (23.6%) | 2,200 (12.4) |
| ≥24 weeks | 1,404,980 (75.9%) | 1,170 (4.0) | - | - | 810,910 (79.4%) | 500 (3.9) | 298,650 (71.0%) | 210 (3.7) | 2,514,540 (76.4%) | 1,880 (3.9) |
| **Sex** | | | | | | | | | | |
| Female | 1,008,010 (54.5%) | 1,150 (5.3) | - | - | 578,680 (56.7%) | 580 (5.7) | 235,300 (55.9%) | 330 (6.8) | 1,821,990 (55.3%) | 2,050 (5.6) |
| Male | 841,960 (45.5%) | 1,200 (6.7) | - | - | 442,660 (43.3%) | 510 (7.0) | 185,590 (44.1%) | 310 (8.3) | 1,470,210 (44.7%) | 2,020 (7.0) |
| **Age** | | | | | | | | | | |
| 18-49 years | 171,970 (9.3%) | 70 (2.1) | - | - | 157,230 (15.4%) | 20 (0.7) | 52,410 (12.5%) | 20 (2.1) | 381,610 (11.6%) | 110 (1.6) |
| 50-64 years | 386,990 (20.9%) | 150 (2.0) | - | - | 340,350 (33.3%) | 80 (1.9) | 129,180 (30.7%) | 50 (2.2) | 856,520 (26.0%) | 290 (2.0) |
| 65-79 years | 898,940 (48.6%) | 760 (3.9) | - | - | 372,000 (36.4%) | 380 (5.3) | 163,060 (38.7%) | 200 (5.7) | 1,434,000 (43.6%) | 1,340 (4.4) |
| ≥80 years | 392,070 (21.2%) | 1,360 (15.4) | - | - | 151,750 (14.9%) | 600 (18.5) | 76,240 (18.1%) | 370 (20.5) | 620,060 (18.8%) | 2,330 (16.8) |
| **Ethnicity** | | | | | | | | | | |
| White | 1,624,620 (87.8%) | 2,130 (6.1) | - | - | 755,030 (73.9%) | 920 (7.0) | 407,790 (96.9%) | 620 (7.5) | 2,787,440 (84.7%) | 3,660 (6.5) |
| Asian | 76,920 (4.2%) | 50 (3.2) | - | - | 16,430 (1.6%) | 20 (7.1) | 6,660 (1.6%) | - | 100,010 (3.0%) | 70 (3.5) |
| Black | 21,140 (1.1%) | 20 (4.1) | - | - | 2,460 (0.2%) | 0 (0.0) | 1,080 (0.3%) | 0 (0.0) | 24,690 (0.7%) | 20 (4.0) |
| Mixed | 12,850 (0.7%) | 10 (4.5) | - | - | 2,740 (0.3%) | - | 1,480 (0.4%) | - | 17,070 (0.5%) | 10 (3.0) |
| Other | 10,070 (0.5%) | - | - | - | 2,520 (0.2%) | 0 (0.0) | 1,200 (0.3%) | 0 (0.0) | 13,780 (0.4%) | 10 (3.7) |
| Unknown | 104,380 (5.6%) | 130 (6.0) | - | - | 242,150 (23.7%) | 150 (3.9) | 2,680 (0.6%) | - | 349,210 (10.6%) | 280 (4.6) |
| **BMI** | | | | | | | | | | |
| <18.5 | 33,720 (1.8%) | 130 (17.9) | - | - | 11,120 (1.1%) | 40 (21.9) | 7,900 (1.9%) | 30 (20.5) | 52,730 (1.6%) | 200 (18.3) |
| 18.5-24.9 | 596,990 (32.3%) | 870 (6.8) | - | - | 150,420 (14.7%) | 280 (10.4) | 108,700 (25.8%) | 220 (9.8) | 856,110 (26.0%) | 1,370 (7.7) |
| 25.0-29.9 | 678,710 (36.7%) | 780 (5.3) | - | - | 601,740 (58.9%) | 490 (4.9) | 143,140 (34.0%) | 200 (7.0) | 1,423,590 (43.2%) | 1,460 (5.3) |
| 30.0-39.9 | 464,240 (25.1%) | 500 (5.1) | - | - | 214,690 (21.0%) | 240 (6.4) | 138,960 (33.0%) | 150 (5.5) | 817,880 (24.8%) | 890 (5.5) |
| ≥40.0 | 76,320 (4.1%) | 70 (4.5) | - | - | 43,370 (4.2%) | 30 (4.8) | 22,200 (5.3%) | 30 (6.3) | 141,890 (4.3%) | 130 (4.9) |
| **Number of QCovid risk groups** | | | | | | | | | | |
| 0 | 572,160 (30.9%) | 190 (1.6) | - | - | 505,380 (49.5%) | 150 (1.8) | 170,010 (40.4%) | 70 (2.1) | 1,247,560 (37.9%) | 410 (1.7) |
| 1 | 495,500 (26.8%) | 340 (3.2) | - | - | 314,300 (30.8%) | 300 (5.5) | 123,410 (29.3%) | 120 (4.8) | 933,210 (28.3%) | 760 (4.1) |
| 2 | 326,850 (17.7%) | 440 (6.3) | - | - | 121,920 (11.9%) | 270 (11.9) | 65,900 (15.7%) | 140 (10.4) | 514,670 (15.6%) | 850 (8.0) |
| 3 | 209,200 (11.3%) | 430 (9.5) | - | - | 49,630 (4.9%) | 200 (21.1) | 33,240 (7.9%) | 110 (15.6) | 292,070 (8.9%) | 740 (11.9) |
| 4 | 122,810 (6.6%) | 330 (12.3) | - | - | 19,760 (1.9%) | 100 (24.5) | 15,950 (3.8%) | 90 (25.0) | 158,510 (4.8%) | 510 (15.0) |
| ≥5 | 123,450 (6.7%) | 620 (22.8) | - | - | 10,340 (1.0%) | 70 (34.7) | 12,380 (2.9%) | 100 (38.3) | 146,170 (4.4%) | 790 (24.9) |
| **Household size** | | | | | | | | | | |
| 1 person | - | - | - | - | 367,420 (36.0%) | 510 (7.8) | 84,830 (20.2%) | 200 (11.4) | 452,250 (31.4%) | 710 (8.6) |
| 2 people | - | - | - | - | 373,660 (36.6%) | 400 (6.0) | 178,430 (42.4%) | 250 (6.8) | 552,090 (38.3%) | 650 (6.4) |
| 3-5 people | - | - | - | - | 258,060 (25.3%) | 120 (3.1) | 136,660 (32.5%) | 100 (3.8) | 394,720 (27.4%) | 220 (3.4) |
| 6-10 people | - | - | - | - | 13,160 (1.3%) | 10 (6.8) | 11,730 (2.8%) | 20 (7.6) | 24,890 (1.7%) | 30 (7.0) |
| ≥11 people | - | - | - | - | 9,030 (0.9%) | 50 (21.1) | 9,230 (2.2%) | 60 (29.2) | 18,260 (1.3%) | 100 (23.1) |
| **Socioeconomic deprivation quintile** | | | | | | | | | | |
| 5th (Least) | 498,320 (26.9%) | 610 (5.7) | - | - | 286,870 (28.1%) | 210 (4.4) | 106,820 (25.4%) | 160 (7.4) | 892,000 (27.1%) | 980 (5.6) |
| 4th | 436,320 (23.6%) | 550 (5.9) | - | - | 210,860 (20.6%) | 190 (5.4) | 82,570 (19.6%) | 100 (5.9) | 729,750 (22.2%) | 850 (5.8) |
| 3rd | 382,450 (20.7%) | 450 (5.6) | - | - | 178,160 (17.4%) | 200 (6.5) | 80,540 (19.1%) | 110 (7.1) | 641,160 (19.5%) | 770 (6.0) |
| 2nd | 307,270 (16.6%) | 400 (6.1) | - | - | 183,520 (18.0%) | 260 (8.1) | 80,940 (19.2%) | 130 (8.1) | 571,720 (17.4%) | 790 (7.0) |
| 1st (Most) | 225,620 (12.2%) | 330 (6.9) | - | - | 161,930 (15.9%) | 220 (8.1) | 70,020 (16.6%) | 130 (9.1) | 457,570 (13.9%) | 680 (7.6) |
| **Rural/urban area classification** | | | | | | | | | | |
| Urban | 1,431,850 (77.4%) | 1,890 (6.2) | - | - | 878,530 (86.0%) | 960 (6.4) | 300,660 (71.4%) | 470 (7.7) | 2,611,050 (79.3%) | 3,320 (6.4) |
| Rural | 418,120 (22.6%) | 460 (5.1) | - | - | 142,800 (14.0%) | 130 (5.2) | 120,230 (28.6%) | 160 (6.7) | 681,150 (20.7%) | 750 (5.4) |

## **Table S14:** Nation and meta, adjusted hazard ratios for vaccination, socio-demographics and clinical factors associated with severe COVID-19 outcomes under broad definitions.

|  | **England** | **NI** | **Scotland** | **Wales** | **Meta-analysis** |
| --- | --- | --- | --- | --- | --- |
| **Overall** |  |  |  |  |  |
| **Time post Autumn 2022 vaccination** | | | | | |
| 2-3 weeks | 1.00 | 1.00 | 1.00 | 1.00 | 1.00 |
| 4-5 weeks | 0.95 (0.83, 1.10) | 0.88 (0.64, 1.20) | 0.97 (0.80, 1.18) | 1.06 (0.80, 1.39) | 0.96 (0.87, 1.06) |
| 6-7 weeks | 0.98 (0.83, 1.14) | 0.82 (0.58, 1.16) | 0.94 (0.76, 1.17) | 1.01 (0.75, 1.36) | 0.95 (0.85, 1.07) |
| 8-9 weeks | 0.99 (0.84, 1.17) | 0.71 (0.49, 1.03) | 0.77 (0.61, 0.98) | 1.23 (0.91, 1.68) | 0.93 (0.83, 1.04) |
| 10-11 weeks | 0.95 (0.79, 1.13) | 0.68 (0.45, 1.03) | 0.78 (0.61, 1.00) | 1.19 (0.85, 1.66) | 0.90 (0.79, 1.02) |
| ≥12 weeks | 0.95 (0.78, 1.15) | 0.90 (0.55, 1.46) | 0.75 (0.57, 0.99) | 1.10 (0.76, 1.59) | 0.91 (0.79, 1.04) |
| **Time since previous COVID-19 vaccination** | | | | | |
| <24 weeks | 1.41 (1.32, 1.52) | 1.53 (1.26, 1.87) | 1.45 (1.29, 1.63) | 1.48 (1.24, 1.75) | 1.44 (1.36, 1.52) |
| ≥24 weeks | 1.00 | 1.00 | 1.00 | 1.00 | 1.00 |
| **Sex** | | | | | |
| Female | 1.00 | 1.00 | 1.00 | 1.00 | 1.00 |
| Male | 1.35 (1.26, 1.43) | 1.49 (1.27, 1.75) | 1.37 (1.24, 1.51) | 1.26 (1.10, 1.44) | 1.35 (1.29, 1.42) |
| **Age** | | | | | |
| 18-49 years | 1.00 | 1.00 | 1.00 | 1.00 | 1.00 |
| 50-64 years | 0.78 (0.65, 0.95) | 0.56 (0.29, 1.07) | 1.68 (1.16, 2.43) | 1.04 (0.67, 1.60) | 0.91 (0.78, 1.06) |
| 65-79 years | 1.81 (1.53, 2.15) | 2.67 (1.51, 4.74) | 4.58 (3.22, 6.53) | 1.99 (1.33, 2.98) | 2.16 (1.88, 2.48) |
| ≥80 years | 5.05 (4.25, 5.99) | 8.34 (4.67, 14.90) | 11.52 (8.02, 16.53) | 4.80 (3.16, 7.28) | 5.85 (5.09, 6.74) |
| **Ethnicity** | | | | | |
| White | 1.00 | - | - | - | 1.00 |
| Asian | 0.60 (0.48, 0.75) | - | - | - | 0.60 (0.48, 0.75) |
| Black | 0.79 (0.56, 1.10) | - | - | - | 0.79 (0.56, 1.10) |
| Mixed | 0.87 (0.56, 1.37) | - | - | - | 0.87 (0.56, 1.37) |
| Other | 0.61 (0.33, 1.13) | - | - | - | 0.61 (0.33, 1.13) |
| Unknown | 1.03 (0.90, 1.18) | - | - | - | 1.03 (0.90, 1.18) |
| **BMI** | | | | | |
| <18.5 | 2.48 (2.13, 2.89) | - | 2.47 (1.89, 3.24) | 1.83 (1.31, 2.55) | 2.38 (2.10, 2.69) |
| 18.5-24.9 | 1.29 (1.19, 1.39) | - | 1.48 (1.31, 1.68) | 1.20 (1.02, 1.41) | 1.32 (1.24, 1.40) |
| 25.0-29.9 | 1.00 | - | 1.00 | 1.00 | 1.00 |
| 30.0-39.9 | 0.98 (0.90, 1.07) | - | 1.02 (0.90, 1.15) | 0.84 (0.71, 1.01) | 0.97 (0.91, 1.04) |
| ≥40.0 | 1.15 (0.95, 1.38) | - | 1.31 (1.00, 1.71) | 1.36 (0.98, 1.89) | 1.23 (1.07, 1.41) |
| **Number of QCovid risk groups** | | | | | |
| 0 | 1.00 | - | 1.00 | 1.00 | 1.00 |
| 1 | 1.89 (1.65, 2.16) | - | 2.21 (1.89, 2.59) | 1.83 (1.44, 2.32) | 1.99 (1.81, 2.18) |
| 2 | 3.06 (2.68, 3.49) | - | 3.53 (2.99, 4.17) | 3.04 (2.39, 3.85) | 3.20 (2.91, 3.52) |
| 3 | 4.30 (3.76, 4.90) | - | 4.96 (4.14, 5.94) | 4.42 (3.44, 5.67) | 4.50 (4.08, 4.97) |
| 4 | 5.35 (4.65, 6.14) | - | 5.72 (4.61, 7.10) | 5.99 (4.57, 7.84) | 5.53 (4.97, 6.16) |
| ≥5 | 8.46 (7.44, 9.62) | - | 6.79 (5.29, 8.70) | 9.01 (6.93, 11.69) | 8.22 (7.40, 9.13) |
| **Number of BNF risk groups** | | | | | |
| 0 | - | 1.00 | - | - | 1.00 |
| 1 | - | 1.77 (1.04, 3.03) | - | - | 1.77 (1.04, 3.03) |
| 2 | - | 1.65 (0.97, 2.80) | - | - | 1.65 (0.97, 2.80) |
| 3 | - | 3.13 (1.90, 5.16) | - | - | 3.13 (1.90, 5.16) |
| 4 | - | 4.22 (2.57, 6.93) | - | - | 4.22 (2.57, 6.93) |
| 5 | - | 5.96 (3.63, 9.80) | - | - | 5.96 (3.63, 9.80) |
| ≥6 | - | 7.19 (4.41, 11.72) | - | - | 7.19 (4.41, 11.72) |
| **Household size** | | | | | |
| 1 person | - | 1.30 (1.07, 1.58) | 1.13 (1.01, 1.26) | 1.18 (1.01, 1.39) | 1.17 (1.08, 1.27) |
| 2 people | - | 1.00 | 1.00 | 1.00 | 1.00 |
| 3-5 people | - | 0.98 (0.79, 1.21) | 1.04 (0.88, 1.24) | 0.88 (0.71, 1.07) | 0.97 (0.87, 1.09) |
| 6-10 people | - | 1.54 (1.01, 2.36) | 1.25 (0.76, 2.06) | 1.36 (0.89, 2.08) | 1.39 (1.08, 1.80) |
| ≥11 people | - | 0.89 (0.54, 1.47) | 1.09 (0.80, 1.47) | 1.57 (1.21, 2.04) | 1.27 (1.05, 1.52) |
| **Socioeconomic deprivation quintile** | | | | | |
| 5th (Least) | 1.00 | 1.00 | 1.00 | 1.00 | 1.00 |
| 4th | 1.00 (0.91, 1.10) | 1.12 (0.88, 1.44) | 1.13 (0.96, 1.34) | 0.83 (0.67, 1.03) | 1.01 (0.94, 1.09) |
| 3rd | 0.95 (0.86, 1.04) | 1.01 (0.77, 1.33) | 1.25 (1.06, 1.48) | 0.96 (0.78, 1.19) | 1.01 (0.94, 1.09) |
| 2nd | 0.98 (0.89, 1.09) | 1.16 (0.89, 1.51) | 1.45 (1.24, 1.70) | 1.04 (0.85, 1.29) | 1.10 (1.02, 1.18) |
| 1st (Most) | 1.20 (1.07, 1.34) | 1.41 (1.08, 1.85) | 1.39 (1.18, 1.64) | 1.19 (0.97, 1.46) | 1.26 (1.16, 1.36) |
| **Rural/urban area classification** | | | | | |
| Urban | 1.00 | 1.00 | 1.00 | 1.00 | 1.00 |
| Rural | 0.78 (0.72, 0.85) | 0.86 (0.71, 1.04) | 0.88 (0.75, 1.03) | 1.01 (0.85, 1.19) | 0.84 (0.78, 0.89) |
| **Comirnaty** |  |  |  |  |  |
| **Time post Autumn 2022 vaccination** | | | | | |
| 2-3 weeks | 1.00 | 1.00 | 1.00 | 1.00 | 1.00 |
| 4-5 weeks | 1.05 (0.80, 1.37) | 0.87 (0.63, 1.19) | 0.85 (0.62, 1.16) | 1.29 (0.83, 2.00) | 0.97 (0.83, 1.14) |
| 6-7 weeks | 1.16 (0.90, 1.51) | 0.83 (0.58, 1.17) | 0.90 (0.65, 1.25) | 1.15 (0.72, 1.84) | 1.01 (0.85, 1.19) |
| 8-9 weeks | 1.28 (0.99, 1.65) | 0.72 (0.49, 1.04) | 0.78 (0.54, 1.12) | 1.62 (1.01, 2.60) | 1.05 (0.89, 1.25) |
| 10-11 weeks | 1.30 (1.01, 1.69) | 0.70 (0.46, 1.05) | 0.76 (0.51, 1.14) | 1.20 (0.69, 2.07) | 1.03 (0.86, 1.23) |
| ≥12 weeks | 1.23 (0.92, 1.66) | 0.89 (0.54, 1.46) | 0.85 (0.52, 1.39) | 1.42 (0.62, 3.25) | 1.09 (0.87, 1.35) |
| **Time since previous COVID-19 vaccination** | | | | | |
| <24 weeks | 1.36 (1.21, 1.54) | 1.52 (1.24, 1.85) | 1.66 (1.35, 2.04) | 1.34 (0.98, 1.82) | 1.44 (1.32, 1.58) |
| ≥24 weeks | 1.00 | 1.00 | 1.00 | 1.00 | 1.00 |
| **Sex** | | | | | |
| Female | 1.00 | 1.00 | 1.00 | 1.00 | 1.00 |
| Male | 1.35 (1.22, 1.50) | 1.46 (1.24, 1.72) | 1.65 (1.38, 1.96) | 1.34 (1.04, 1.72) | 1.42 (1.32, 1.53) |
| **Age** | | | | | |
| 18-49 years | 1.00 | 1.00 | 1.00 | 1.00 | 1.00 |
| 50-64 years | 0.71 (0.55, 0.92) | 0.56 (0.28, 1.11) | 1.09 (0.64, 1.85) | 1.11 (0.50, 2.44) | 0.77 (0.62, 0.95) |
| 65-79 years | 2.05 (1.62, 2.60) | 2.90 (1.60, 5.26) | 3.13 (1.87, 5.24) | 2.39 (1.13, 5.07) | 2.29 (1.88, 2.79) |
| ≥80 years | 6.68 (5.26, 8.47) | 8.89 (4.87, 16.23) | 8.95 (5.27, 15.20) | 7.85 (3.62, 17.03) | 7.25 (5.95, 8.83) |
| **Ethnicity** | | | | | |
| White | 1.00 | - | - | - | 1.00 |
| Asian | 0.60 (0.42, 0.87) | - | - | - | 0.60 (0.42, 0.87) |
| Black | 0.87 (0.53, 1.42) | - | - | - | 0.87 (0.53, 1.42) |
| Mixed | 0.84 (0.40, 1.76) | - | - | - | 0.84 (0.40, 1.76) |
| Other | 0.30 (0.08, 1.21) | - | - | - | 0.30 (0.08, 1.21) |
| Unknown | 1.00 (0.80, 1.24) | - | - | - | 1.00 (0.80, 1.24) |
| **BMI** | | | | | |
| <18.5 | 2.18 (1.68, 2.82) | - | 2.92 (1.72, 4.95) | 0.94 (0.41, 2.16) | 2.16 (1.73, 2.70) |
| 18.5-24.9 | 1.26 (1.11, 1.42) | - | 1.75 (1.40, 2.18) | 0.96 (0.71, 1.30) | 1.31 (1.18, 1.45) |
| 25.0-29.9 | 1.00 | - | 1.00 | 1.00 | 1.00 |
| 30.0-39.9 | 0.97 (0.84, 1.12) | - | 1.13 (0.91, 1.40) | 0.74 (0.54, 1.01) | 0.98 (0.88, 1.09) |
| ≥40.0 | 1.15 (0.87, 1.52) | - | 1.66 (1.11, 2.48) | 1.40 (0.82, 2.41) | 1.31 (1.06, 1.62) |
| **Number of QCovid risk groups** | | | | | |
| 0 | 1.00 | - | 1.00 | 1.00 | 1.00 |
| 1 | 2.01 (1.64, 2.46) | - | 1.99 (1.53, 2.58) | 1.62 (1.08, 2.42) | 1.94 (1.67, 2.26) |
| 2 | 2.93 (2.39, 3.60) | - | 3.37 (2.56, 4.42) | 2.24 (1.47, 3.43) | 2.96 (2.54, 3.44) |
| 3 | 4.08 (3.31, 5.04) | - | 4.20 (3.07, 5.77) | 4.32 (2.82, 6.63) | 4.15 (3.53, 4.88) |
| 4 | 5.38 (4.33, 6.70) | - | 6.20 (4.30, 8.94) | 4.91 (3.00, 8.05) | 5.50 (4.61, 6.55) |
| ≥5 | 7.85 (6.40, 9.63) | - | 5.71 (3.53, 9.23) | 8.75 (5.48, 13.94) | 7.64 (6.42, 9.10) |
| **Number of BNF risk groups** | | | | | |
| 0 | - | 1.00 | - | - | 1.00 |
| 1 | - | 1.83 (1.06, 3.16) | - | - | 1.83 (1.06, 3.16) |
| 2 | - | 1.71 (0.99, 2.93) | - | - | 1.71 (0.99, 2.93) |
| 3 | - | 3.19 (1.91, 5.32) | - | - | 3.19 (1.91, 5.32) |
| 4 | - | 4.36 (2.62, 7.24) | - | - | 4.36 (2.62, 7.24) |
| 5 | - | 5.92 (3.56, 9.86) | - | - | 5.92 (3.56, 9.86) |
| ≥6 | - | 7.44 (4.51, 12.26) | - | - | 7.44 (4.51, 12.26) |
| **Household size** | | | | | |
| 1 person | - | 1.28 (1.06, 1.56) | 1.43 (1.18, 1.72) | 1.15 (0.86, 1.54) | 1.32 (1.16, 1.49) |
| 2 people | - | 1.00 | 1.00 | 1.00 | 1.00 |
| 3-5 people | - | 0.97 (0.78, 1.21) | 1.07 (0.79, 1.45) | 0.74 (0.51, 1.07) | 0.95 (0.81, 1.11) |
| 6-10 people | - | 1.52 (0.98, 2.34) | 0.73 (0.18, 2.95) | 1.03 (0.45, 2.35) | 1.33 (0.92, 1.93) |
| ≥11 people | - | 0.89 (0.54, 1.47) | 1.73 (0.70, 4.28) | 1.77 (0.97, 3.23) | 1.25 (0.88, 1.78) |
| **Socioeconomic deprivation quintile** | | | | | |
| 5th (Least) | 1.00 | 1.00 | 1.00 | 1.00 | 1.00 |
| 4th | 0.93 (0.80, 1.08) | 1.10 (0.85, 1.41) | 1.03 (0.75, 1.41) | 0.85 (0.58, 1.24) | 0.96 (0.86, 1.08) |
| 3rd | 0.91 (0.78, 1.07) | 1.01 (0.77, 1.32) | 1.13 (0.84, 1.54) | 0.90 (0.61, 1.33) | 0.96 (0.85, 1.08) |
| 2nd | 0.89 (0.76, 1.05) | 1.12 (0.86, 1.46) | 1.31 (0.97, 1.78) | 0.89 (0.60, 1.34) | 0.99 (0.88, 1.12) |
| 1st (Most) | 1.15 (0.96, 1.37) | 1.44 (1.09, 1.89) | 1.34 (0.97, 1.84) | 1.33 (0.90, 1.97) | 1.26 (1.11, 1.43) |
| **Rural/urban area classification** | | | | | |
| Urban | 1.00 | 1.00 | 1.00 | 1.00 | 1.00 |
| Rural | 0.75 (0.65, 0.86) | 0.87 (0.72, 1.06) | 0.75 (0.58, 0.98) | 1.04 (0.77, 1.41) | 0.80 (0.73, 0.89) |
| **Spikevax** |  |  |  |  |  |
| **Time post Autumn 2022 vaccination** | | | | | |
| 2-3 weeks | 1.00 | - | 1.00 | 1.00 | 1.00 |
| 4-5 weeks | 1.15 (0.81, 1.64) | - | 1.06 (0.82, 1.37) | 0.93 (0.65, 1.32) | 1.05 (0.88, 1.25) |
| 6-7 weeks | 1.04 (0.67, 1.62) | - | 0.98 (0.74, 1.30) | 0.92 (0.62, 1.35) | 0.98 (0.80, 1.19) |
| 8-9 weeks | 1.24 (0.75, 2.06) | - | 0.78 (0.57, 1.05) | 1.02 (0.68, 1.53) | 0.92 (0.74, 1.14) |
| 10-11 weeks | 1.41 (0.84, 2.38) | - | 0.78 (0.57, 1.08) | 1.12 (0.73, 1.72) | 0.97 (0.77, 1.22) |
| ≥12 weeks | 1.60 (0.95, 2.71) | - | 0.73 (0.52, 1.04) | 1.00 (0.63, 1.57) | 0.95 (0.74, 1.21) |
| **Time since previous COVID-19 vaccination** | | | | | |
| <24 weeks | 1.48 (1.35, 1.62) | - | 1.38 (1.20, 1.58) | 1.60 (1.30, 1.98) | 1.47 (1.37, 1.57) |
| ≥24 weeks | 1.00 | - | 1.00 | 1.00 | 1.00 |
| **Sex** | | | | | |
| Female | 1.00 | - | 1.00 | 1.00 | 1.00 |
| Male | 1.35 (1.25, 1.47) | - | 1.24 (1.10, 1.41) | 1.23 (1.05, 1.44) | 1.30 (1.22, 1.39) |
| **Age** | | | | | |
| 18-49 years | 1.00 | - | 1.00 | 1.00 | 1.00 |
| 50-64 years | 0.94 (0.71, 1.24) | - | 2.39 (1.42, 4.03) | 1.02 (0.61, 1.72) | 1.13 (0.90, 1.41) |
| 65-79 years | 1.54 (1.21, 1.97) | - | 6.07 (3.69, 9.98) | 1.79 (1.10, 2.89) | 1.97 (1.61, 2.40) |
| ≥80 years | 3.94 (3.08, 5.03) | - | 14.34 (8.65, 23.78) | 3.81 (2.33, 6.24) | 4.81 (3.93, 5.88) |
| **Ethnicity** | | | | | |
| White | 1.00 | - | - | - | 1.00 |
| Asian | 0.59 (0.44, 0.78) | - | - | - | 0.59 (0.44, 0.78) |
| Black | 0.70 (0.44, 1.12) | - | - | - | 0.70 (0.44, 1.12) |
| Mixed | 0.88 (0.50, 1.56) | - | - | - | 0.88 (0.50, 1.56) |
| Other | 0.81 (0.40, 1.63) | - | - | - | 0.81 (0.40, 1.63) |
| Unknown | 1.05 (0.88, 1.26) | - | - | - | 1.05 (0.88, 1.26) |
| **BMI** | | | | | |
| <18.5 | 2.67 (2.21, 3.22) | - | 2.31 (1.69, 3.17) | 2.21 (1.53, 3.20) | 2.51 (2.16, 2.91) |
| 18.5-24.9 | 1.31 (1.18, 1.44) | - | 1.38 (1.19, 1.60) | 1.31 (1.08, 1.59) | 1.33 (1.23, 1.43) |
| 25.0-29.9 | 1.00 | - | 1.00 | 1.00 | 1.00 |
| 30.0-39.9 | 0.99 (0.88, 1.11) | - | 0.97 (0.82, 1.13) | 0.89 (0.72, 1.10) | 0.97 (0.89, 1.05) |
| ≥40.0 | 1.14 (0.89, 1.45) | - | 1.13 (0.79, 1.62) | 1.32 (0.87, 2.01) | 1.17 (0.97, 1.40) |
| **Number of QCovid risk groups** | | | | | |
| 0 | 1.00 | - | 1.00 | 1.00 | 1.00 |
| 1 | 1.78 (1.49, 2.12) | - | 2.32 (1.90, 2.83) | 1.94 (1.44, 2.61) | 1.99 (1.76, 2.25) |
| 2 | 3.07 (2.59, 3.64) | - | 3.60 (2.93, 4.43) | 3.42 (2.55, 4.59) | 3.30 (2.92, 3.72) |
| 3 | 4.31 (3.63, 5.11) | - | 5.32 (4.26, 6.64) | 4.47 (3.28, 6.08) | 4.63 (4.09, 5.24) |
| 4 | 5.17 (4.31, 6.19) | - | 5.52 (4.22, 7.23) | 6.46 (4.66, 8.96) | 5.46 (4.77, 6.26) |
| ≥5 | 8.55 (7.24, 10.10) | - | 7.28 (5.43, 9.78) | 9.19 (6.68, 12.64) | 8.38 (7.34, 9.56) |
| **Household size** | | | | | |
| 1 person | - | - | 1.00 (0.88, 1.15) | 1.20 (0.99, 1.45) | 1.06 (0.95, 1.19) |
| 2 people | - | - | 1.00 | 1.00 | 1.00 |
| 3-5 people | - | - | 1.02 (0.83, 1.26) | 0.95 (0.74, 1.21) | 0.99 (0.85, 1.16) |
| 6-10 people | - | - | 1.37 (0.80, 2.34) | 1.53 (0.93, 2.51) | 1.45 (1.01, 2.09) |
| ≥11 people | - | - | 1.00 (0.72, 1.38) | 1.58 (1.18, 2.13) | 1.28 (1.03, 1.60) |
| **Socioeconomic deprivation quintile** | | | | | |
| 5th (Least) | 1.00 | - | 1.00 | 1.00 | 1.00 |
| 4th | 1.05 (0.93, 1.18) | - | 1.18 (0.96, 1.43) | 0.82 (0.63, 1.06) | 1.04 (0.95, 1.14) |
| 3rd | 0.96 (0.85, 1.09) | - | 1.31 (1.07, 1.59) | 0.99 (0.77, 1.27) | 1.04 (0.94, 1.15) |
| 2nd | 1.04 (0.91, 1.18) | - | 1.51 (1.26, 1.82) | 1.10 (0.86, 1.41) | 1.16 (1.05, 1.28) |
| 1st (Most) | 1.22 (1.06, 1.40) | - | 1.40 (1.15, 1.71) | 1.15 (0.90, 1.47) | 1.25 (1.13, 1.39) |
| **Rural/urban area classification** | | | | | |
| Urban | 1.00 | - | 1.00 | 1.00 | 1.00 |
| Rural | 0.81 (0.72, 0.90) | - | 0.98 (0.80, 1.19) | 0.99 (0.81, 1.22) | 0.87 (0.80, 0.95) |

## **Figure S2:** Meta, adjusted hazard ratios for broad and strict definitions of severe COVID-19 outcomes. * England, Scotland, Wales only. † Northern Ireland only.


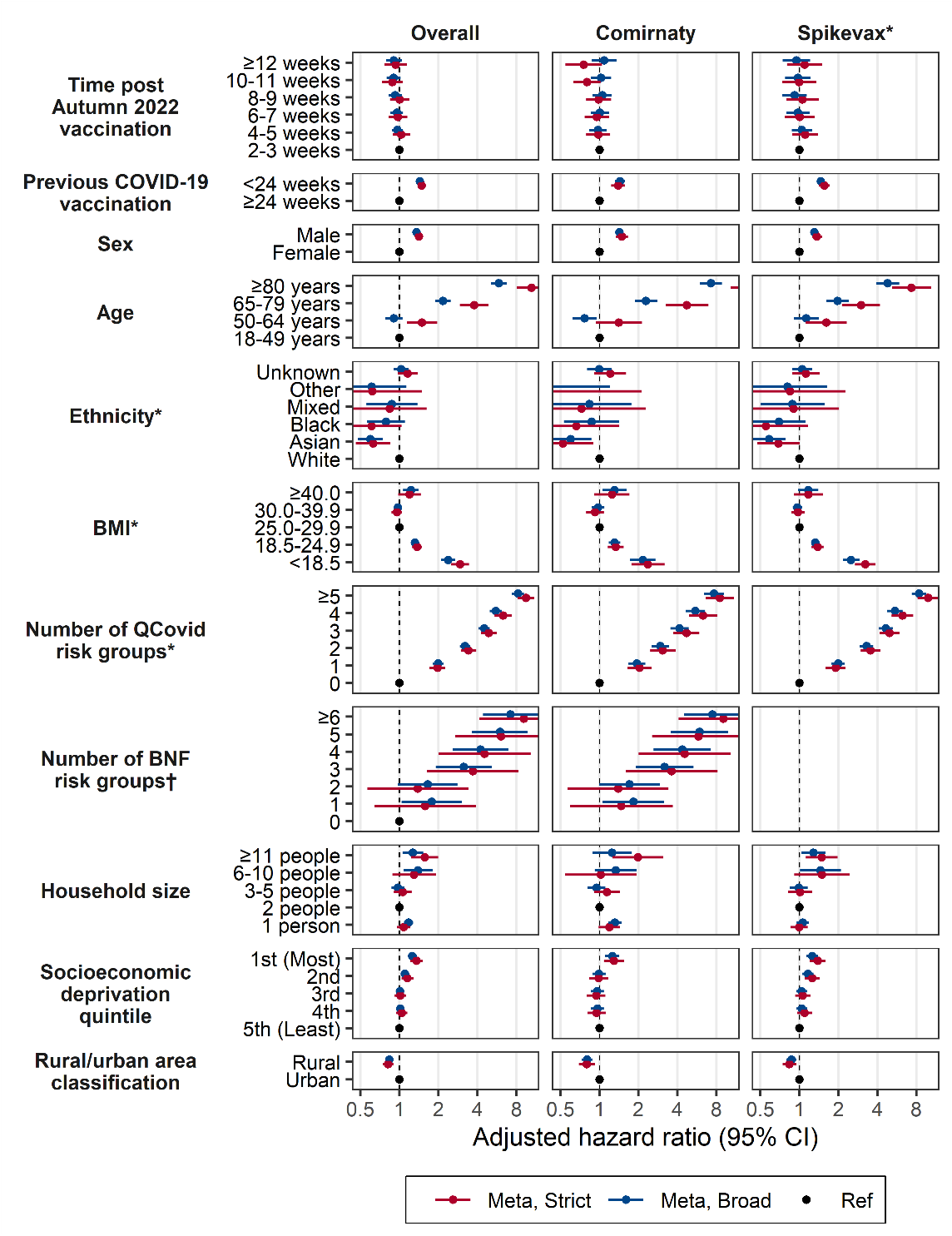


# References

1. Leston M, Elson WH, Watson C, et al. Representativeness, Vaccination Uptake, and COVID-19 Clinical Outcomes 2020-2021 in the UK Oxford-Royal College of General Practitioners Research and Surveillance Network: Cohort Profile Summary. *JMIR Public Health Surveill* 2022; **8**(12): e39141.

2. Simpson CR, Robertson C, Vasileiou E, et al. Early Pandemic Evaluation and Enhanced Surveillance of COVID-19 (EAVE II): protocol for an observational study using linked Scottish national data. *BMJ Open* 2020; **10**(6): e039097.

3. Mulholland RH, Vasileiou E, Simpson CR, et al. Cohort profile: Early pandemic evaluation and enhanced surveillance of COVID-19 (EAVE II) database. *Int J Epidemiol* 2021.

4. Vasileiou E, Simpson CR, Shi T, et al. Interim findings from first-dose mass COVID-19 vaccination roll-out and COVID-19 hospital admissions in Scotland: a national prospective cohort study. *Lancet* 2021; **397**(10285): 1646-57.

5. Simpson CR, Shi T, Vasileiou E, et al. First-dose ChAdOx1 and BNT162b2 COVID-19 vaccines and thrombocytopenic, thromboembolic and hemorrhagic events in Scotland. *Nat Med* 2021; **27**(7): 1290-7.

6. Sheikh A, McMenamin J, Taylor B, Robertson C, Public Health S, the EIIC. SARS-CoV-2 Delta VOC in Scotland: demographics, risk of hospital admission, and vaccine effectiveness. *Lancet* 2021; **397**(10293): 2461-2.

7. Lyons RA, Jones KH, John G, et al. The SAIL databank: linking multiple health and social care datasets. *BMC Med Inform Decis Mak* 2009; **9**: 3.

8. Ford DV, Jones KH, Verplancke JP, et al. The SAIL Databank: building a national architecture for e-health research and evaluation. *BMC Health Serv Res* 2009; **9**: 157.

9. Lyons J, Akbari A, Agrawal U, et al. Protocol for the development of the Wales Multimorbidity e-Cohort (WMC): data sources and methods to construct a population-based research platform to investigate multimorbidity. *BMJ Open* 2021; **11**(1): e047101.
